# Gene expression defines natural changes in mammalian lifespan

**DOI:** 10.1111/acel.12283

**Published:** 2015-02-09

**Authors:** Alexey A Fushan, Anton A Turanov, Sang-Goo Lee, Eun Bae Kim, Alexei V Lobanov, Sun Hee Yim, Rochelle Buffenstein, Sang-Rae Lee, Kyu-Tae Chang, Hwanseok Rhee, Jong-So Kim, Kap-Seok Yang, Vadim N Gladyshev

**Affiliations:** 1 Department of Bioinspired Science, Ewha Womans UniversitySeoul, 120-750, South Korea; 2 Division of Genetics, Department of Medicine, Brigham and Women's Hospital, Harvard Medical SchoolBoston, MA, 02115, USA; 3 Department of Animal Life Science, Kangwon National UniversityChuncheon, 200-701, South Korea; 4 Department of Physiology and The Sam and Ann Barshop Institute for Longevity and Aging Studies, University of Texas Health Science CenterSan Antonio, TX, 78245, USA; 5 The National Primate Research Center, Korea Research Institute of Bioscience and BiotechnologyOchang, Cheongwon, Chungbuk, 363-883, South Korea; 6 Macrogene, Inc.Geumchen-gu, Seoul, 153-781, South Korea

**Keywords:** aging, lifespan, mammals, gene expression, life-history traits

## Abstract

Mammals differ more than 100-fold in maximum lifespan, which can be altered in either direction during evolution, but the molecular basis for natural changes in longevity is not understood. Divergent evolution of mammals also led to extensive changes in gene expression within and between lineages. To understand the relationship between lifespan and variation in gene expression, we carried out RNA-seq-based gene expression analyses of liver, kidney, and brain of 33 diverse species of mammals. Our analysis uncovered parallel evolution of gene expression and lifespan, as well as the associated life-history traits, and identified the processes and pathways involved. These findings provide direct insights into how nature reversibly adjusts lifespan and other traits during adaptive radiation of lineages.

## Introduction

Extant mammals diversified dramatically, featuring more than 100-fold difference in species maximum lifespan, 100-million-fold difference in body mass and adaptations to both terrestrial and aquatic life as well as to powered flight and subterranean life. Nature has been continuously and reversibly adjusting morphology and life histories of mammals while preserving fitness, but how it does this, and what the molecular processes are involved, remains unclear. Accumulating evidence suggests a role of widespread heritable variation combined with extensive natural variation in gene expression within and between heterologous mammalian populations (Brawand *et al*., 2011). Although much of the variation is thought to evolve under neutral drift, the variation in expression of numerous genes exhibited selective constraints in diverse vertebrate species (Jordan *et al*., 2005; Whitehead & Crawford, 2006a). The extent to which evolution of gene expression contributed to certain mammalian traits is subject to debate. It is of fundamental importance to estimate the rate of gene expression changes among and within taxa and to characterize the underlying forces shaping evolution of the mammalian transcriptome.

Predominant neutrality of changes in gene expression evolving under minimal or no selective constraints was proposed as a primarily model for evolution of transcriptomes (Khaitovich *et al*., 2004a). However, the null effect of gene expression changes on phenotypes is questionable, since numerous case studies showed that gene expression alterations can result in drastic phenotypic effects, such as changes in lifespan (Yuan *et al*., 2011) and morphological differences (Beldade *et al*., 2002; Gompel *et al*., 2005). While inherited and acquired genetic variants may feature low predictable effects on phenotypes, gene expression profiles can be coordinately modified in response to environmental signals, thereby promoting specific phenotypic outcomes. Dietary interventions, such as caloric restriction (CR), which do not affect genetic structure, may control lifespan of diverse species by modulating the transcription of specific genes and remodeling metabolism (Lee *et al*., 1999; Anderson & Weindruch, 2009). Fundamental evolutionary questions, such as which forces govern the variance in transcript levels among and within distant mammalian taxa and how these variations connect genomic content and phenotypes, have only begun to be understood.

Mammals differ dramatically in their life-history strategies and, therefore, represent a model for uncovering mechanisms and underlying forces that govern evolution of phenotypes. Interspecific competition could change adaptive strategies of lineages in opposite directions, whereas environmental cues could lead to convergence in molecular mechanisms that underlie phenotypes (Losos *et al*., 1998). Lifespan, like other life-history traits, exhibits a moderate phylogenetic signal (Blomberg *et al*., 2003) that, at the molecular level, could be explained by accumulation of sequence polymorphisms and interspecies variation in transcription levels (Janecka *et al*., 2012). However, interplay between and within heritable and environmental components directing micro- and macroevolution of morphological traits has been questioned (Stearns, 2000).

It was proposed that adaptive changes in morphology and development are more evident in alterations in gene expression than in protein sequences (Carroll, 2005). Indeed, local ecological adaptations are 10-fold more likely to affect gene expression than amino acid sequences (Fraser, 2013). Therefore, studies on gene evolution at the expression level could provide further insights in phenotype evolution.

In this study, we prepared 143 RNA-seq gene expression profiles for liver, kidney, and brain and carried out comparative gene expression analyses of 33 mammalian species. By considering gene expression as a quantitative character, we examined evolution of gene expression across whole-organism life-history traits. We offer a concept of parallel evolution of mammalian life histories and gene expression profiles over evolutionary timescale and uncover the biological processes involved.

## Results and Discussion

### Analysis of gene expression to elucidate lifespan and other life-history strategies

**Fig 1:**
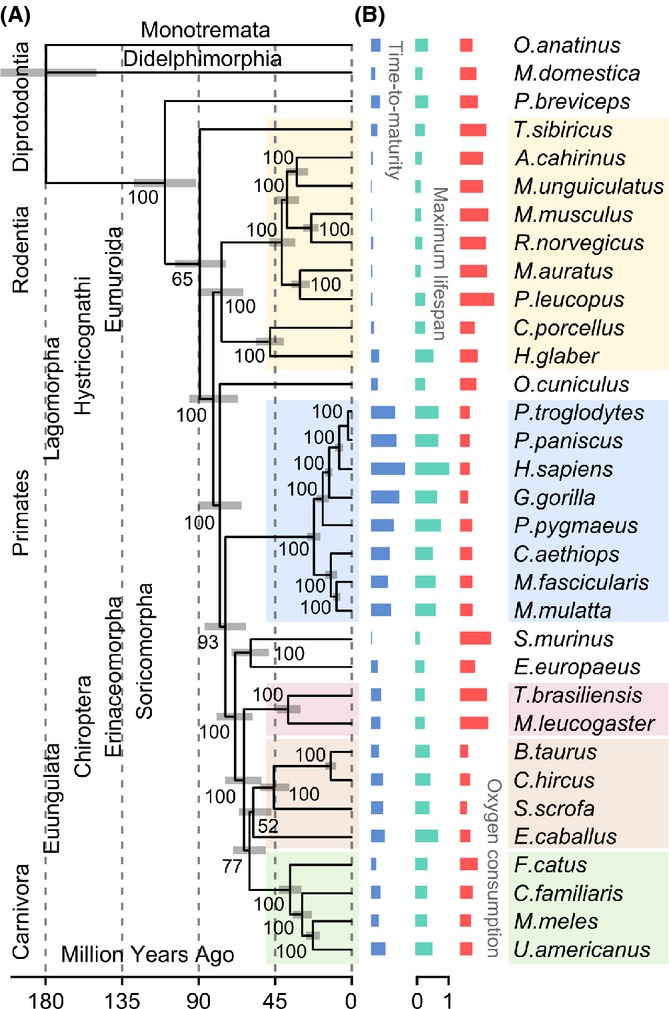
Species phylogeny and life-history traits. (A) Chronogram tree demonstrating phylogenetic relationships between mammals. Bootstrap support for the branching order of 33 species was reconstructed with 1000 randomization steps. Species divergence time is plotted as upper and lower-bounded intervals (gray bars). (B) Comparative plots of the life histories. From left to right: time to maturity, maximum lifespan, and oxygen consumption. Each bar denotes a value of life-history variable for a particular organism in standard scale.

We carried out an analysis of gene expression divergence on 33 species of terrestrial mammals of young adult age belonging to Euungulata (*n* = 4), Carnivora (*n* = 4), Chiroptera (*n* = 2), Didelphimorphia (*n* = 1), Diprotodoncia (*n* = 1), Erinaceomorpha (*n* = 1), Lagomorpha (*n* = 1), Monotremata (*n* = 1), Primate (*n* = 8), Rodentia (*n* = 9), and Soricomorpha (*n *=* *1) lineages (Table S1). Most representatives of these lineages were placental mammals (Placentalia), except for platypus (Monotremes), and opossum and sugar glider (Marsupials), and the total divergence of examined lineages corresponded to a period of ∼160 million years (Fig.1A). Evolution of these mammals yielded widespread variation in their life histories, such as time to maturity, maximum lifespan, and oxygen consumption (as a measure of basal metabolic rate, BMR) (Fig.1B). The relationship between these life histories defines a set of lineage-specific functional trade-offs and adaptive investments developed during environmental specialization. For example, most primates are characterized by extended longevity, slow growth, and reduced BMR, whereas most rodents, and in particular the muroid species (*Eumuroida*), commonly utilize opportunistic-type strategies characterized by rapid development and growth, low body mass, large litters, and short lifespan (Fig.1B). Moreover, some organisms such as representatives of *Chiroptera* and *Hystriocognathi* feature *Eumuroida*-sized species, but possess life-history attributes of larger, longer-lived mammals.

We analyzed gene expression in three organs, liver, kidney and brain, because of their easier availability, dominance of one cell type (i.e. liver), difference in metabolic functions, size of organs (which is a limitation for smaller animals), and compatibility with data from other laboratories. The majority of the examined species were represented by duplicated (52–60% of species) or triplicated (30–42% of species) biological replicates to account for within species gene expression variation (Table S1).

We generated 25–60 millions of 51-bp paired-end RNA-seq reads for each biological replicate. Reads were then mapped to genomic sequences of 21 organisms obtained from Ensembl and NCBI databases (Table S2). We used database gene model annotations and precomputed 1–1 orthologous sequence relationships for these organisms to calculate gene expression values defined as fragments per kilobase of transcript per million RNA-seq reads mapped (FPKM). Depending on species, RNA-seq read alignment efficiency varied from 55 to 99% (Table S2). For 12 species with no available genome sequences, we *de novo* assembled full-length transcriptomic contigs using RNA-seq reads (Fig. S1, Table S3), *ab initio* predicted encoded peptides (Figs S2 and S3, Table S4), and inferred 1–1 orthologous sequence relationships with database proteins (Supplementary Information, Fig. S4). We further focused our analyses on the expression of protein-coding genes with 1–1 orthologous relationship, derived from the dataset of 19 643 unique groups of sequences (Fig. S5, Table S5).

### Relationship between life histories and phylogeny of mammals

We first examined the extent to which phylogeny of the species in our study influenced life-history evolution, including gestation period, weaning time, maturation time, maximum lifespan, growth, body weight, and metabolic rate (Table S6). We used the λ model (Pagel, 1999) to test life-history variation simultaneously against randomized value (no effect of phylogeny) and against the diffusive or the Brownian motion (BM) model (neutral drift). Species phylogeny provided the null distribution, given an appropriate model of neutral evolution. The method produces a quantitative estimate of the phylogenetic signal (the extent to which correlation in traits reflects shared evolutionary history of the species) in a character, the λ parameter. Under the BM model, traits are inherited from a common ancestor and diverge linearly in a manner analogous to random walk. λ describes the proportion of variance that can be attributed to BM. The value of λ equal or close to 1 suggests a character evolution evolving under the stochastic process, whereas λ < 1 indicates departure from neutral drift. We ensured that the λ model performs well, even when the true model of trait evolution deviates from strict BM process (Supplementary Information, Figs S6 and S7).

The data showed that life-history variation of study subjects significantly departs from the diffusive model of evolution. For example, phylogeny could explain only a moderate portion of variance (λ = 0.65, *P *=* *0.02, likelihood-ratio test) in maximum lifespan (Table S7). Body weight exhibited relatively greater constraints than the other examined traits (λ = 0.39, *P *=* *0.003, likelihood-ratio test). The results indicated that with increasing genetic distance, phenotypic divergence becomes nonlinear within and between lineages.

**Fig 2:**
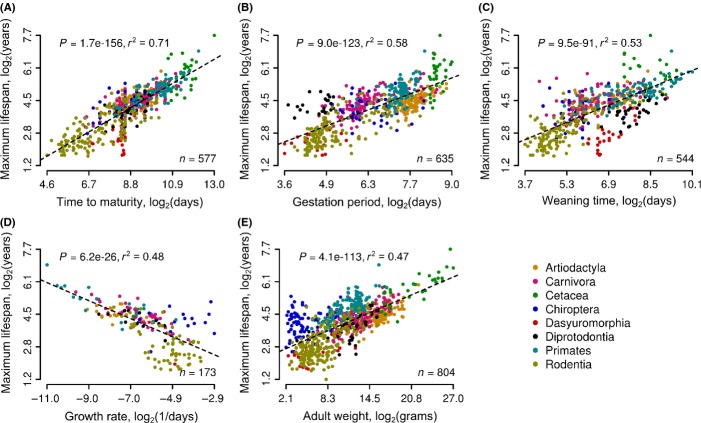
Relationship between maximum lifespan and other life histories. (A) Time to maturity. (B) Gestation period. (C) Weaning time. (D) Growth rate (Gompertz coefficient). (E) Adult weight. Number of informative species (*n*) used in the analysis is indicated in the bottom right corner of each plot. Lineages are highlighted with distinct colors (legend in the bottom right corner). Determination coefficient (*r*-squared) and statistical significance of correlation (*P*, *F*-test) are indicated at the top of each panel.

Although life-history evolution deviated from phylogeny, distinct traits preserve covariance with each other. To demonstrate this, we analyzed life-history data of ∼800 species of mammals (de Magalhaes & Costa, 2009) using nonphylogenetic regression (Fig.2). The analysis showed that, for example, maximum lifespan strongly covariates with body weight (*r*^2^ = 0.47, *P *=* *4 × 10^−113^, *F*-test), time to maturity (*r*^2^ = 0.71, *P *=* *1 × 10^−156^, *F*-test), and other examined traits (Fig.2). The data suggest that selective forces governed parallel evolution of life histories. These forces maximized fitness and interdependence between distinct traits and may also represent conserved underlying mechanisms.

### Life-history evolution shaped interspecies gene expression variation

Mammalian life histories exhibited drift and selection (Blomberg *et al*., 2003). Yet, little research has been conducted to determine the mechanisms of trait evolution in mammals because the exceptional level of life-history variation was historically influenced by complex interactions between genetics and environment.

Because life-history variation significantly departed from the model of neutral evolution, we adopted nonphylogenetic ordinary least squares (OLS) instead of phylogenetic regression to assess the relationship between transcript levels and whole-organism traits. When used improperly, the phylogenetic regression can have poor statistical performance, even under some circumstances in which the type I error rate of the method is not inflated over its nominal level (Revell, 2010). We further applied Kruskal–Wallis one-way analysis of variance as a post-hoc test to ensure that interspecies gene expression variation exceeded those within species. The analyses identified gene sets whose expression levels significantly associate (FDR-corrected *P *<* *0.05, *F*-test) with life-history variation (Fig. S8).

**Fig 3:**
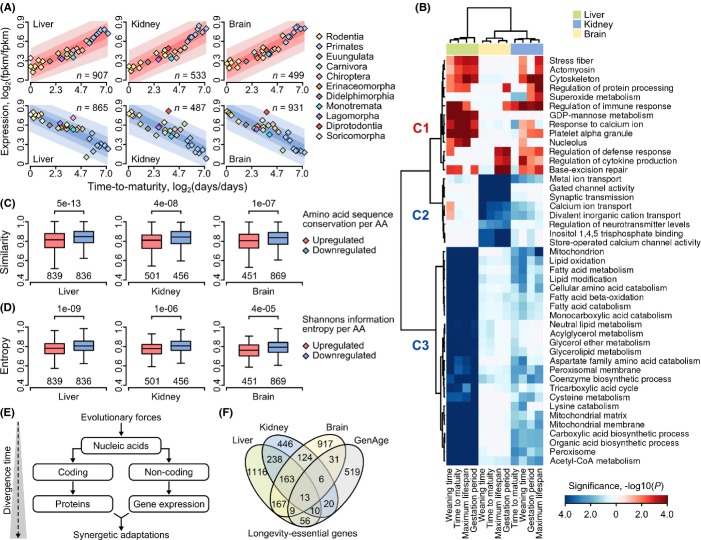
Covariance of transcript levels and life-history variation. (A) Cumulative expression profiles of transcripts associated with time to maturity. Genes positively correlated with life-history variation are plotted on the top panel (pink), and negatively correlated on the bottom panel (blue). Vertical axes denote relative FPKM (log_2_ ratio of mean FPKM of a given species to minimal mean FPKM observed among species) in standard scale. Horizontal axes denote relative time to maturity (log_2_ ratio of a given value to minimal observed value). Each rhomb on the plot denotes mean expression value of all genes (*n*) for a particular organism. Shaded areas denote 60%, 75%, and 90% upper and lower quantiles of log_2_ ratio distribution. (B) A cluster map that shows GO terms for genes associated with gradient of life-history variation. Columns on the plot correspond to a particular trait (indicated at the bottom). Rows on the plot show GO terms. Upregulated GO terms are in red. Downregulated GO terms are in blue. Magnitude of respective color denotes statistical significance of enrichment (negative logarithm of FDR-corrected *P*-value, bar at the bottom). (C) Conservation scores for molecules associated with gradient of time to maturity variation. Each panel shows distributions of per-residue similarity scores for up- (pink) and downregulated (blue) molecules for the liver, kidney, or brain. Numbers of individual orthologous groups examined in the analysis are indicated at the bottom of each bar. Significance of the difference between distributions was assessed with two-tailed Welch's *t*-tests (*P*-values at the top). (D) Shannon's information entropy for molecules significantly associated with gradient of time to maturity variation. Each panel shows distributions of per-residue entropy scores for up- (pink) and downregulated (blue) genes for the liver, kidney, or brain. (E) A model of parallel accumulation of changes in biological sequences and gene expression. (F) Overlap between gene sets associated with gradients of life-history variation and database longevity genes (mouse, fly, worm and yeast; from the GenAge dataset).

Overall, at the level of FDR-corrected *P *<* *0.05, we detected ∼5000 unique 1–1 orthologs significantly associated with 7 traits in the three organs with some overlap (381 transcripts) between organs (Table1). As an example, Fig.3A shows expression profiles of 3249 transcripts associated with gradient of time to maturity variation in liver, kidney, and brain. Although organisms examined in the study represent both laboratory and nonlaboratory populations, the sources of measurement error such as sampling and biological variations were not overdispersed compared to the estimate.

**Table 1:** Statistics on genes whose expression variation is associated with life-history variation

Variable (*P*_FDR_ < 0.05)*	Liver (*n *=* *14 679)†		Kidney (*n *=* *16 063)		Brain (*n *=* *16 424)		Combined¶
	Nb. of genes‡	% from total	Nb. of genes	% from total	Nb. of genes	% from total	
Gestation period	1017 (121)	6.9 (0.8)	588 (126)	3.7 (0.8)	926 (168)	5.6 (1.0)	2097 (75)
Weaning time	1690 (198)	11.5 (1.3)	1098 (203)	6.8 (1.3)	1453 (246)	8.8 (1.5)	3295 (193)
Body weight	506 (44)	3.4 (0.3)	116 (8)	0.7 (0.0)	549 (62)	3.3 (0.4)	1062 (11)
Growth rate	918 (116)	6.5 (0.8)	698 (173)	4.6 (1.1)	783 (123)	5.0 (0.8)	1989 (79)
Time to maturity	1740 (149)	11.9 (1.0)	998 (88)	6.2 (0.5)	1393 (95)	8.5 (0.6)	3249 (170)
Maximum lifespan	1399 (90)	9.5 (0.6)	713 (31)	4.4 (0.2)	1195 (57)	7.3 (0.3)	2683 (119)
Metabolic rate	510 (44)	3.5 (0.3)	213 (30)	1.3 (0.2)	438 (37)	2.7 (0.2)	1042 (15)
Combined§	2610 (134)	17.8 (0.9)	1753 (30)	10.9 (0.2)	2384 (97)	14.5 (0.6)	4996 (381)∥

* *P*_FDR_ denotes OLS *P*-value cut-off.

† *n* denotes total number of orthologous groups assayed in the analysis.

‡ Number of unique genes associated with trait variation and number of genes specific for a trait (in brackets).

§ Number of unique genes identified in the organ and its overlap between all traits (in brackets).

¶ Number of unique genes identified in three organs for a specific trait and interorgan overlap (in brackets).

∥ Number of unique genes identified in three organs for all traits and interorgan overlap (in brackets).

The analyses provided evidence that the interspecies variation in the expression of numerous orthologs in mammals was shaped by evolution constraints in agreement with gradient in life-history change. Life-history variation of animals in our study could explain ∼11–18% of total variability in interspecies transcript levels (Table1), whereas variability in the expression of other orthologs could be explained by drift (Figs S9 and S10, Table S8) and stabilizing constraints (Figs S11 and S12, Table S9).

It was reported previously that life-history variation governed by natural selection explains expression variation of 22% genes in marine species (Whitehead & Crawford, 2006b). Thus, gene expression evolution in vertebrates exhibits widespread selective constraints, whereas drift appears to account for less variation than expected.

**Fig 4:**
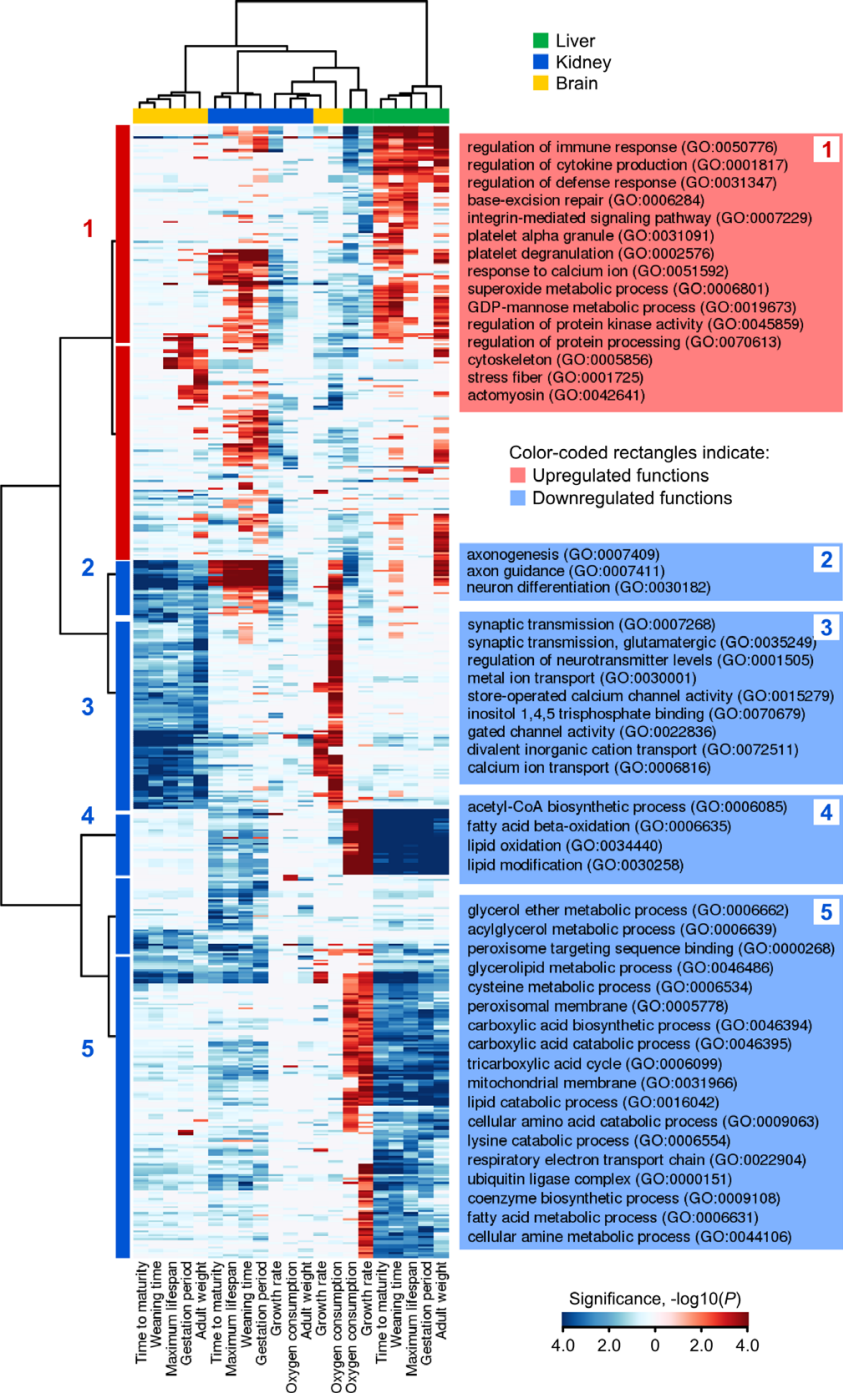
A cluster map that shows GO terms associated with gradient of life-history variation. Columns on the plot correspond to life histories (bottom). Rows show GO terms. Sub rectangles in red denote GO terms positively correlated with life-history variables. Negatively correlated GO terms are in blue. Color intensities denote statistical significance of GO term (negative logarithm of FDR corrected *P*-value, bar in the bottom right corner of plot). Life histories and GO terms were clustered using the Ward's method and Euclidean distance metric. GO terms were grouped into five clusters using constant height cutoff method (left side). Titles of representative GO terms are presented on the right side of plot (in brackets).

Gene set enrichment analysis (GSEA) revealed statistically nonrandom distribution of transcripts positively and negatively correlated with life-history traits among GO functions (Figs3B and 4, Dataset S1). In liver, we detected downregulated mitochondrial metabolic GO functions, such as metabolism of saturated and unsaturated fatty acids, and degradation of amino acids and their derivatives linked to ATP production through the TCA cycle and mitochondrial respiratory pathways (Fig.3B, cluster C3). In brain, downregulated functions included inositol and calcium-mediated signaling pathways and transmembrane channel transport (Fig.3B, cluster C2). DNA repair and defense GO functions were positively associated with gradient in lifespan variation, maturation time, and related traits (Fig.3B, cluster C1).

We detected minor overlap, which did not reach the level of statistical significance (*P* = 0.12, hypergeometric test), between individual genes shown to alter lifespan in model organisms (de Magalhaes & Costa, 2009) and genes associated with natural variation in life histories (Fig.3F). The data suggest that evolutionary changes in lifespan in mammals are associated with coordinated reprogramming of expression levels at the genomewide scale rather than with changes in expression of individual genes.

Lifespan varies among individuals of the same species, and accuracy of the estimates may be influenced by environmental conditions and sample size (Kawasaki *et al*., 2008). Thus, gene sets identified in the study may be inflated partially by quality of independent variables. We further used the gene set associated with maturation time as a representative of other sets to assess sequence conservation of the encoded proteins. The analysis showed that the downregulated orthologs were more conserved than the upregulated ones (Fig.3C). We also used Shannon' information entropy criterion (Mirny & Shakhnovich, 1999) to evaluate the number of radical amino acid substitutions and found that the number of such substitutions was also lower in the downregulated sequences (Fig.3D).

A positive relationship between amino acid substitution rate and gene expression divergence was previously reported for several species of mammals (Khaitovich *et al*., 2005). The phenomenon could be explained by nonuniform GC content in the genome defining frequencies of transitions and transversions (Misawa & Kikuno, 2011). Our data suggest parallel evolution of biological sequences and expression levels (Fig.3E) and also point out that the degree of purifying forces varies between distinct functional classes of sequences.

### Intimate relationship between life-history variation and central metabolism

**Fig 5:**
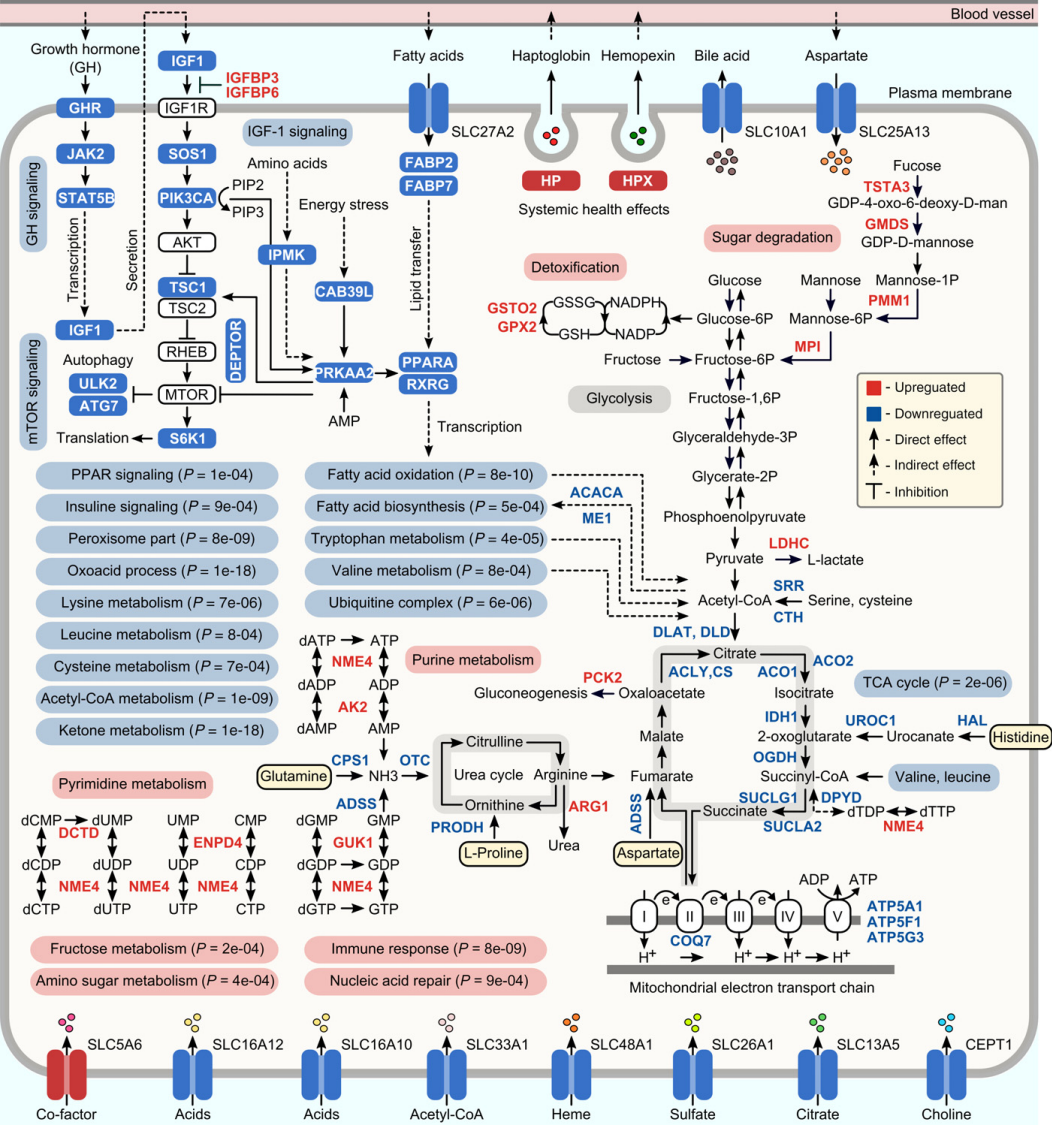
Schematic overview of genes and functions associated with gradient in lifespan variation in the liver. Rectangles in red indicate upregulated genes (FDR corrected *P *<* *0.05, *F*-test) or functions, while rectangles in blue indicate downregulated genes (FDR corrected *P *<* *0.05, *F*-test) or functions. Solid arrows denote direct effects (activation) when upstream partners interact with the targets, while dashed lines show an indirect effect (or compound entry in the pathway) occurring during downstream reactions. *P*-values denote statistical enrichment of biological pathways with significant genes (FDR corrected *P*, right-sided hypergeometric test). Refer to Dataset S1 for specific statistical details on GO functions.

**Fig 6:**
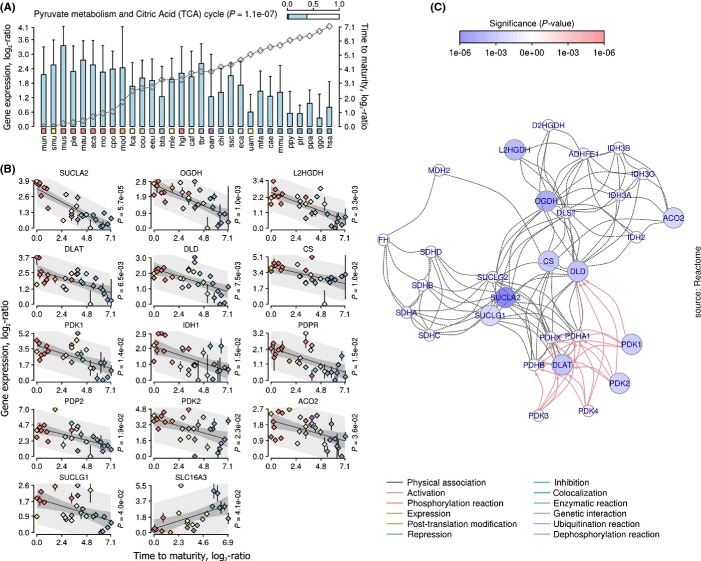
Gene expression variation associated with the TCA cycle in liver. (A) Mean FPKM of all significant genes. Error bars indicate standard deviation of the mean. Gray line is the relative value of life-history variable (time to maturity, axis on the right). Species are shown at the bottom. Color-coded rectangles distinguish lineages. Bar at the top right shows proportion of significant genes from all genes associated with this pathway. *P*-value denotes statistical enrichment (right-sided hypergeometric test). (B) Genes whose expression variation correlates with life-history variation. Vertical axis is the relative FPKM log_2_-transformed. Horizontal axis is the relative life-history variable in logarithmic space. Rhombs are the means of FPKM. Colors of rhombs distinguish lineages. Error bars show standard deviation of the mean. *P*-value denotes significance of the OLS model. Median gray line is best-fit OLS line. Shaded areas indicate observed and predicted upper (95%) and lower (5%) confidence intervals. (C) Functional interaction network. Color of nodes denotes significance of the OLS model (scale on the top). Positively correlated genes are in red. Negatively correlated genes are in blue. Color of edges denotes type of interaction (bottom).

We examined most relevant genes and biological pathways associated with life histories (Figs5 and S13). GSEA showed statistically significant label overrepresentation in the central energy metabolism combining numerous pathways such as pyruvate metabolism (*P *=* *1 × 10^−7^, hypergeometric test, Fig.6), carbohydrate degradation pathways (*P *=* *1 × 10^−5^, hypergeometric test, Fig. S14), catabolism of tryptophan (*P *=* *4 × 10^−5^, hypergeometric test, Fig. S15), lysine (*P *=* *3 × 10^−6^, hypergeometric test, Fig. S16) and valine (*P *=* *7 × 10^−7^, hypergeometric test, Fig. S17), oxidation and biosynthesis of fatty acids (*P *=* *2 × 10^−5^, hypergeometric test, Fig. S18), Ppar (*P *=* *4 × 10^−4^, hypergeometric test, Fig. S19), peroxisome (*P *=* *1 × 10^−6^, hypergeometric test, Fig. S20), Ampk (*P *=* *8 × 10^−4^, hypergeometric test, Fig. S21), growth hormone (Gh/Ghr) signaling, and others (Fig.5). Interestingly, adaptive variation in growth and lifespan of marine vertebrates was also shown to be associated with expression changes in central metabolic pathways (St-Cyr *et al*., 2008). Taking together, these data suggest fundamental relatedness of strategies governing parallel life history and transcriptome evolution in vertebrates.

Genetic interventions re-balancing expression of the effectors of these pathways are capable of modifying life-history attributes in opposite directions. In yeast, changing gene dosage for glycolytic enzyme genes resulted in variation in life-history traits such as growth and lifespan (Wang *et al*., 2010). In mice, deletion of Ghr results in increased longevity (Coschigano *et al*., 2003), whereas elevated growth hormone treatment shortens lifespan (Panici *et al*., 2010). Consistent with experimental data, natural expression divergence of Ghr negatively correlates with life-history variation of mammals (Fig.5).

Statistical analysis revealed significant relationships between life-history variation and expression levels of numerous genes involved in DNA repair, defense, and detoxification (Fig.5). We offer functional classification of these orthologs based on the activities in Datasets S2 and S3. As a less established example, haptoglobin (*Hp*) and hemopexin (*Hpx*) were reported to prevent oxidative damage resulting from hemoglobin in erythrocytes as well as protect kidney in humans (Burbea *et al*., 2004). There is positive relationship between lifespan and liver expression of *Hp* (*r*^2^ = 0.51, FDR corrected *P *=* *5 × 10^−3^, F-test) and *Hpx* (*r*^2^ = 0.38, FDR corrected *P *=* *1 × 10^−2^, *F*-test), although such relationship would also reflect species allometry and constitutive differences among homologous organs.

One would expect that changes in gene expression result in correlated downstream changes in protein levels because of positive relationship between transcript and protein levels in mammals (Schwanhausser *et al*., 2011). Therefore, rates of bioenergetic conversion and/or its efficiency may be differentially adjusted, in an organ-specific manner, in mammals in accordance with gradient of life-history changes. As a consequence, this would contribute to the levels of metabolic by-products which are thought to influence aging (Houtkooper *et al*., 2012). A higher rate of metabolism may allocate energy resources necessary for growth and reproduction in the opportunistic-type organisms such as Eumuroida species.

### Relationship between body weight and longevity traits

Body weight was shown to exhibit nonrandom association with life-history traits (de Magalhaes *et al*., 2007). When the body size is increased in natural populations, fecundity is maximized through a longer period of growth and increased lifespan, providing trade-offs between reproduction and survival mediated through body size and development time.

**Fig 7:**
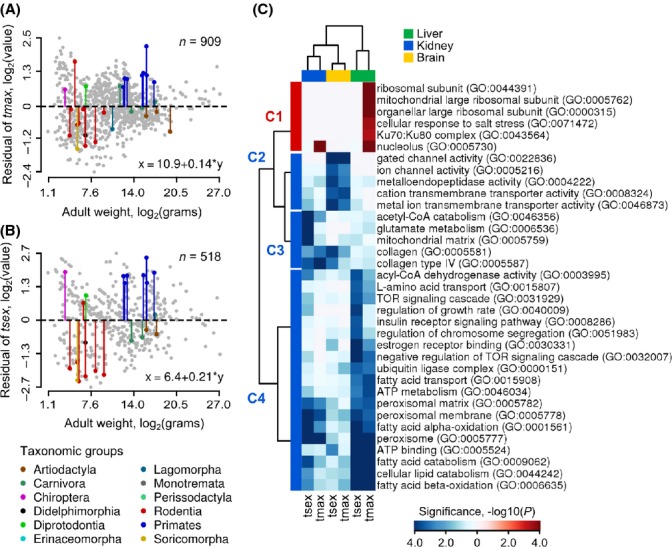
Gene expression signatures of the residual of life histories. (A) and (B) Plots show the residual of maximum lifespan (*t*_max_) and maturation time (*t*_sex_) plotted against body weight, respectively. Vertical axes are the residuals log_2_-transformed. Horizontal axes denote body weight log_2_-transformed. *n* denotes total numbers of species. Species examined in the study are highlighted with colors (legend at the bottom). Equations in the bottom right corner of each plot define linear relationship between respective life histories and body weight. (C) A cluster map that shows GO terms associated with gradient of residual variation. Columns on the plot indicate residuals of *t*_max_ and *t*_max_ (bottom). Rows show GO terms. Sub rectangles in red denote GO terms positively correlated with residual variable. Negatively correlated GO terms are in blue. Color intensities denote statistical significance of GO term (logarithm of FDR corrected *P*-value, bottom right corner).

We calculated the residuals of several traits (maturation time and maximum lifespan) to elucidate whether these variables evolved under the stochastic evolution. We excluded allometric component from the linear regression models (Fig.7A and B) and calculated the λ model on the residuals of life histories (Table S7). We found that the residual of maturation time significantly departs from neutral evolution defined by phylogeny variance structure (λ = 0.72, *P *=* *0.04, likelihood-ratio test). The analyses also demonstrated a similar relationship between phylogeny and residuals of other traits, such as maximum lifespan and oxygen consumption (Table S7). The observations provided further evidence of constraints contributing to life-history evolution in mammals, probably through interplay between drift and selection.

### Nonrandom association of gene expression with life-history residuals

We identified genes whose expression variation explains the residual of maximum lifespan and maturation time by examining statistical interaction between life histories and body weight variable in the OLS model (Fig.7, Table2). The identified genes overlapped with the gene sets associated with respective life histories, but not with body weight (Fig. S22).

**Table 2:** Statistics on genes whose expression variation is associated with the residuals of life histories

Variable (*P*_FDR_ < 0.05)*	Liver (*n *=* *14 679)†		Kidney (*n *=* *16 063)		Brain (*n *=* *16 424)		Combined¶
	Nb. of genes‡	% from total	Nb. of genes	% from total	Nb. of genes	% from total	
Maximum lifespan	659 (123)	4.5 (0.8)	469 (65)	2.9 (0.4)	366 (36)	2.2 (0.2)	1255 (43)
Time to maturity	1186 (650)	8.1 (4.4)	897 (493)	5.6 (3.1)	948 (618)	5.8 (3.8)	2428 (113)
Combined§	1309 (536)	8.9 (3.7)	962 (404)	6.0 (2.5)	984 (330)	6.0 (2.0)	2585 (126)∥

* *P*_FDR_ denotes OLS *P*-value cut-off.

† *n* denotes total number of orthologous groups assayed in the analysis.

‡ Number of unique genes associated with trait variation and number of genes specific for a trait (in brackets).

§ Number of unique genes identified in the organ and its overlap between all traits (in brackets).

¶ Number of unique genes identified in three organs for a specific trait and interorgan overlap (in brackets).

∥ Number of unique genes identified in three organs for all traits and interorgan overlap (in brackets).

**Fig 8:**
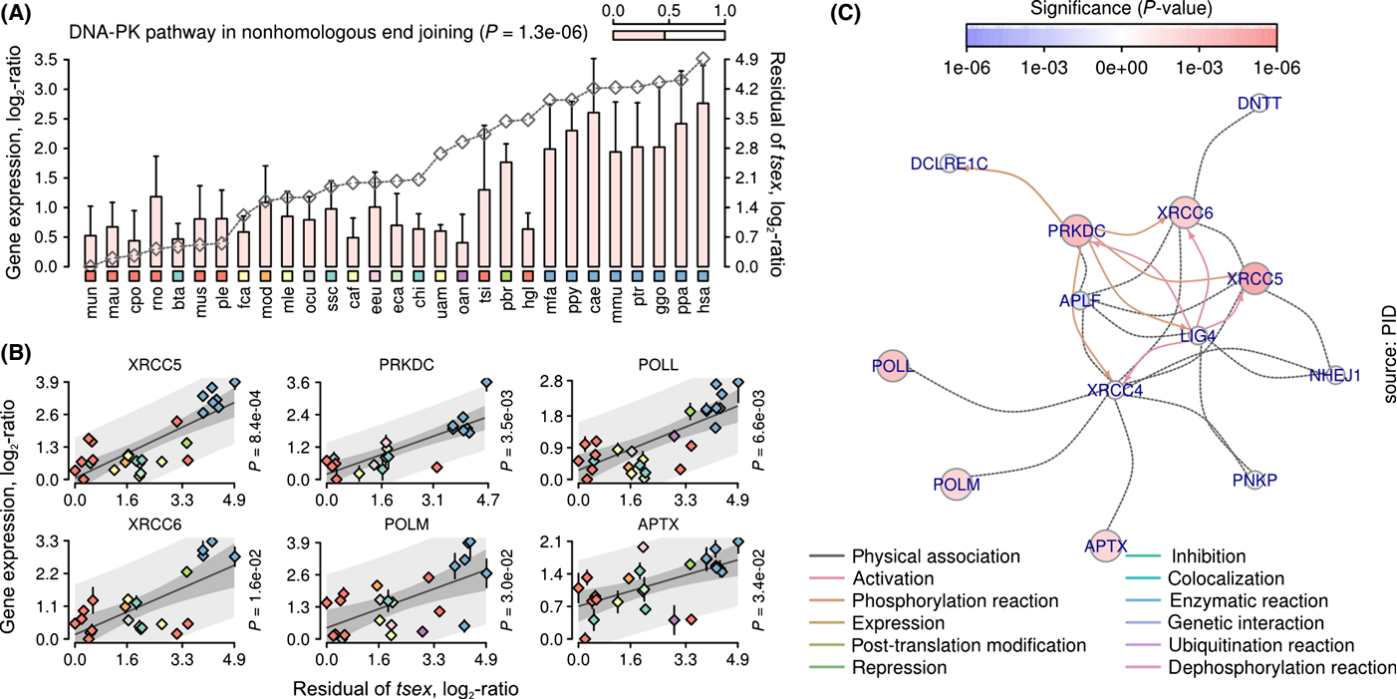
Gene expression variation associated with NHEJ positively correlates with residual of maximum lifespan and maturation time in liver. (A) Mean FPKM of all significant genes. Error bars indicate standard deviation of the mean. Gray line is the relative value of residual variable (time to maturity, axis on the right). Species are shown at the bottom. Color-coded rectangles distinguish lineages. Bar at the top right shows proportion of significant genes from all genes associated with this pathway. *P*-value denotes statistical enrichment (right-sided hypergeometric test). (B) Genes whose expression variation correlates with residual variation. Vertical axis is the relative FPKM log_2_-transformed. Horizontal axis is the residual of maturation time in logarithmic space. Rhombs are the means of FPKM. Colors of rhombs distinguish lineages. Error bars show standard deviation of the mean. *P*-value denotes significance of the OLS model. Median gray line is best-fit OLS line. Shaded areas indicate observed and predicted upper (95%) and lower (5%) confidence intervals. (C) Functional interaction network. Color of nodes denotes significance of the OLS model. Positively correlated genes are in red. Negatively correlated genes are in blue. Color of edges denotes type of interaction (bottom).

GSEA indicated label overrepresentation in the central metabolism, including mitochondrial and peroxisomal GO functions (Fig.7C, Datasets S4 and S5) and DNA repair such as nonhomologous end-joining pathway (NHEJ) (Fig.8). The latter pathway is predominantly upregulated in the primate lineage. Genes associated with NHEJ (*Xrcc5*, *Xrcc6*, *Prkdc*, etc.) mediate telomeric and chromosomal DNA repair through interaction with Wrn, whose functional impairment may promote accelerated aging syndrome and genome instability in humans (Ferguson *et al*., 2000). The processes involved in genome stability related to aging are likely to be more complex than an enhancement in simple NHEJ kinetics or telomere length, but may be related to maintenance of telomere capping (Lorenzini *et al*., 2009).

Overall, the analysis showed that the residual of life-history traits does not provide additional statistical support to explain life-history variation at the level of gene expression, although significantly reduces the associated gene sets.

## Conclusions

To our knowledge, this is the first systemic report that provides a direct evidence of widespread selection on gene expression governed by evolution of whole-organism traits in mammals. Body weight exhibited greater constraints and probably influenced evolution of other life histories, such as lifespan and maturation time. There is reciprocal interaction between forces that maximize fitness and equilibrium between life-history traits. Our analyses provide a direct evidence that expression variation of at least ∼11–18% orthologs exhibited constraints in heterologous mammalian organs and evolved in agreement with gradient of life-history variation.

Although the data may reflect allometry and local adaptive responses of species to habitat, we observed nonrandom association of gene expression variation with functions related to central energy metabolism. The magnitude of these changes was significantly correlated with gradient of life-history variation. This would make biological sense because reversible changes in species-specific developmental dynamics during chronological evolution required flexible and adjustable instruments allowing allocating cellular resources, such as energy, for growth and reproduction.

The results are intriguing because numerous case studies of aging and dietary interventions such as CR also showed gene expression alterations associated with energy metabolism, growth hormone, and stress signaling pathways (Lee *et al*., 1999; Anderson & Weindruch, 2009). CR may reduce the levels of by-products by switching glucose degradation to gluconeogenesis and lowering the rate of mitochondrial metabolism in liver (Hart *et al*., 1992), although, the magnitude of CR-inducible changes is small (Lee *et al*., 1999). An even stronger alteration in gene expression may promote longer survival of subjects, as evident from correlated variation in transcript levels and life histories in mammals. Genes and biological processes reported in the study provide a valuable resource for examination of new candidate interventions that mimic gene expression changes associated with natural changes in lifespan.

## Experimental procedures

### Animal tissue collection and RNA extraction

The description and classification of mammals examined in the study is provided in Table S1. The 143 organ samples of 23 species were obtained from various sources. The collected species belong to Euungulata (domestic cattle, domestic goat, domestic boar, horse), Carnivora (domestic dog, domestic cat, Asian badger, American black bear), Chiroptera (greater tube-nosed bat, Brazilian free-tailed bat), Didelphimorphia (short-tailed opossum), Diprotodoncia (sugar glider), Lagomorpha (old world rabbit), Primate (vervet), Rodentia (spiny mouse, guinea pig, golden hamster, Mongolian gerbil, house mouse, white-footed mouse, Norway rat, Siberian chipmunk), and Soricomorpha (house shrew). All animals were young adults and, with the exception of horse and vervet, all were males. Additional RNA-seq libraries for liver, kidney, and brain of 10 species (Primate, Erinaceomorpha, Monotremata, and Bathyergidae sp.) were obtained from NCBI Gene Expression Omnibus. The experimental protocols were approved by the Institutional Animal Care and Use Committee (IACUC) of Ewha Womans University (No. 2011-03-038,039,062,063 and 065) and Korea Research Institute of Bioscience and Biotechnology (KRIBB-AEC-12005).

The organs examined in the study represent heterogeneous tissues whose structural and cellular composition varies among species. To account for this issue and maximize sample compatibility, major parts of each organ (covering different structures/cells) were dissected and homogenized prior to RNA extraction. Given that brain is a heterogeneous organ, we sampled prefrontal cortex/frontal lobe (Primate, Euungulata, Carnivora, Diprotodoncia, Didelphimorphia, Lagomorpha sp.) or entire brain except for olfactory bulb and cerebellum (Rodentia and Chiroptera sp.). Previous studies suggested that while the cortical regions substantially differ from the cerebellum in terms of gene expression (which we account for by our sampling procedure), different regions within the cerebral cortex show small expression variation (Khaitovich *et al*., 2004b). Immediately after sacrificing, liver, kidney, and brain tissue samples were frozen in liquid nitrogen and stored at −80 °C until further use. To ensure comparability of data derived from homologous organs between species, each organ was *ground in liquid nitrogen*-cooled mortar and used for RNA extraction. Most tissue samples were prepared in biological duplicates or triplicates to ensure biological variation in gene expression (Table S1). Total RNA was extracted using RNAeasy kit Qiagen (Valencia, CA. USA) according to the manufacturer's instructions. RNA integrity was assessed using an Agilent 2100 Bioanalyzer (Lexington, MA. USA) prior to library construction.

### RNA sequencing

Sequencing libraries were prepared using the mRNA-Seq Sample Prep Kit Illumina (San Diego, CA. USA) in accordance with the manufacturer's instructions. Polyadenylated RNA was isolated using a poly-dT bead procedure, chemically fragmented, and randomly primed for reverse transcription. After second-strand synthesis, the ends of the double-stranded complementary DNA were repaired. Following 3′-end adenylation of these products, Illumina paired-end sequencing adapters were ligated to the blunt ends of the cDNA fragments. Ligated products were run on gels; 300-bp fragments were excised and then PCR-amplified (15 cycles). After column purification, quality of the resulting libraries was assessed using Agilent 2100 Bioanalyzer. Sequencing was performed on the Illumina HiSeq2000 platform generating approximately 30 million reads per sample.

### RNA-seq read mapping

Genome annotations (GTF) for 17 mammals with sequenced genomes were obtained from Ensembl, release 65. For the naked mole-rat (*Heterocephalus glaber*), long-tailed macaque (*Macaca fasciularis*), bonobo (*Pan paniscus*), and goat (*Carpa hircus*), we used GTF annotations downloaded from NCBI database (Table S2). 51-bp paired-end reads that passed the chastity filter threshold were mapped using TopHat 2.0 (Trapnell *et al*., 2012) with default parameter values, except for distance between mature pairs (*r* = 200) and the number of allowable mismatches between read and genomic sequences (*n* = 3) to account for a possible genetic variability between study and database organisms. The anchor size (i.e. the minimum aligned length spanning each of the two exons that define a splice junction) was set at 8 bp, and we allowed one mismatch on the anchor region. We filtered the read alignments accepted by TopHat to remove mapping ambiguity. To do this, we extracted the best mapping(s) for each read, based on the number of mismatches in the alignment, and selected those reads for which the best mapping was unique. Depending on species, final efficiency of RNA-seq read alignments varied from 55 to 99% (Table S2). Average gene expression levels were calculated as FPKM and normalized using Cufflinks (Trapnell *et al*., 2012). An FPKM value of 3.0 was used to filter out low abundant transcripts.

### *De novo* transcriptome assembly

Draft transcriptomes for 12 species were *de novo* assembled using Trinity (Grabherr *et al*., 2011). First, each RNA-seq reads set originating from individual biological repeat was assembled and analyzed individually (Fig. S4). As the Trinity assembler discards low coverage k-mers, no quality trimming of the reads was performed prior to the assembly. Trinity was run on the 51-bp paired-end sequences with the fixed default k-mer size of 25, minimum contig length of 200, paired fragment length of 500, and a butterfly heapspace of 25G (i.e. allocated memory). To remove redundancy, contigs that overlapped with a minimum length of 50 bp and minimum identity of 99% were merged using CAP3 (Huang & Madan, 1999) to form the organ-specific transcriptome assemblies. Finally, the assemblies from individual organs were collapsed with CAP3 for the liver, kidney, and brain to form a united reference assembly (Table S3, Figs S1 and S4).

### FPKM calculation for *de novo* transcriptomes

To calculate gene expression levels for *de novo* assembled transcripts, we developed a strategy combining *ab initio* proteome prediction, redundancy elimination followed by FPKM calculation (Fig. S4). *De novo* assembled transcriptomic contigs represent a mix of noncoding, partial, and complete cDNA sequences. The latter portion of molecules contains both start and stop signals and, therefore, can be treated as complete models in the *ab initio* protein prediction. We used augustus v2.5 software (Stanke *et al*., 2006) with default parameters optimized for eukaryote gene prediction to refine amino acid sequences encoded by reference transcriptome assemblies (Figs S2 and S4). Although *de novo* transcriptome assemblies were treated to eliminate redundant sequences, the *ab initio* predicted proteomes contained homologous sequences originating from software misassembly errors, highly homologous cDNA sequences, and transcript isoforms. To filter out redundant amino acid sequences, we applied usearch v6.0 software (Edgar, 2010) with default parameters. The final sets of amino acid sequences were encoded by nonredundant longest transcripts expressed in the liver, kidney, or brain (Fig. S3). An overview of proteome characteristics is provided in Table S4 and additionally discussed in Supplementary Information. GTF gene model annotations produced by augustus software were used for calculations of FPKM values using TopHat and Cufflinks as described above. The statistics on RNA-seq read alignments is provided in Table S2.

### Definition of orthologous genes

We obtained sequence orthologous relationships for 17 mammals with sequenced genomes from Ensembl, version 65. We considered only 1–1 orthologs in downstream analyses. Any other relationships like uncertain relationship due to the presence of paralogous sequences were excluded from the analysis. For *ab initio* predicted peptides and protein sets from the naked mole-rat (*H. glaber*), long-tailed macaque (*M. fasciularis*), bonobo (*P. paniscus*), and goat (*C. hircus*), we used inparanoid v4.1 software (Ostlund *et al*., 2009) with default parameters to refine initial 1–1 relationships with Ensembl peptides (Fig. S4). The software predicted heterogeneous relationships with distinct species in rare cases. We applied strict thresholds based on overall prediction performance (*P *=* *1, no multiple relationship was allowed) to filter out molecules with inconsistent relationships from the dataset (Fig. S4). The final dataset of orthologous groups (COG) accounted for 19 643 individual groups of sequences (Table S5, Fig. S5). The list of COG referred in the study is provided in Dataset S6.

### Expression level normalization

Initially, FPKM values of each sample were normalized against single reference sample individually using upper quartile normalization (Dillies *et al*., 2013). We then calculated log_2_ ratios centered on 0 for every pair of orthologs of two samples. The procedure was cyclically repeated for every combination of samples. The final expression values were represented by a collection of log_2_ ratios accounting for variation associated with normalization reference and biological variation between samples. Means and standardized quantiles derived from the distribution of FPKM were used in downstream analyses.

### Data quality control

Because of the scale of the project and limitations in availability of tissue samples, some inherent biases were present in our data collection and analysis, so we acknowledge them. This includes *de novo* transcriptome assemblies for organisms for which no genome is currently available, as discussed above. In addition, organisms from published databases (primarily, Primates) were used in our analysis even though some of them featured difference in read length, sequencing platform, sex (we used males, whereas some database organisms were females), and occasional alignment to closely related genomes. Nevertheless, we found that the addition of these organisms to the pipeline improved our analysis. An analysis of traits is also less sensitive to issues with individual data points.

For further data quality control, we performed a series of statistical tests to filter out any poor quality samples from the analyses. We assessed intraspecies variation by examining *CV* defined as ratio of standard deviation to mean. Comparable degree of gene expression divergence among liver, kidney, and brain was observed, which did not exceed the value of *CV* = 0.6 and with mean values centered on *CV* = 0.05 (Fig. S23). The results indicated that measurement and sampling errors as well as biological differences contribute little additional variation and that much of interspecies variation was due to distinct sources of variation.

Normalized gene expression values were examined visually and by the K-S and Welch's tests for any pair of organisms to ensure that resulting values were sampled from identical uniform distributions (Fig. S24). Any biological repeats with unusual deviation from homologous samples were excluded from the analysis. For RNA-seq libraries produced by previous studies (Brawand *et al*., 2011), we compared expression values with in-house data and examined *CV* to ensure that the downloaded data contained no instrumental or sampling errors (Fig. S25).

To verify the compatibility of FPKM calculated using conventional method (RNA-seq reads mapped to the genome) and FPKM calculated using *de novo* contigs, we assembled RNA transcripts using murine RNA-seq reads from liver, kidney, and brain. We inferred multiple orthologous relationships between *ab initio* predicted products with database sequences (excluding mouse database orthologs). We then calculated FPKM for liver, kidney, and brain and compared these values with FPKM produced from mouse genome alignments (Fig. S26). The analysis demonstrated no significant difference between FPKM produced by the two approaches (*P *=* *1, K-S test) and minor additional variation introduced by two methods (Fig. S26).

### Statistical analysis of gene expression and life histories

Analyses of gene expression and life histories were performed using OLS. Life-history variables (log_2_ ratio) were examined for nonrandom association with relative values of FPKM under assumption that the error follows Gaussian distribution:

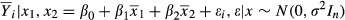
2.1where *Y*_*i*_ is the average response for gene *i*, *x*_*1*_ is the first explanatory variable, *x*_*2*_ is the covariate predictor, β_*0*_ is the intercept, ε_*i*_ is the random error, *I*_*n*_ is an *n *× *n* identity matrix, and σ^2^ determines the variance of each observation.

OLS *P*-values (*F*-test) were then corrected with the Benjamini-Hochberg FDR-controlling procedure. We further used randomization test with *n *= 10^6^ replications to ensure that observed significance exceed level that can be obtained by chance. Distribution of inter- and intraspecies expression variations were examined by Kruskal–Wallis one-way analysis of variance by ranks.

### Primary accessions

Raw sequencing data and gene expression for 143 biological samples have been deposited in Gene Expression Omnibus under accession GSE43013. All RNA-seq read data have been deposited into the Short Read Archive database. Transcriptome shotgun assembly projects and contig annotations were deposited to DDBJ/EMBL/GenBank under the following accession numbers: PRJNA182762 (*Chlorocebus aethiops*), PRJNA182765 (*Mesocricetus auratus*), PRJNA182766 (*M. leucogaster*), PRJNA182767 (*Meles meles*), PRJNA182768 (*Meriones unguiculatus*), PRJNA182769 (*Petaurus breviceps*), PRJNA182770 (*Peromyscus leucopus*), PRJNA182771 (*Suncus murinus*), PRJNA182772 (*Tadarida brasiliensis*), PRJNA182773 (*Ursus americanus*), PRJNA182705 (*Acomys cahirinus*), PRJNA183188 (*Tamias sibiricus*).

## Supporting Information

**Dataset S1**. Statistically significant GO terms for genes whose expression variation associates with life history variation.

**Dataset S2**. Biological GO functions for genes whose expression variation associates with gestation period.

**Dataset S3**. Molecular GO functions for genes whose expression variation associates with gestation period

**Dataset S4**. Statistically significant GO terms for genes whose expression variation associates with residual of life histories.

**Dataset S5**. GO functions for genes whose expression variation associates with residual of time to maturity.

**Dataset S6**. Groups of orthologous sequences assayed in the study.

**Fig. S1** *De novo* assembled transcriptomic contigs. **Fig. S2** *Ab initio* predicted peptides. **Fig. S3** Orthologous relationships inferred from *ab initio* predicted peptides. **Fig. S4** Schematic representation of methods used for *de novo* transcriptome assembly, annotation and inferring of orthologous relationships. **Fig. S5** Cluster heat map that shows quantitative representation of orthologs in COG. **Fig. S6** Influence of measurement error on the λ model. **Fig. S7** Influence of non-constant evolution rate on the λ model. **Fig. S8** Overlap of genes whose expression variation associate with life histories. **Fig. S9** The diffusive model of transcriptome evolution. **Fig. S10** Distribution of transcripts with λ > 0.95 across gene expression levels. **Fig. S11** Macroevolution of gene expression modeled with the OU process. **Fig. S12** Summary on genes and models explaining interspecies expression variation. **Fig. S13** A cluster map that shows biological pathways associated with life histories. **Fig. S14** Genes encoding enzymes of carbohydrate degradation pathway are differentially expressed in liver. **Fig. S15** Gene expression variation associated with tryptophan metabolism in liver. **Fig. S16** Gene expression variation associated with lysine metabolism in liver. **Fig. S17** Gene expression variation associated with valine metabolism in liver. **Fig. S18** Gene expression variation associated with fatty acid metabolism in liver. **Fig. S19** Gene expression variation associated with the peroxisome proliferator pathway in liver. **Fig. S20** Gene expression variation associated with peroxisome negatively correlates with life history variation in liver. **Fig. S21** Gene expression variation associated with AMPK signaling negatively correlates with life history variation in liver. **Fig. S22** Overlap of genes whose expression variation associates with life histories and residuals. **Fig. S23** Within species FPKM variation. **Fig. S24** Normalization of liver RNA-seq samples. **Fig. S25** Variation between in-house and database RNA-seq data. **Fig. S26** Variation between FPKM produced from genomic and *de novo* contig RNA-seq read alignments. **Table S1** Classification and sampling sources of 33 mammals. **Table S3** Statistics on *de novo* assembled RNA contigs for 12 mammals. **Table S4** Characteristics of *ab initio* predicted coding sequences and peptides for 12 mammals. **Table S5** The numbers of orthologs in COG. **Table S6** Life histories of 33 mammals. **Table S7** Lambda parameter estimates and associated statistics for life histories. **Table S8** Statistics for genes whose expression variation is consistent with the BM model. **Table S9** Statistics for genes whose expression variation is consistent with the OU model.

**Table S2** TopHat statistics on RNA-seq read alignments.
