# Supplementary material for: Gene expression defines natural changes in mammalian lifespan
Source: Aging Cell. 2015 Feb 9;14(3):352–65. doi: 10.1111/acel.12283 (PMC4406664; doi:10.1111/acel.12283)
Supplement: Supplementary file 7 [file acel0014-0352-sd7.pdf]

Supplementary Information

Supplementary text 3

1. *Ab initio* proteome representation 3
2. Influence of measurement error and non-constant rate of character evolution on the λ model 3
3. The diffusive model of gene expression evolution 4
4. Application of OU model to gene expression variation 4

Supplementary methods 5

Mammalian phylogeny reconstruction 5
Estimation of divergence time 5
Definition of whole-organism life history traits 6
Application of the Brownian motion model to character evolution 6
Fitting the Hansen model on gene expression 7
Amino acid sequence conservation 8
Label overrepresentation analysis 9
Databases 9

Supplementary references 9

Supplementary figures 12

1. *De novo* assembled transcriptomic contigs 12
2. *Ab initio* predicted peptides 13
3. Orthologous relationships inferred from *ab initio* predicted peptides 14
4. Schematic representation of methods used for *de novo* transcriptome assembly, annotation and inferring of orthologous relationships 15
5. Cluster heat map that shows quantitative representation of orthologs in COG 16
6. Influence of measurement error on the λ model 17
7. Influence of non-constant evolution rate on the λ model 18
8. Overlap of genes whose expression variation associate with life histories 19
9. The diffusive model of transcriptome evolution 20
10. Distribution of transcripts with λ > 0.95 across gene expression levels 21
11. Macroevolution of gene expression modeled with the OU process 22
12. Summary on genes and models explaining interspecies expression variation 23
13. A cluster map that shows biological pathways associated with life histories 24
14. Genes encoding enzymes of carbohydrate degradation pathway

are differentially expressed in liver 26
15. Gene expression variation associated with tryptophan metabolism in liver 28
16. Gene expression variation associated with lysine metabolism in liver 30
17. Gene expression variation associated with valine metabolism in liver 32
18. Gene expression variation associated with fatty acid metabolism in liver 34
19. Gene expression variation associated with the peroxisome proliferator pathway in liver 36
20. Gene expression variation associated with peroxisome negatively correlates with life history variation in liver 38
21. Gene expression variation associated with AMPK signaling negatively correlates with life history variation in liver 40
22. Overlap of genes whose expression variation associates with life histories and residuals 41
23. Within species FPKM variation 42
24. Normalization of liver RNA-seq samples 43
25. Variation between in-house and database RNA-seq data 44
26. Variation between FPKM produced from genomic and *de novo* contig RNA-seq read alignments 45

Supplementary tables 46

1. Classification and sampling sources of 33 mammals 47
3. Statistics on *de novo* assembled RNA contigs for 12 mammals 48
4. Characteristics of *ab initio* predicted coding sequences and peptides for 12 mammals 49
5. The numbers of orthologs in COG 50
6. Life histories of 33 mammals 51
7. Lambda parameter estimates and associated statistics for life histories 52
8. Statistics for genes whose expression variation is consistent with the BM model 53
9. Statistics for genes whose expression variation is consistent with the OU model 54

Ab initio proteome representation

For 12 mammalian organisms with no available genome sequences, we predicted collections of *ab initio* polypeptides expressed in the liver, kidney and brain (Table S4) based on *de novo* assembled transcriptomic contigs (Table S3). We inferred functional annotations of *ab initio* predicted proteins from sequence similarities with Ensembl peptides using Inparanoid (Fig. S4). This software has strict rules in defining of orthologous relationship between candidate sequences (i.e. size of homologous region should exceed 70% of lengths of both candidate proteins) (Ostlund *et al.*, 2009). There is a strong overlap between predicted orthologs indicating qualitative consistence of gene sets expressed in the three organs (Fig. S3). Overall, ~90% of *ab initio* predicted peptides exhibited consistent orthology relationships among 12 organisms indicating robustness of the methodology used in the study (Fig. S4). These sequences provided a snapshot of proteomes expressed in the three tissues and were used for biological analysis and classification.

Distribution of predicted peptide sizes is shown in Fig. S2. Visual examination showed that it follows non-uniform skewed distribution. Such a deviation from normality may indicate selection for polypeptides of shorter sizes over longer sequences, which is consistent with the current view of evolution of eukaryote proteomes (Kurland *et al.*, 2007; Brocchieri & Karlin *et al.*, 2005; Frith *et al*., 2006; Wang *et al.*, 2011). Recent comparative studies on eukaryote proteomes revealed evolution constraints shaping protein size distribution and selection for shorter polypeptides encoded in the genome (Kurland *et al.*, 2007; Brocchieri & Karlin *et al.*, 2005; Wang *et al.*, 2011). For example, gamma-distributed protein sizes of mouse, human and other eukaryotic species are centered on 300-400 amino acids (Frith *et al*., 2006). Small sequences (less than 100 amino acids) were reported to play important role in regulation (Frith *et al*., 2006). Therefore, short *ab intio* predicted sequences with sizes greater than 50 amino acids were also used in downstream analyses.

Influence of measurement error and non-constant rate of character evolution on the λ model

We simulated character evolution evolving under the stochastic process and examined the influence of uncorrelated variations on the λ parameter (Fig. S6). The assumption that the measurement error is independent was reasonable because of heteroskedasticity of variance between the samples. Incorporation of measurement error improved accuracy of the method because error and the λ model have exactly the same effect on the distribution of variation among species (Fig. S6).

We then simulated non-linearity in character evolution to ensure that the λ model can overcome evolution rate saturation at large phylogenetic distances (Fig. S7). We transformed branch lengths of the original phylogenetic tree using δ tree transformation approach (Pagel, 1999) aimed at generating trees spanning a range of stemminess. $\delta > 1$ increases the height of external nodes simulating character evolution on the tips of the tree, whereas $\delta < 1$ increases the height of internal nodes increasing tree stemminess that corresponds to evolution on the root of the tree. For each tree topology, we simulated ~1,800 independently evolving traits under the diffusive model of evolution, and tested the λ model for these traits on untransformed tree topology. The results indicated that variability in rates of character

evolution over phylogenetic distance had a minor effect on the λ model for traits evolving in a Brownian fashion (Fig. S7).

The diffusive model of gene expression evolution

By applying the λ model to transcript levels of common orthologs and appropriate phylogenetic subtrees, we identified a range of transcripts with λ > 0.95 in the three organs (Fig. S9A, Table S8). Because the λ model is robust to moderate differences in the model of trait evolution, we could identify the transcript sets whose expression levels diversified in agreement with phylogeny under minor or no additional constraints.

By examining the identified gene sets, we found that kidney and brain substantially overlap (more than 30% overlapped transcripts from 1,994 and 2,346 identified in the kidney and brain, respectively), whereas liver was quite distant from them (Fig. S9B). The data suggested that even at the relaxed threshold conditions (alternative hypothesis, $P < 0.05$) interspecies expression variation of ~17% transcripts in the kidney and brain and ~6% in the liver could be explained by stochastic model (Table S8), while the remaining part of the transcriptome evolved with varying degree of independence from phylogeny (Fig. S9C). The proportions are preserved across the whole range of gene expression levels with minor deviations suggesting independence of the estimates from within species gene expression variation (Fig. S10).

To evaluate whether our results were sensitive to the choice of evolutionary model, we also compared the likelihoods of the λ model with likelihoods of other models, such as single optimum Ornstein-Uhlenbeck (OU) model (Butler & King, 2004), which approximates stochastic evolution with a constraining force due to stabilizing selection. However, we did not observe significant improvements over the BM process for the reported transcript sets. Overall, the data indicated that transcript profiles experienced distinct constraints across heterologous mammalian organs and that neutral drift does not explain interspecies expression variation of numerous transcripts.

Application of the OU model to gene expression variation

We used the multi optimum OU process (Hansen model) to model ancestor states of gene expression and unravel putative stabilizing regimes operating on transcript levels. Ancestor state corresponds to a situation when gene expression variation resembles a central tendency, so that transcript levels are pulled back toward some optimal value. In the absence of selective constraints, transcript levels may further evolve under the diffusive process. However, because mammals feature unique phenotypic adaptations one would expect selective constraints that shaped interspecies gene expression variation.

We initially fitted the two-optimum OU model to gene expression variation and distinct branch segments on the tree (Fig. S11A). Starting from chronologically most ancient ancestor and ending on the most recent one, we detected varying numbers of transcripts whose expression resembled distinct clade-specific optimum. For example, transcript levels of total of 425 genes in liver, kidney and brain could

distinguish the ancestor of Rodentia from other branches suggesting widespread accumulation of gene expression variation in child branches (Fig. S11A).

We then fitted a complete combination of OU regimes to gene expression using entire phylogeny (Monotremata, Didelphimorphia and Diprotodontia were excluded because of single species representing these orders) and classified regimes with best model likelihoods explaining the observed expression variation. We found that the two-optimum OU process is the dominant regime accounting for ~73-78% of transcripts that exhibit stabilizing constraints in liver, kidney and brain (Table S9). In the case of multiple adaptive peaks, there was a more uneven distribution, as species occupying common environmental niche tend to have their own local optima. However, multi-optimum OU regimes were rare. For example, the three-optimum OU process could explain variation in the expression of ~20-25% of the transcripts that exhibited stabilizing constraints. The two-optimum OU regimes reflect clade speciation histories (Fig. S11B). Thus, bimodal gene expression changes were accumulated sequentially during speciation and adjusted unique gene sets specific for organs and lineages (Table S9).

The results could be explained by heuristic logic, because stabilization of gene expression levels assumes continuous fixation of lineage-specific transcription optimum until species occupy a common environmental niche and escape interspecific competition. Diverse lineages feature unique environmental adaptations, and stabilizing regime acting on expression levels of specific transcripts could contribute to these adaptations.

Supplementary Methods

Mammalian phylogeny reconstruction

We aligned 424 common protein orthologs of 33 mammals with Muscle v3.8 (Edgar, 2004) and produced a concatenated gap-free alignment with Gblocks v0.91 (Castresana, 2000). Respective genes were than examined using PAML (Yang, 2007) for positive selection (M1a and M2a hypotheses) to validate that the encoded products exhibited nearly neutral evolution across branches. Species phylogeny was than reconstructed with the Neighbor-Joining method. The reliability of branching patterns was assessed in 1,000 bootstrapping replications using Mega 5.1 (Kumar *et al.*, 2008) and PAML software.

Estimation of divergence time

For the concatenated multiple amino acid sequence alignment, we used a calibration range 150-210 Mya for the divergence time. This range appears to be the most reliable for the divergence date between human and platypus, the most distant species in the dataset (Kumar & Subramanian, 2002). To calculate divergence time, we used PAML and MCMCTree (Yang, 2007) utilizing a Bayesian phylogenetic approach. The method accepts an upper and a lower bound on calibration points. Mammalian sequence evolution exhibits large rate differences within and between lineages (Kumar & Subramanian, 2002). Therefore, a global clock cannot be assumed for complex phylogenies. We used the independent substitution rate model in the reconstruction analysis. Divergence times were calculated using Whelan and Goldman (WAG) amino acid substitution matrix (Whelan & Goldman, 2005).

Definition of whole-organism life history traits

The data on life histories were collected from the AnAge database (de Magalhaes & Costa, 2009) and literature in case of rate of oxygen consumption (Heusner, 1991; Clarke *et al.*, 2010; White & Seymour, 2003) and were cross-validated by independent sources such as PanTHERIA database. Overall, 7 life history traits were examined in the study (Table S5). AnAge database internally traces the quality of population parameters with the number of subjects that were under observation and by the quality of data source. Poorly rated data (“tiny” sizes of populations or “unacceptable” quality) were excluded from the analyses.

Maximum lifespan (*tmax*) is the maximum time interval from birth to death documented for a given population of organisms within species. The accuracy of *tmax* depends on sample size being under observation and, therefore, the precision of the estimate may vary among lineages. The best estimate of *tmax* is available for human populations. *tmax* exhibits strong relationships with other traits such as time to maturity (de Magalhaes *et al.*, 2007) estimated with greater precision for multitude species.

Oxygen consumption is the volume of oxygen consumed by an individual per an hour and, therefore, defines the intensity of resting (basal) metabolic rate (BMR). There is considerable correlation of BMR with body weight (White & Seymour, 2003). Thus, oxygen consumption with subtracted body weight component provides phylogeny-unrelated estimate of BMR.

Application of the Brownian motion model to character evolution

We used the BM model, developed for analyses of numerical traits evolving along a given phylogenetic pattern, to model evolution of life histories. The BM theory assumes a linear accumulation of changes in a numerical trait over time (Boettiger *et al.*, 2012; Freckleton & Harvey, 2006). For particular values at ancestral nodes, the likelihood (*L*) of observing a set of phenotypic data for a single character at the tips of ultrameric phylogenetic tree can be represented as:

$$L = \prod \frac{1}{\sqrt{2\pi(\upsilon_{n1} + \upsilon_{n2})}} \exp\left[-\frac{(x_{n1} - x_{n2})^2}{2(\upsilon_{n1} + \upsilon_{n2})} \right]$$

where *L* is the product over all nodes on the tree; *n* indicates a particular node and N is the total number of nodes (Freckleton & Harvey, 2006). The term (x_{n1}-x_{n2}) is the difference in trait values at two descendents of each node *n*. v_{n1} and v_{n2} are variance values derived from the branch lengths of the phylogeny in units of expected amount of time available for phenotypic change along branches of the tree.

Several approaches have been proposed for estimation of the likelihood parameter (Blomberg *et al.*, 2003). We constructed a phylogenetic covariance from the phylogenetic tree to evaluate the BM process and calculated parameter lambda (λ). λ is a branch length scaling parameter that allowed to range from 0 to 1 (Pagel, 1999). With the tree in this variance-covariance matrix form, λ scales the off-diagonal elements of the matrix by the amount of coefficient. It moves from 1 to 0 the shorter the internal branches. The final tree is a star-like with all branches emanating from a common node. When λ = 1, there was no transformation that corresponds to the BM of the trait along the phylogenetic pattern. When

λ = 0, co-variances are zero, corresponding to random noise. A star-like tree reflects less phylogenetic structure, that is, less phylogenetic signal.

To test the significance of the λ model we estimated log-likelihoods of the BM model for original (where it is allowed to take its maximum value) and star-like topologies and calculated the ratio between log-likelihoods of these models and the λ model. The probability that the observed value of λ differs from random distribution and the probability of deviation from the BM process were then estimated using chi-squared distributions (Pagel, 1999).

We accounted for a measurement error in the data (Supplementary Information, Fig. S6). The sources of measurement error include sampling variation, variation related to age, sex, season, etc. Although estimating the total measurement error (e.g., the variation among all populations of a species) is unrealistic, incorporating the measurement error associated with the observations provides substantial improvement to the method (Ives *et al.*, 2007).

Under the λ model the multivariate distribution of tip values is $x \sim \sigma^2 C_\lambda$, where C_λ is an $n \times n$ matrix for *n* species containing, in the diagonal, the height of each species above the root, and in each off-diagonal element $C_\lambda(i,j)$, the height above the root node of the most common recent ancestor of species *i* and *j* multiplied by the coefficient λ. With measurement error $x \sim \sigma^2 C_\lambda + E$, where E is a diagonal matrix containing the square of the estimation error for each species and $E \sim \sigma^2_m M$. The variance due to measurement error M of trait *x* for species *i* is $\sigma^2_m m_{ij}$ where m_{ij} is the *i*-th diagonal element of M. Therefore, the distribution of tip values among species is:

$$x = a + \varepsilon + \eta, \varepsilon + \eta \sim \sigma^2 C_\lambda + \sigma_m^2 M$$

where *x* is a N×1 vector containing the observed values of the trait, *a* is a scalar giving the expected value of the trait, *ε* is a N×1 vector of zero-mean error terms depicting the evolutionary variance of the trait among species, and *η* is the N×1 vector of errors associated with measurement (Ives *et al.*, 2007).

Fitting the Hansen model on gene expression

Adaptive evolution of trait X (transcription levels of each single gene for a set of species) was modeled as an OU process with stepwise Akaike Information Criterion (Ingram *et al.*, 2013). In OU process, lineages in distinct selective regimes are limited to certain optima of X assuming that X follows a multivariate normal distribution. Under the OU process, a continuous character evolves as following:

$$dX(t) = \alpha[\theta - x(t)]d(t) + \beta dW(t)$$

where β defines the magnitude of the diffusion process over time interval d*t*, the Brownian rate parameter. dW(*t*) is Wiener process (noise) following uniform distribution. Parameter α defines the strength of adaptive evolution attracting local optimum to value θ.

The regime shifts were sequentially added to the initial OU model in which the entire tree is in a single regime. Maximum likelihood was than calculated to estimate OU parameter values and likelihoods of the model (*L*). The performance of each new model was estimated using the Akaike information criterion (AIC) (Boettiger *et al.*, 2012; Harmon *et al.*, 2010):

$$AIC_c = -2\log(L) + 2p + \left(\frac{2p(p+1)}{N-p-1}\right)$$

where N is number of trait values and p is number of parameters in the Hansen model. The improvements of each new model was defined as $\Delta AIC_{c(i)} = AIC_{c(I\ )} - AIC_{c(j)}$, the difference between AIC of the *i*-th model from the *j*-th one. Monte Carlo simulations were also used to determine whether each *i*-th model had statistically significant improvement over *j*-th treated as the null model (Boettiger *et al.*, 2012). Fitting of candidate models was repeated until no candidate model exceed the criterion $\Delta AIC_{c(i)} < \Delta AIC_{c(j)}$, indicating that the new model did not provide significant improvements over the existing one. The regimes corresponding to the best models were retained through the iterations.

Candidate regimes were then collapsed by evaluating all pairwise combinations of regimes *i* and *j*, and calculating OU parameters for the model. $\Delta AIC_{c(ij)}$ was than calculated for each of k(k-1)/2 candidate models to determine which model meets the criterion $\Delta AIC_{c(ij)} < \Delta AIC_c$. The criterion indicates that the model was improved when regimes *i* and *j* were collapsed. The convergence of the final model was defined as $\Delta k = k - k'$, representing the simplification of convergent model (decrease in the number of regimes) over the collapsed models (Ingram *et al.*, 2013).

Amino acid sequence conservation

The analysis of conservation aims to identify positions in a protein sequence which are conserved within each orthologous population that acquires this sequence. Such analysis also provides information about selection pressure acting across diverse protein groups and classes.

Conservation of a particular amino acid residue is defined as the average of the similarity scores of all pairwise comparisons for that position in the alignment, whereas the similarity score between any two residues is the score value between these residues in the chosen substitution matrix. An average conservation score for a group of amino acid sequences is a per residue similarity adjusted by the number of informative amino acids in the alignment.

The degree of evolutionary conservation within a family of homologous sequences was measured by Shannon's information entropy for a particular orthologous group:

$$S(l) = -\sum_{i=1}^{6} P_i(l)\log P_i(l)$$

where $P_i(l)$ is the frequency of each of the six classes *i* of residues at position *l* in the multiple sequence alignment (Mirny & Shakhnovich, 1999). The six classes of residues are: aliphatic (AVLIMC), aromatic (FWYH), polar (STNQ), positive (KR), negative (DE), and special (reflecting their special conformational properties) (GP). In addition to conservation, *S(l)* also reflects the level of amino acid substitutions between and within homologous sequences. A low value of the intrafamily conservation *S(l)* indicates that the particular amino acid position was under evolutionary pressure to keep a certain type of residue.

An average level of amino acid sequence diversity per amino acid residue for a particular orthologous group of sequences can be calculated as follows:

$$S = \frac{\sum_{k=1}^{N} S(l)}{N}$$

where *S(l)* is an information entropy for *l*-th residue and *N* is the number of informative amino acids in the alignment. The union {S_1, S_2…S_k} provides an estimate of evolution conservation and divergence for a custom class of *k* orthologs.

Label overrepresentation analysis

Label overrepresentation (gene set enrichment) analysis for functional annotation, ontology and pathways were done using standard right-sided hypergeometric test employing all genes on the array as denominator and genes under interest as numerator (Huang da *et al.*, 2009). *P*-values were corrected by the Benjamini-Hochberg FDR-controlling procedure.

Databases

For pathway and gene ontology analyses, we used latest builds of CPDB (Kamburov *et al.*, 2009) and gene ontology consortium databases (Ashburner *et al*., 2000), respectively. CPDB is a comprehensive database of biochemical pathways that accumulates data from KEGG, Reactome, HumanCyc and related sources. Electronically inferred GO annotations (RCA, IEA, NR and ND codes) were excluded from gene ontology enrichment analyses. Protein-protein interactions were obtained from CPDB and STRING 9.0 (Szklarczyk *et al*., 2010). Only highly confident interactions as defined by the original sources were used in biological network analyses.

Supplementary references

Ashburner M, Ball CA, Blake JA, Botstein D, Butler H, Cherry JM, Davis AP, Dolinski K, Dwight SS, Eppig JT, Harris MA, Hill DP, Issel-Tarver L, Kasarskis A, Lewis S, Matese JC, Richardson JE, Ringwald M, Rubin GM, Sherlock G (2000). Gene ontology: tool for the unification of biology. The Gene Ontology Consortium. Nat Genet. 25, 25-29.

Blomberg SP, Garland T, Jr., Ives AR (2003). Testing for phylogenetic signal in comparative data: behavioral traits are more labile. Evolution. 57, 717-745.

Boettiger C, Coop G, Ralph P (2012). Is your phylogeny informative? Measuring the power of comparative methods. Evolution. 66, 2240-2251.

Brocchieri L, Karlin S (2005). Protein length in eukaryotic and prokaryotic proteomes. Nucleic acids research. 33, 3390-3400.

Butler MA, King AA (2004) Phylogenetic comparative analysis: a modeling approach for adaptive evolution. American Naturalist. 164, 683-695.

Castresana J (2000). Selection of conserved blocks from multiple alignments for their use in phylogenetic analysis. Mol Biol Evol. 17, 540-552.

Clarke A, Rothery P, Isaac NJ (2010). Scaling of basal metabolic rate with body mass and temperature in mammals. J Anim Ecol. 79, 610-619.

Edgar RC (2004). MUSCLE: multiple sequence alignment with high accuracy and high throughput. Nucleic Acids Res. 32, 1792-1797.

Freckleton RP, Harvey PH (2006). Detecting non-Brownian trait evolution in adaptive radiations. PLoS Biol. 4, e373.

Frith MC, Forrest AR, Nourbakhsh E, Pang KC, Kai C, Kawai J, Carninci P, Hayashizaki Y, Bailey TL, Grimmond SM (2006). The abundance of short proteins in the mammalian proteome. PLoS genetics. 2, e52.

Heusner AA (1991). Size and power in mammals. J Exp Biol. 160, 25-54.

Harmon LJ, Losos JB, Jonathan Davies T, Gillespie RG, Gittleman JL, Bryan Jennings W, Kozak KH, McPeek MA, Moreno-Roark F, Near TJ, Purvis A, Ricklefs RE, Schluter D, Schulte Ii JA, Seehausen O, Sidlauskas BL, Torres-Carvajal O, Weir JT, Mooers AO (2010). Early bursts of body size and shape evolution are rare in comparative data. Evolution. 64, 2385-2396.

Huang da W, Sherman BT, Lempicki RA (2009). Bioinformatics enrichment tools: paths toward the comprehensive functional analysis of large gene lists. Nucleic Acids Res. 37, 1-13.

Ingram T, Mahler DL (2013). SURFACE: detecting convergent evolution from comparative data by fitting Ornstein-Uhlenbeck models with stepwise Akaike Information Criterion. Meth Ecol Evol. 4, 416-425.

Ives AR, Midford PE, Garland T, Jr. (2007). Within-species variation and measurement error in phylogenetic comparative methods. Syst Biol. 56, 252-270.

Kumar S, Nei M, Dudley J, Tamura K (2008). MEGA: a biologist-centric software for evolutionary analysis of DNA and protein sequences. Brief Bioinform. 9, 299-306.

Kumar S, Subramanian S (2002). Mutation rates in mammalian genomes. Proc Natl Acad Sci U S A. 99, 803-808.

Kurland CG, Canback B, Berg OG (2007). The origins of modern proteomes. Biochimie. 89, 1454-1463.

Kamburov A, Wierling C, Lehrach H, Herwig R (2009). ConsensusPathDB--a database for integrating human functional interaction networks. Nucleic Acids Res. 37, D623-628.

de Magalhaes JP, Costa J, Church GM (2007). An analysis of the relationship between metabolism, developmental schedules, and longevity using phylogenetic independent contrasts. J Gerontol A Biol Sci Med Sci. 62, 149-160.

de Magalhaes JP, Costa J (2009). A database of vertebrate longevity records and their relation to other life-history traits. J Evol Biol. 22, 1770-1774.

Mirny LA, Shakhnovich EI (1999). Universally conserved positions in protein folds: reading evolutionary signals about stability, folding kinetics and function. J Mol Biol. 291, 177-196.

Ostlund G, Schmitt T, Forslund K, Kostler T, Messina DN, Roopra S, Frings O, Sonnhammer EL (2009). InParanoid 7: new algorithms and tools for eukaryotic orthology analysis. Nucleic Acids Res. 38, D196-203.

Pagel M (1999). Inferring the historical patterns of biological evolution. Nature. 401, 877-884.

Szklarczyk D, Franceschini A, Kuhn M, Simonovic M, Roth A, Minguez P, Doerks T, Stark M, Muller J, Bork P, Jensen LJ, von Mering C (2010). The STRING database in 2011: functional interaction networks of proteins, globally integrated and scored. Nucleic Acids Res. 39, D561-568.

Wang M, Kurland CG, Caetano-Anolles G (2011). Reductive evolution of proteomes and protein structures. Proc Natl Acad Sci U S A. 108, 11954-11958.

Whelan S, Goldman N (2001). A general empirical model of protein evolution derived from multiple protein families using a maximum-likelihood approach. Mol Biol Evol. 18, 691-699.

White CR, Seymour RS (2003). Mammalian basal metabolic rate is proportional to body mass2/3. Proc Natl Acad Sci U S A. 100, 4046-4049.

Yang Z (2007). PAML 4: phylogenetic analysis by maximum likelihood. Mol Biol Evol. 24, 1586-1591.

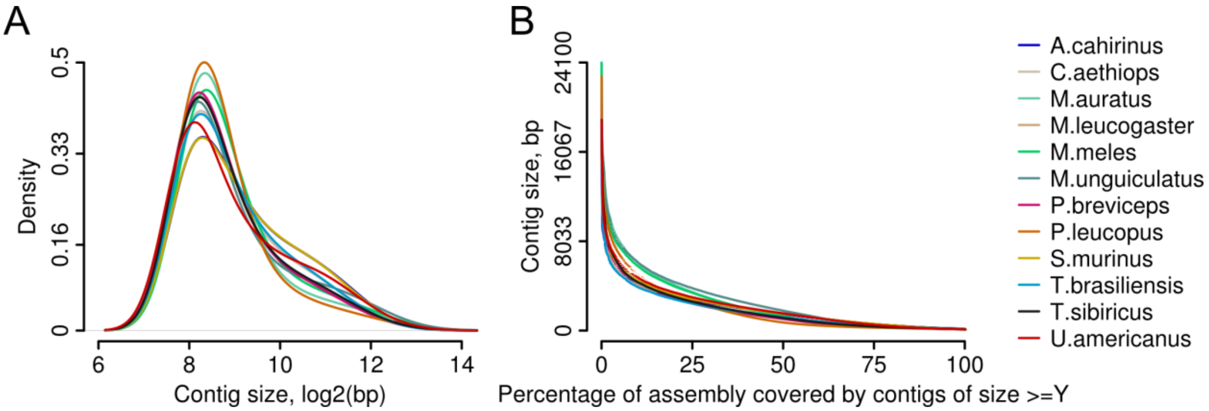

Figure S1. *De novo* assembled transcriptomic contigs. (A) Density of RNA-seq contig lengths. The vertical axis of a graph shows density, and the horizontal axis shows contig size (in nucleotide base pairs) in logarithmic space. (B) Contig size coverage. The vertical axis of a graph shows contig size (in nucleotide base pairs), and horizontal axis shows assembly coverage (percentage). Species are indicated in the right corner of plot.

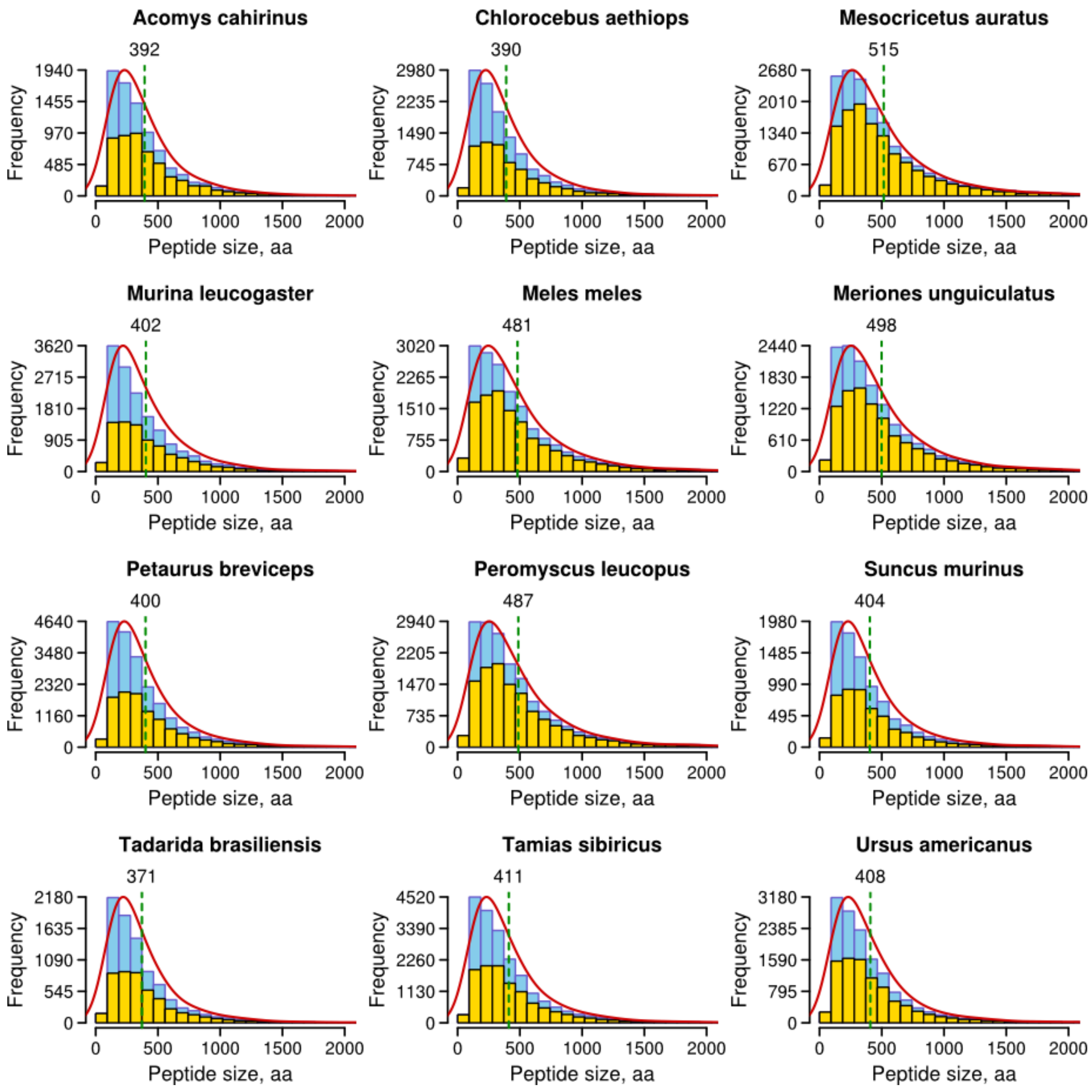

Figure S2. *Ab initio* predicted peptides. Each panel shows the distribution of peptide lengths for one of the indicated species (top). Blue bars denote peptides predicted from a complete set of coding sequences (CDSs). Yellow bars indicate peptides predicted from CDSs that have start and stop translation signals. Red curve is the kernel density estimate of CDS lengths. Green dashed lines with numbers at the top indicate median size of proteomes.

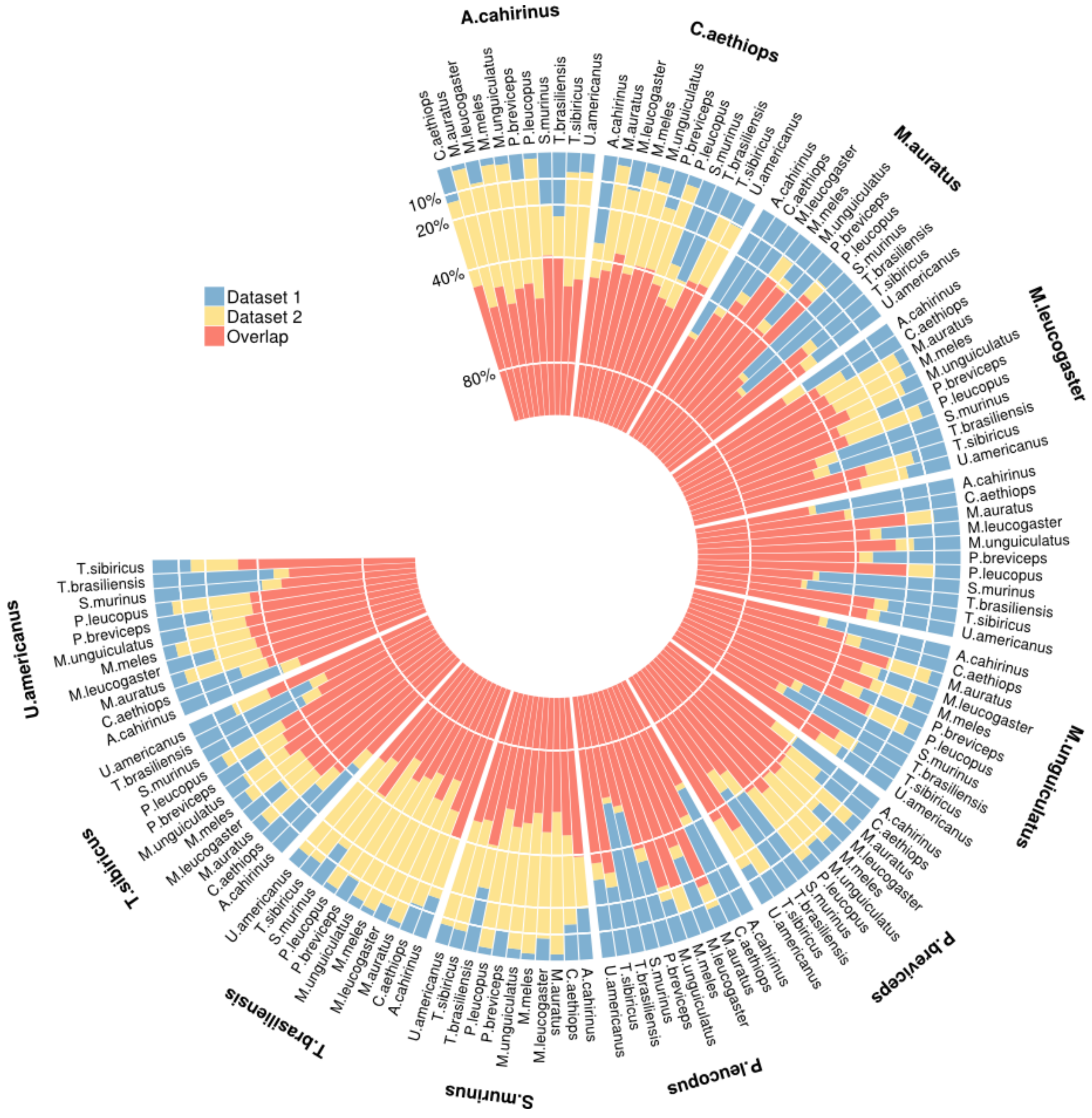

Figure S3. Orthologous relationships inferred from *ab initio* predicted peptides. Each color-coded bar shows percentages of peptides (scale at the beginning) unique for two species (blue, yellow) and percentage of orthologous peptides (red).

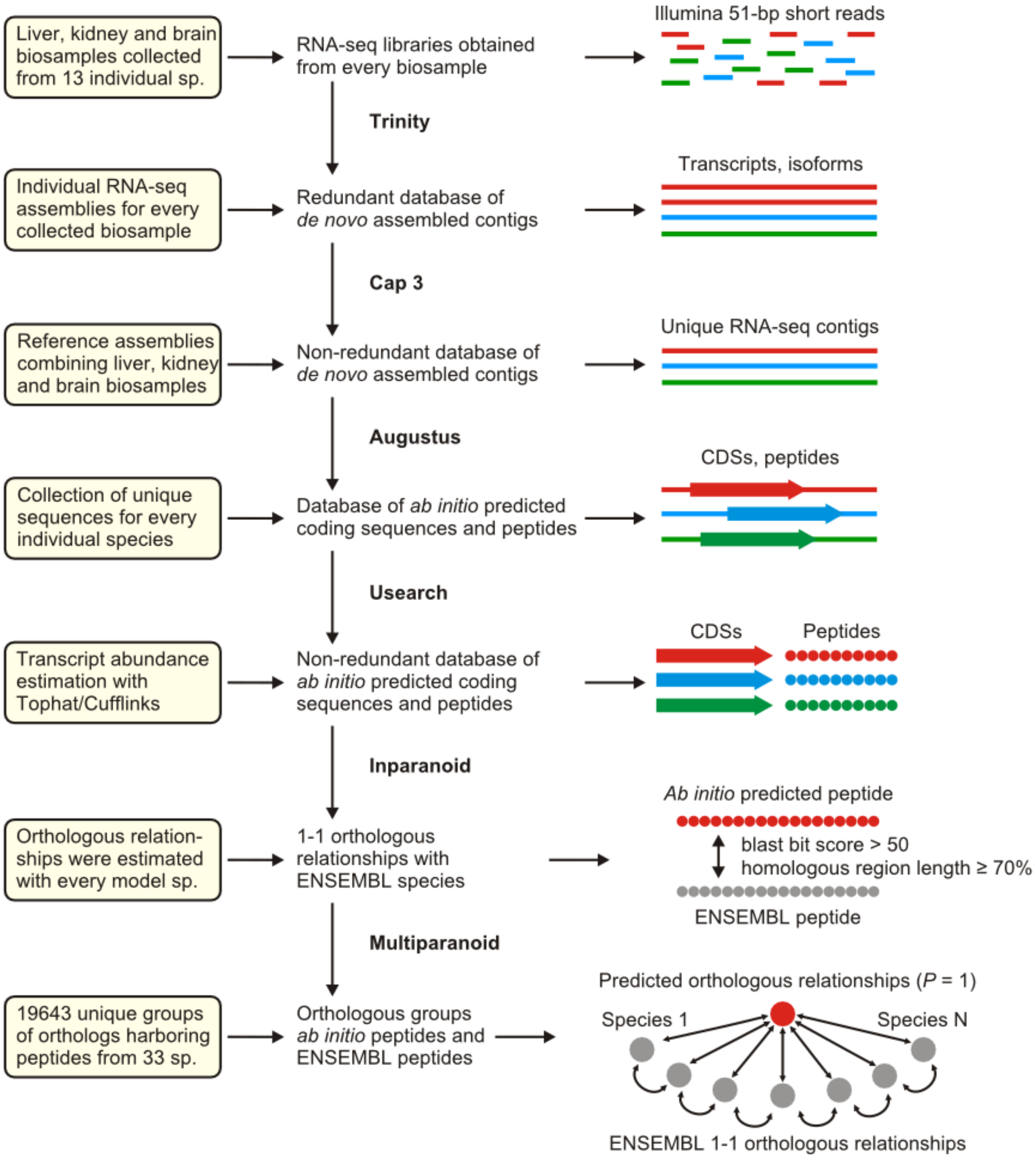

Figure S4. Schematic representation of methods used for *de novo* transcriptome assembly, annotation and inferring of orthologous relationships.

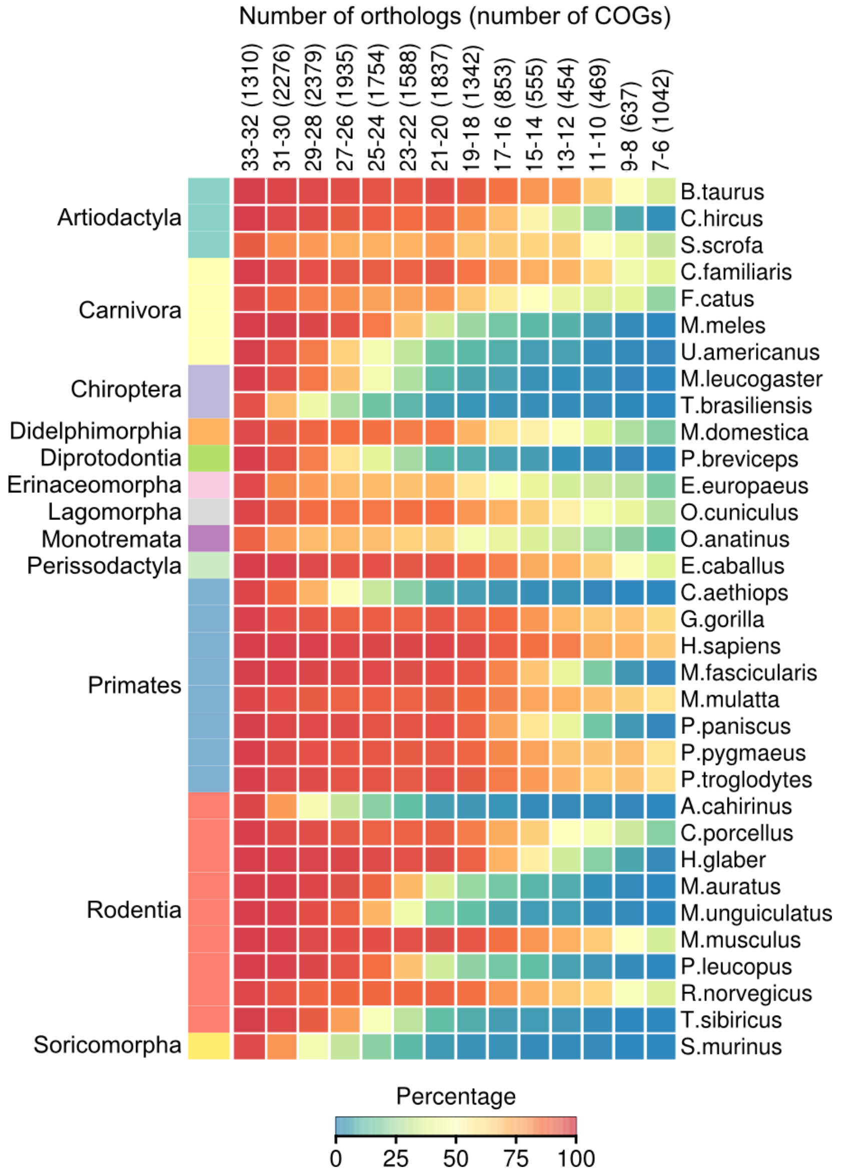

Figure S5. Cluster heat map that shows quantitative representation of orthologs in COG. Number of orthologs and total number of COG of particular size (in brackets) is indicated at the top of plot. Species are shown in the right corner of plot. Each colored cell denotes percentage of species-specific orthologs that belongs to COG of particular size (scale at the bottom).

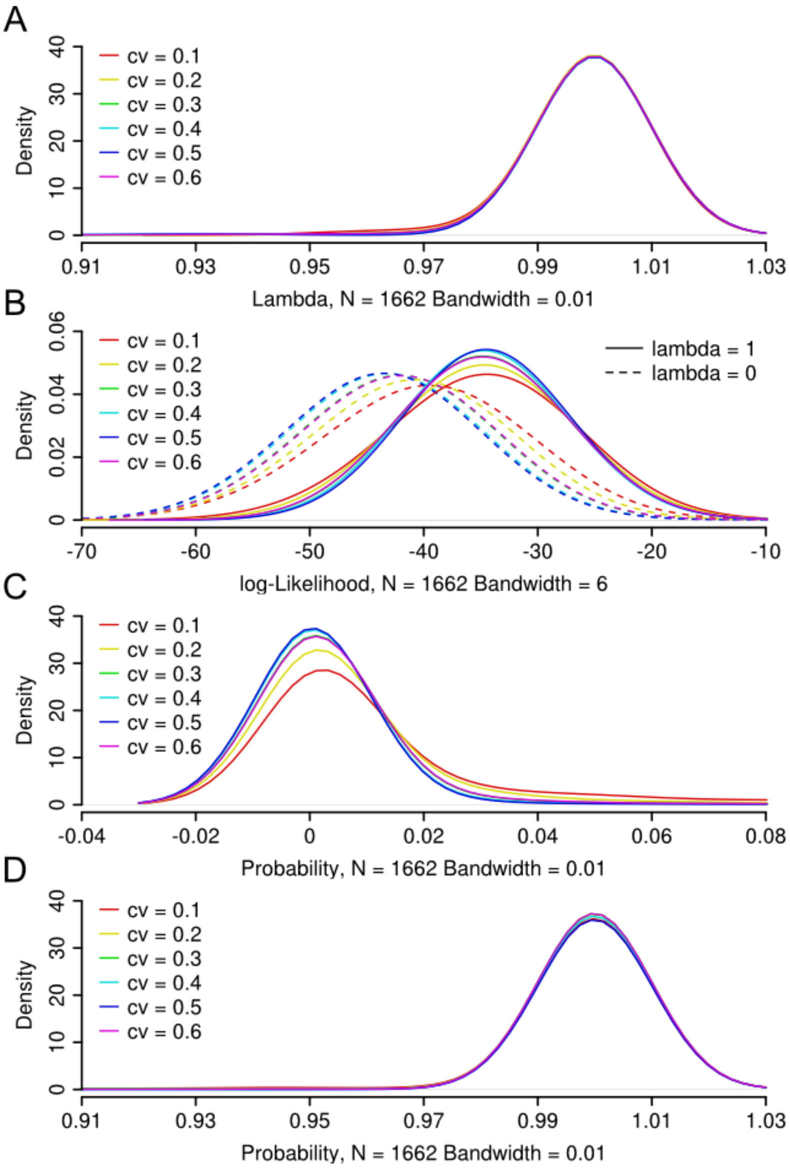

Figure S6. Influence of measurement error on the λ model. We simulated evolution of N = 1662 traits under the Brownian motion. We then added fixed measurement error to traits (*CV* varied from 0.1 to 0.6). (A) Kernel density estimate of λ coefficient. Color of lines denotes *CV* (legend in the top left corner of plot). (B) Kernel density estimate of log-likelihoods of the λ model. Color-coded dashed lines correspond to log-likelihoods of the model when trait variation was compared with randomly distributed value (star-like tree topology, model M0). Solid lines correspond to log-likelihoods of the model when trait variation was compared with the BM model (untransformed tree topology, model M1). (C) Kernel density estimate of *P*-values. Probabilities were obtained from the likelihood-ratio tests (LRT) between log-likelihoods of the λ model and log-likelihoods of the M0 model. (D) Kernel density estimate of *P*-values. Probabilities were obtained from the LRT between log-likelihoods of the λ model and log-likelihoods of the M1 model. Kernel density estimates were calculated using Gaussian approximation with smoothing bandwidth indicated below each panel.

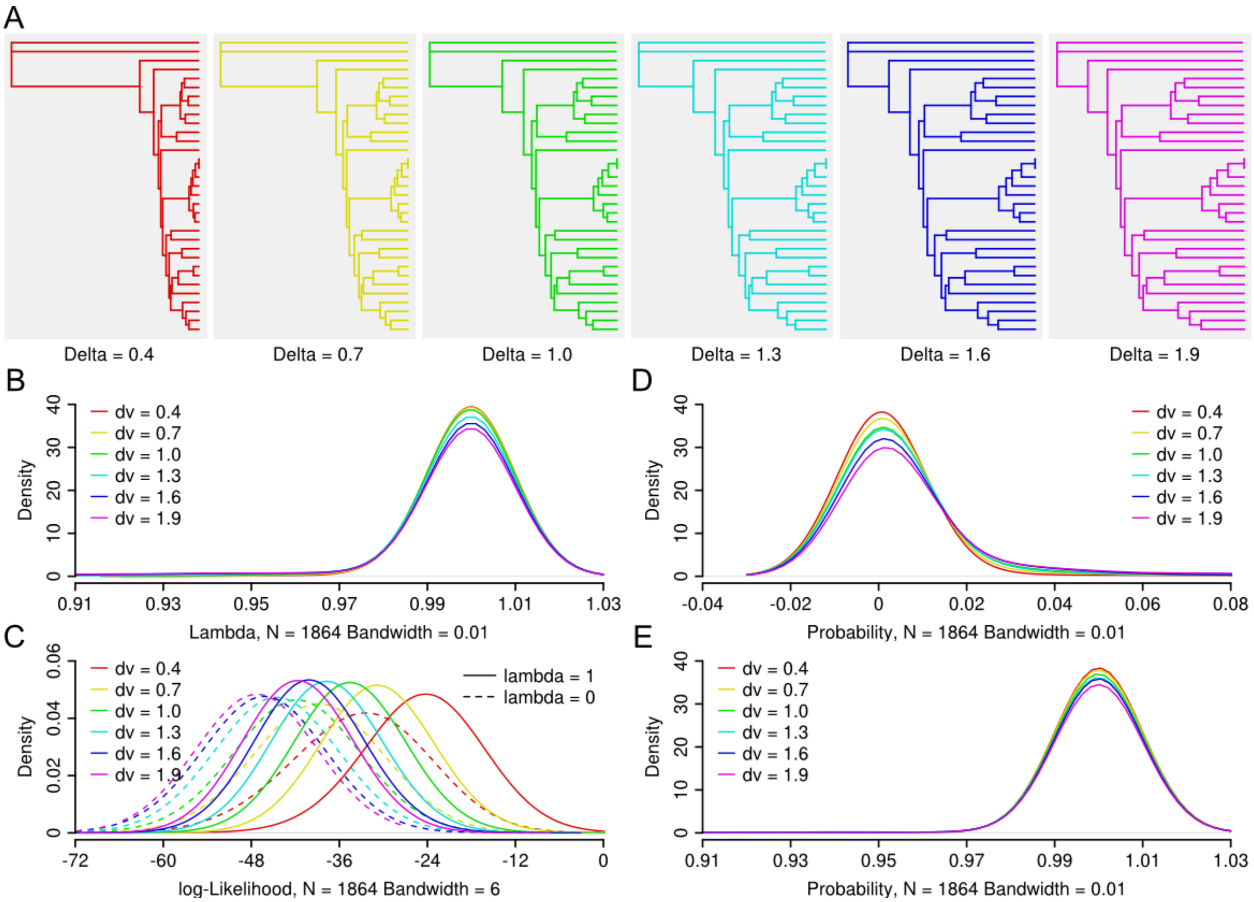

Figure S7. Influence of non-constant evolution rate on the λ model. (A) A set of trees created with δ branch length transformation approach. For every topology we simulated N = 1864 traits under the Brownian motion and tested the λ model against untransformed tree topology (δ = 1). (B) Kernel density estimate of λ coefficient. Color of lines denotes particular tree topology used for simulation of character evolution (legend in the top left corner of plot). (C) Kernel density estimate of log-likelihoods. Color-coded dashed lines correspond to log-likelihoods of the models when trait variation was compared with randomly distributed value (star-like tree topology, model M0). Solid lines correspond to log-likelihoods of the models when trait variation was compared with the BM model (untransformed topology, model M1). (D) Kernel density estimate of *P*-values. Probabilities were obtained from the likelihood-ratio tests (LRT) between log-likelihoods of the λ model and log-likelihoods of the M0 model. (E) Kernel density estimate of *P*-values. Probabilities were obtained from the LRT between log-likelihoods of the λ model and log-likelihoods of the M1 model. Kernel density estimates were calculated using Gaussian approximation with smoothing bandwidth indicated below each panel.

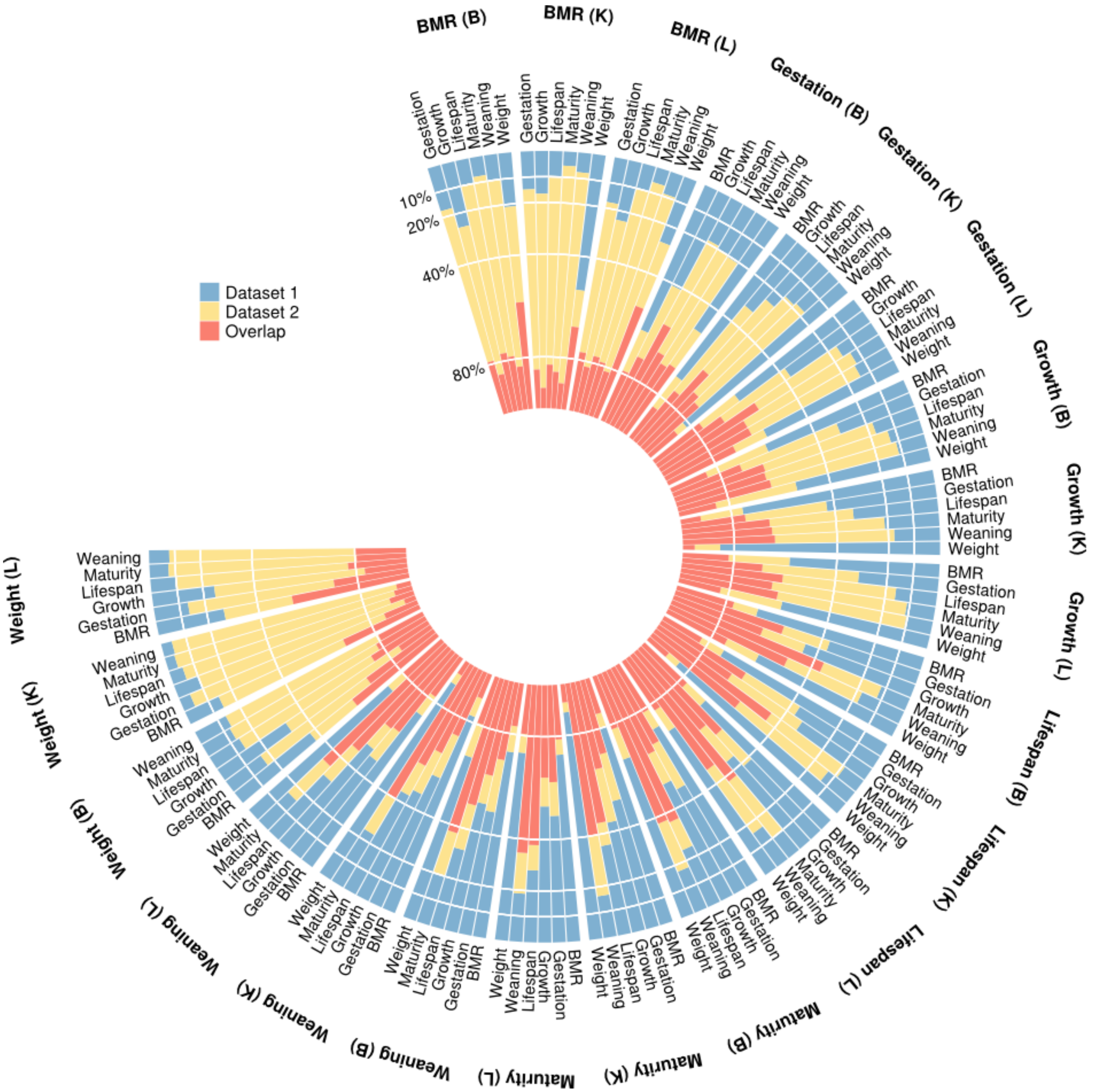

Figure S8. Overlap of genes whose expression variation associate with life histories. Each color-coded bar shows percentages of genes (scale at the beginning) unique for two data sets (blue, yellow) and percentage of common genes (red). L, liver; K, kidney; B, brain.

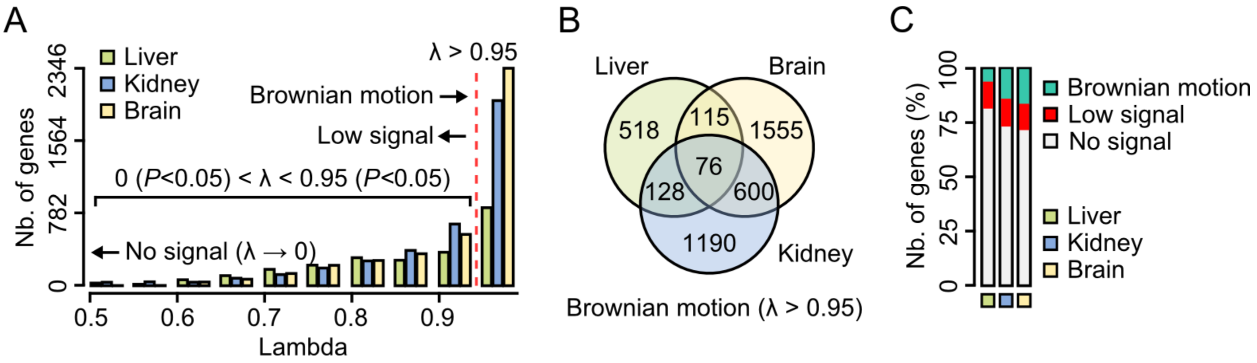

Figure S9. The diffusive model of transcriptome evolution. (A) Distributions of λ coefficients in the liver, kidney and brain. Each bar shows the numbers of genes (vertical axis) within a particular λ estimate (bottom axis). Red line denotes λ cut-off for genes whose expression variation evolved under the BM model. (B) Overlap of genes with λ > 0.95 among the liver, kidney and brain. (C) Proportion of transcripts whose expression variation is consistent with the BM model (green) or evolved with varying degree of independence from phylogeny (red, grey).

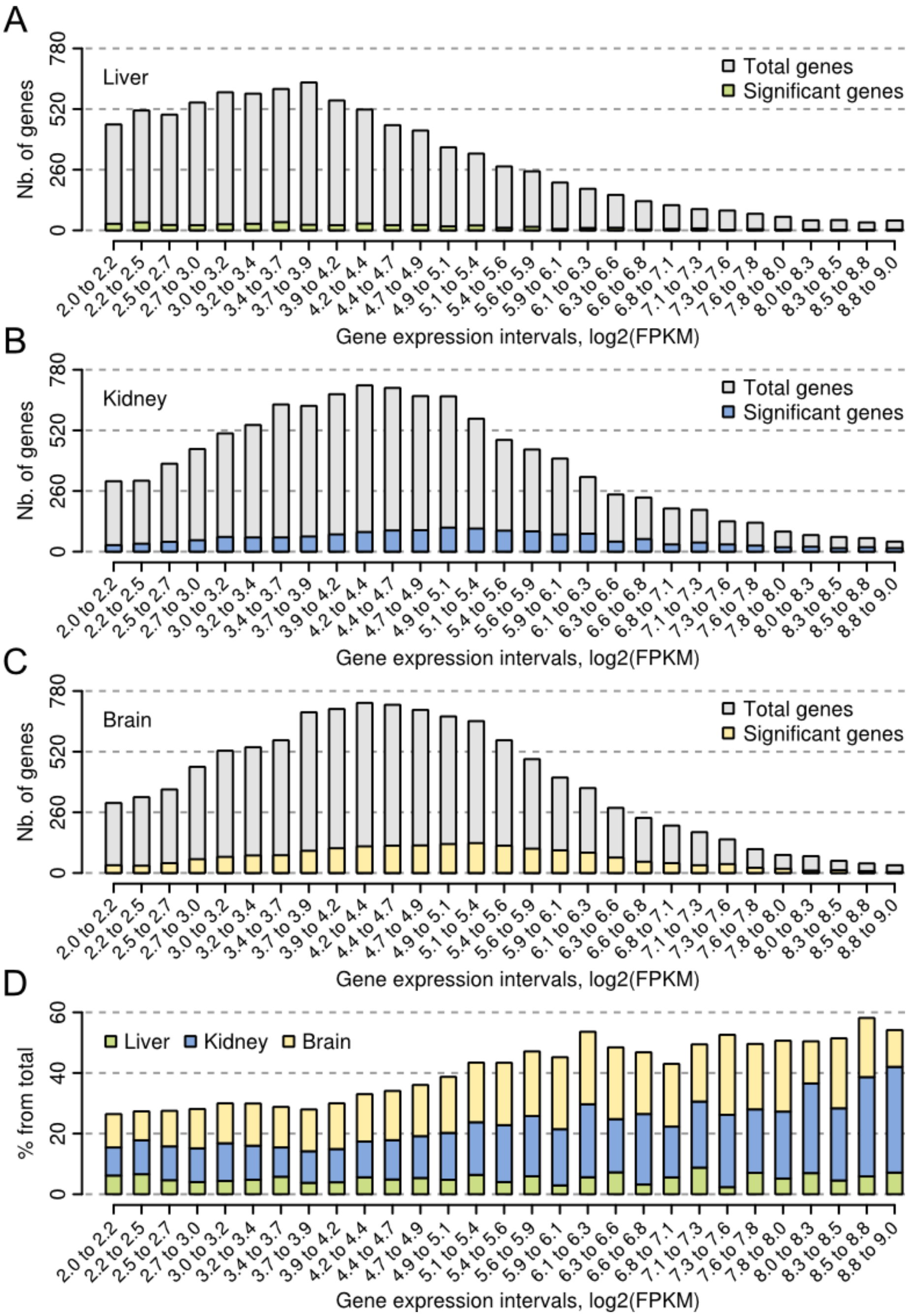

Figure S10. Distribution of transcripts with λ > 0.95 across gene expression levels. (A), (B) and (C) Histogram plots show distribution of transcript numbers across a range of gene expression levels in the liver, kidney and brain, respectively. Horizontal axes denote FPKM in logarithmic space. Vertical axes show transcript number. Grey bars show all transcripts. Transcripts with λ > 0.95 are highlighted with one of the three colors (legend in the top right corner). (D) Histogram shows distribution of transcripts with λ > 0.95 across a range of gene expression intervals. Horizontal axes denote FPKM in logarithmic space. Vertical axis shows percentage of transcripts with λ > 0.95 from total number of organ-specific transcripts within a particular FPKM interval.

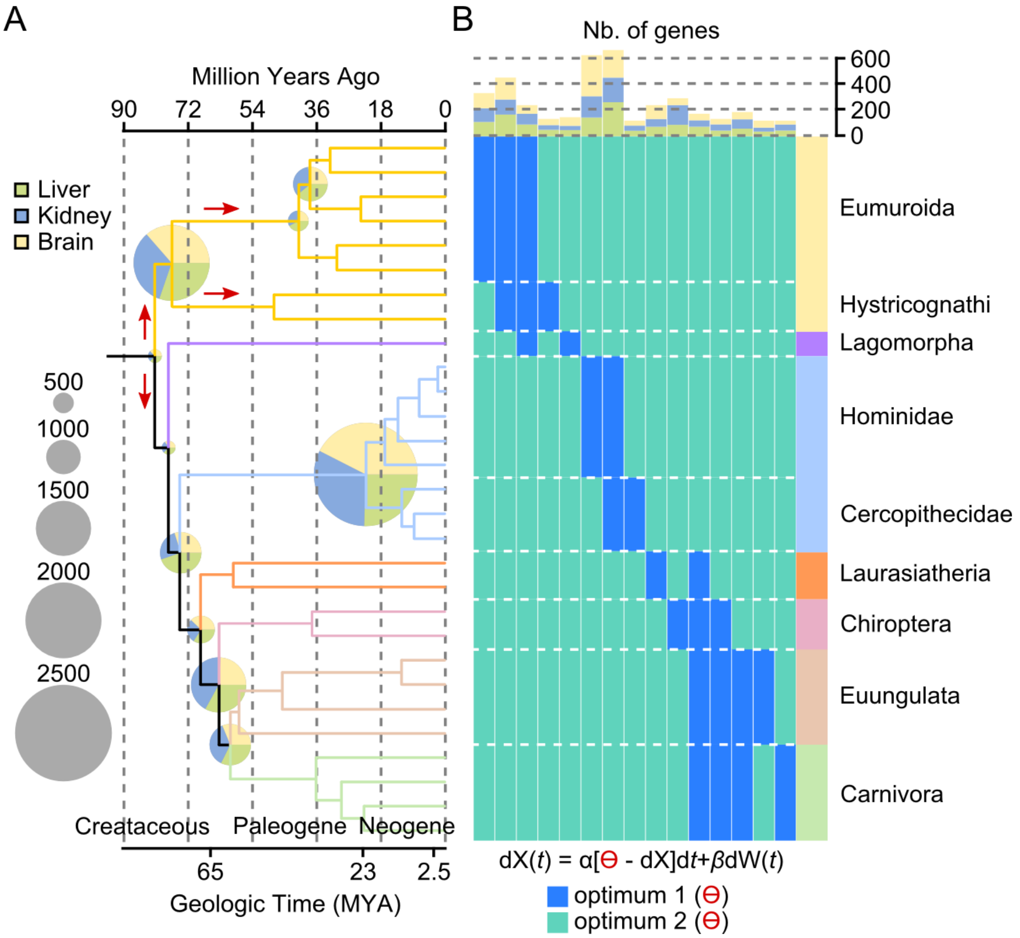

Figure S11. Macroevolution of gene expression modeled with the OU process. (A) Phylogeny and gene expression divergence of ancestors. Number of transcripts distinguishing child branches is shown in a pie chart with color-coded slices distinguishing organs. Grey circles in the left corner of plot denote number of transcripts corresponding to a particular pie size. (B) Patterns of gene expression variation inferred with the OU model. Colors distinguish OU optima. Numbers of genes corresponding to a particular OU regime are indicated at the top of each color-coded rectangle.

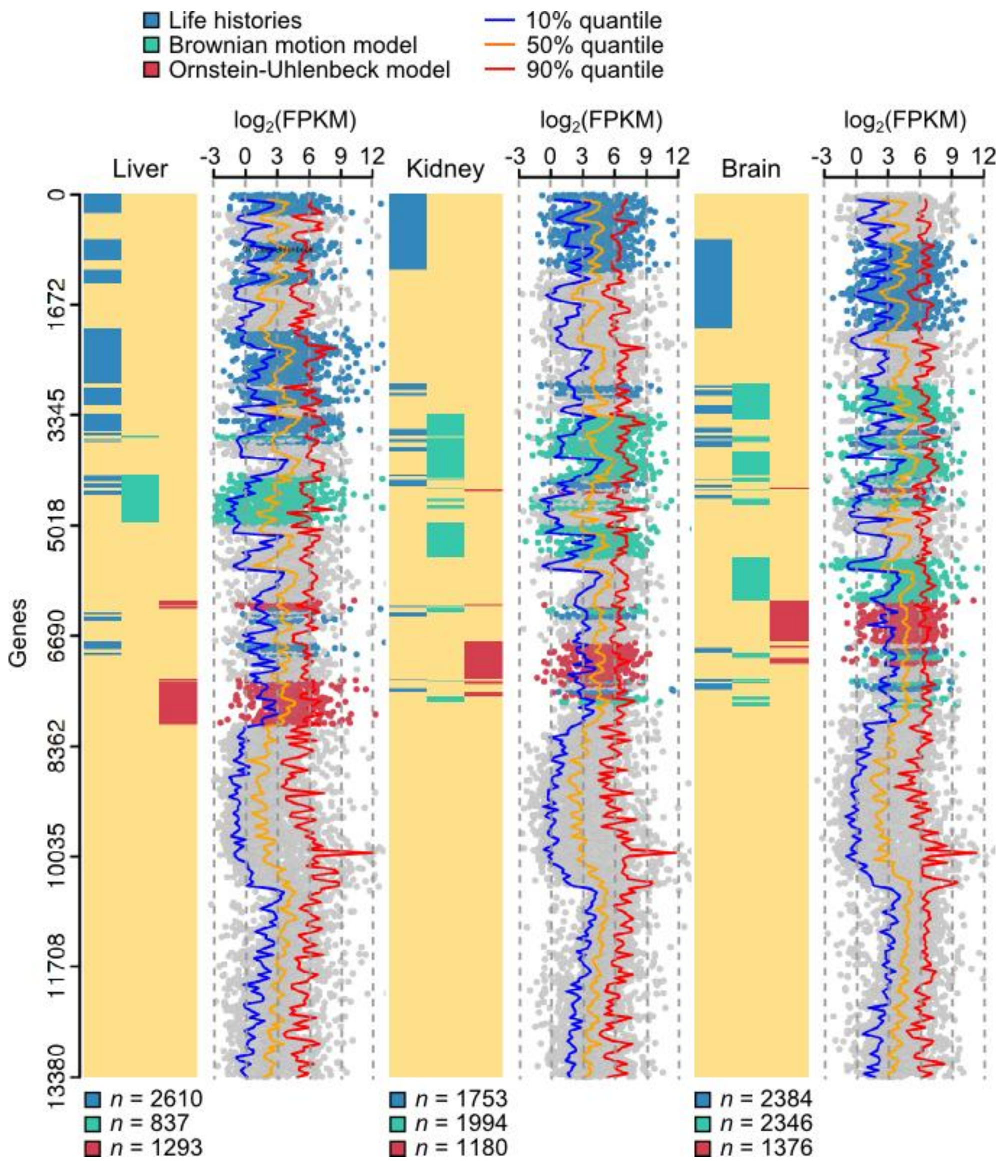

Figure S12. Summary on genes and models explaining interspecies expression variation. Color-coded cluster maps show a complete set of transcripts for one of the liver, kidney or brain. Colors distinguish genes whose expression variation could be explained by phylogeny (green), stabilizing constraints (red) or gradients of life history variation (blue). Total numbers of respective genes (*n*) is indicated at the bottom of each plot. Graphs on the left show organ-specific transcription levels averaged between all species. Color-coded lines denote 10%, 50% and 90% quantiles of expression variation calculated over 50-gene sliding window intervals.

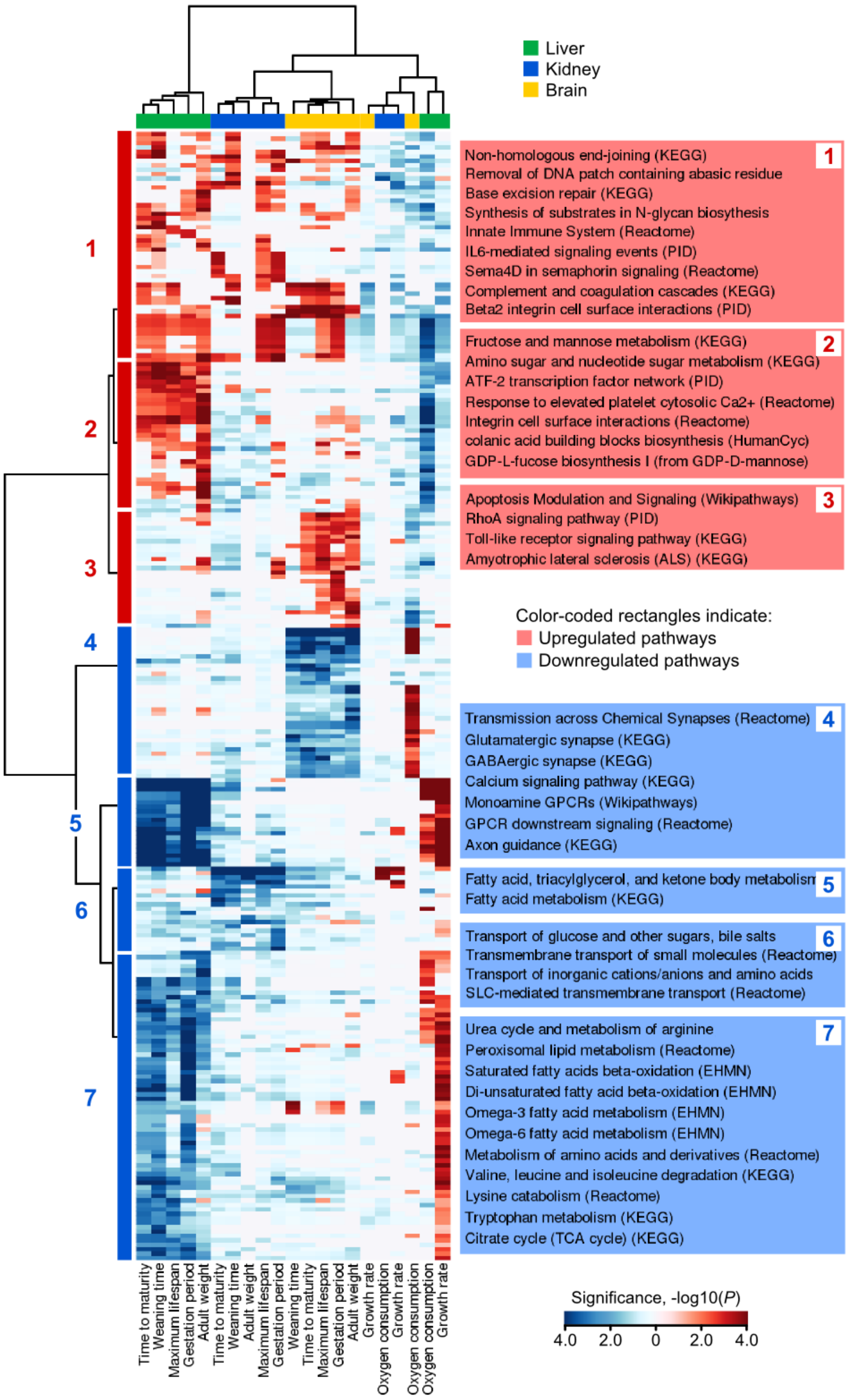
Liver
Kidney
Brain
1
Non-homologous end-joining (KEGG)
Removal of DNA patch containing abasic residue
Base excision repair (KEGG)
Synthesis of substrates in N-glycan biosythesis
Innate Immune System (Reactome)
IL6-mediated signaling events (PID)
Sema4D in semaphorin signaling (Reactome)
Complement and coagulation cascades (KEGG)
Beta2 integrin cell surface interactions (PID)
2
Fructose and mannose metabolism (KEGG)
Amino sugar and nucleotide sugar metabolism (KEGG)
ATF-2 transcription factor network (PID)
Response to elevated platelet cytosolic Ca2+ (Reactome)
Integrin cell surface interactions (Reactome)
colanic acid building blocks biosynthesis (HumanCyc)
GDP-L-fucose biosynthesis I (from GDP-D-mannose)
3
Apoptosis Modulation and Signaling (Wikipathways)
RhoA signaling pathway (PID)
Toll-like receptor signaling pathway (KEGG)
Amyotrophic lateral sclerosis (ALS) (KEGG)
Color-coded rectangles indicate:
Upregulated pathways
Downregulated pathways
4
Transmission across Chemical Synapses (Reactome)
Glutamatergic synapse (KEGG)
GABAergic synapse (KEGG)
Calcium signaling pathway (KEGG)
Monoamine GPCRs (Wikipathways)
GPCR downstream signaling (Reactome)
Axon guidance (KEGG)
5
Fatty acid, triacylglycerol, and ketone body metabolism
Fatty acid metabolism (KEGG)
6
Transport of glucose and other sugars, bile salts
Transmembrane transport of small molecules (Reactome)
Transport of inorganic cations/anions and amino acids
SLC-mediated transmembrane transport (Reactome)
7
Urea cycle and metabolism of arginine
Peroxisomal lipid metabolism (Reactome)
Saturated fatty acids beta-oxidation (EHMN)
Di-unsaturated fatty acid beta-oxidation (EHMN)
Omega-3 fatty acid metabolism (EHMN)
Omega-6 fatty acid metabolism (EHMN)
Metabolism of amino acids and derivatives (Reactome)
Valine, leucine and isoleucine degradation (KEGG)
Lysine catabolism (Reactome)
Tryptophan metabolism (KEGG)
Citrate cycle (TCA cycle) (KEGG)
1
2
3
4
5
6
7
Time to maturity
Weaning time
Maximum lifespan
Gestation period
Adult weight
Time to maturity
Weaning time
Adult weight
Maximum lifespan
Gestation period
Weaning time
Time to maturity
Maximum lifespan
Gestation period
Adult weight
Growth rate
Oxygen consumption
Growth rate
Oxygen consumption
Oxygen consumption
Growth rate
Significance, -log10(P)
4.0
2.0
0
2.0
4.0

Figure S13. A cluster map that shows biological pathways associated with life histories. Columns on the plot correspond to life histories (bottom). Rows show pathways. Sub rectangles in red denote pathways positively correlated with life history variable. Negatively correlated pathways are in blue. Color intensities denote statistical significance of enrichment (negative logarithm of FDR-corrected *P*-value, bar in the bottom right corner of plot). Life histories and pathways were clustered using the Ward's method and Euclidean distance metric. Pathways were grouped into 7 clusters using constant height cutoff method (left side). Titles of representative pathways and database source (in brackets) are presented in the right corner of plot.

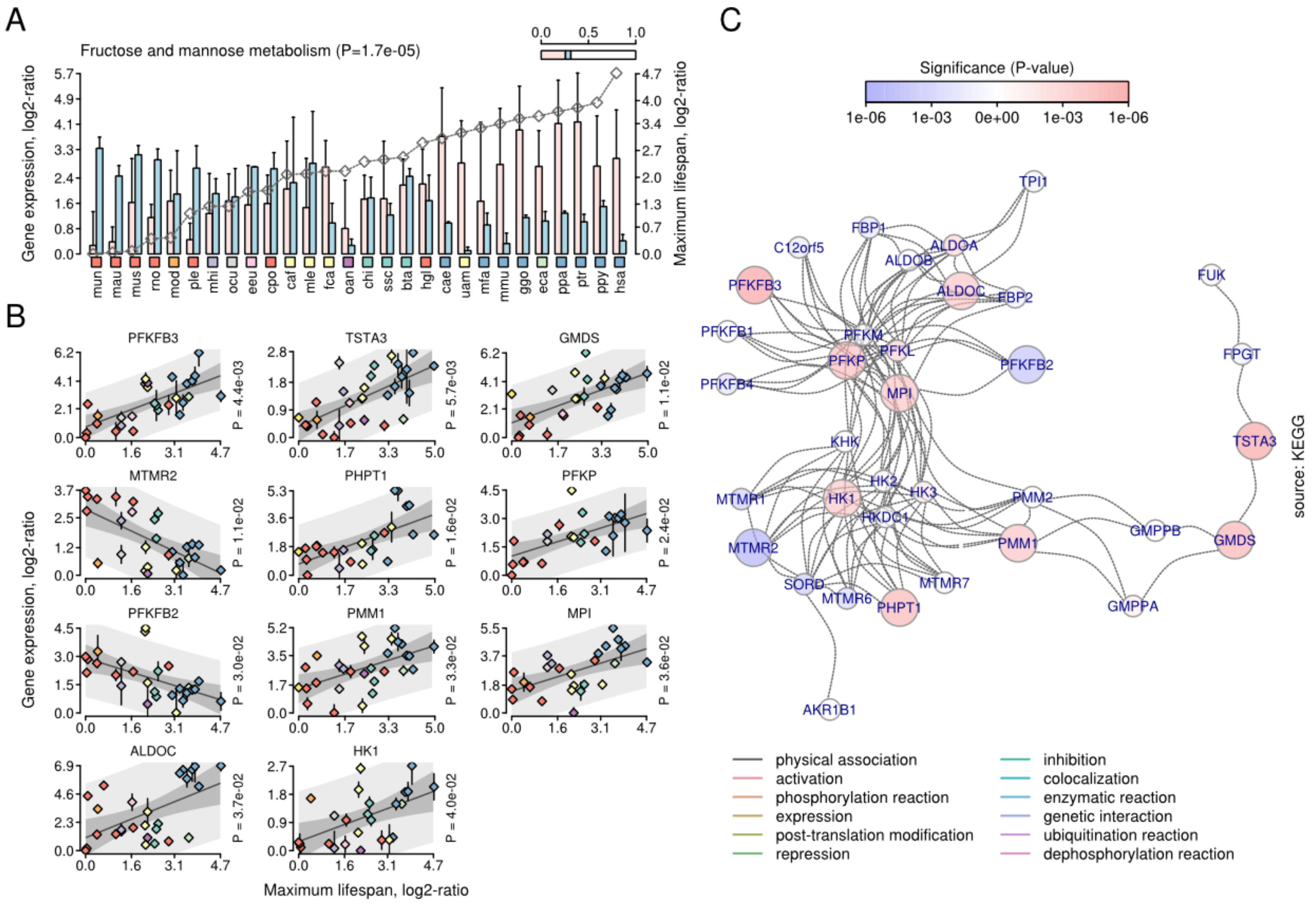

Figure S14. Genes encoding enzymes of carbohydrate degradation pathway are differentially expressed in liver. (A) Mean FPKM of all significant genes. Bars in pink denote upregulated genes. Blue bars correspond to downregulated genes. Error bars indicate standard deviation of the mean. Grey line is the relative value of life history variable (maximum lifespan, axis on the right). Species are shown at the bottom. Color-coded rectangles distinguish

lineages. Bar at the top right shows proportion of significant genes from all genes associated with this pathway. *P*-value denotes statistical enrichment (right-sided hypergeometric test). (B) Genes whose expression variation correlates with life history variation. Vertical axis is the relative FPKM log2-transformed. Horizontal axis is the relative life history variable in logarithmic space. Rhombs are the means of FPKM. Colors of rhombs distinguish lineages. Error bars show standard deviation of the mean. *P*-value denotes significance of the OLS model. Median grey line is best-fit OLS line. Shaded areas indicate observed and predicted upper (95%) and lower (5%) confidence intervals. (C) Functional interaction network. Color of nodes denotes significance of the OLS model. Positively correlated genes are in red. Negatively correlated genes are in blue. Color of edges denotes type of interaction (bottom).

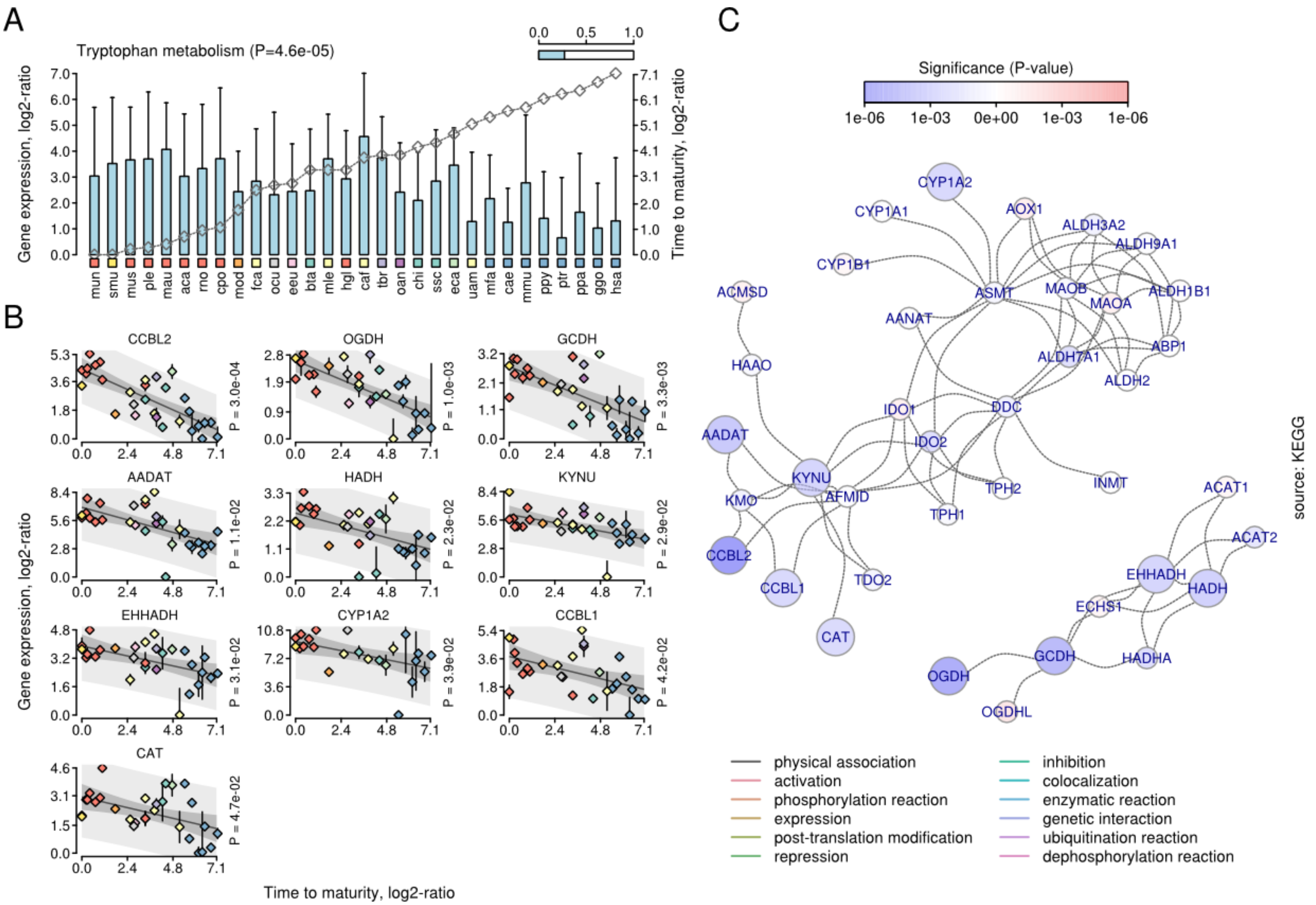

Figure S15. Gene expression variation associated with tryptophan metabolism in liver. (A) Mean FPKM of all significant genes. Error bars indicate standard deviation of the mean. Grey line is the relative value of life history variable (time to maturity, axis on the right). Species are shown at the bottom. Color-coded rectangles distinguish lineages. Bar at the top right shows proportion of significant genes from all genes associated with this pathway. *P-*

value denotes statistical enrichment (right-sided hypergeometric test). (B) Genes whose expression variation correlates with life history variation. Vertical axis is the relative FPKM log2-transformed. Horizontal axis is the relative life history variable in logarithmic space. Rhombs are the means of FPKM. Colors of rhombs distinguish lineages. Error bars show standard deviation of the mean. *P*-value denotes significance of the OLS model. Median grey line is best-fit OLS line. Shaded areas indicate observed and predicted upper (95%) and lower (5%) confidence intervals. (C) Functional interaction network. Color of nodes denotes significance of the OLS model. Positively correlated genes are in red. Negatively correlated genes are in blue. Color of edges denotes type of interaction (bottom).

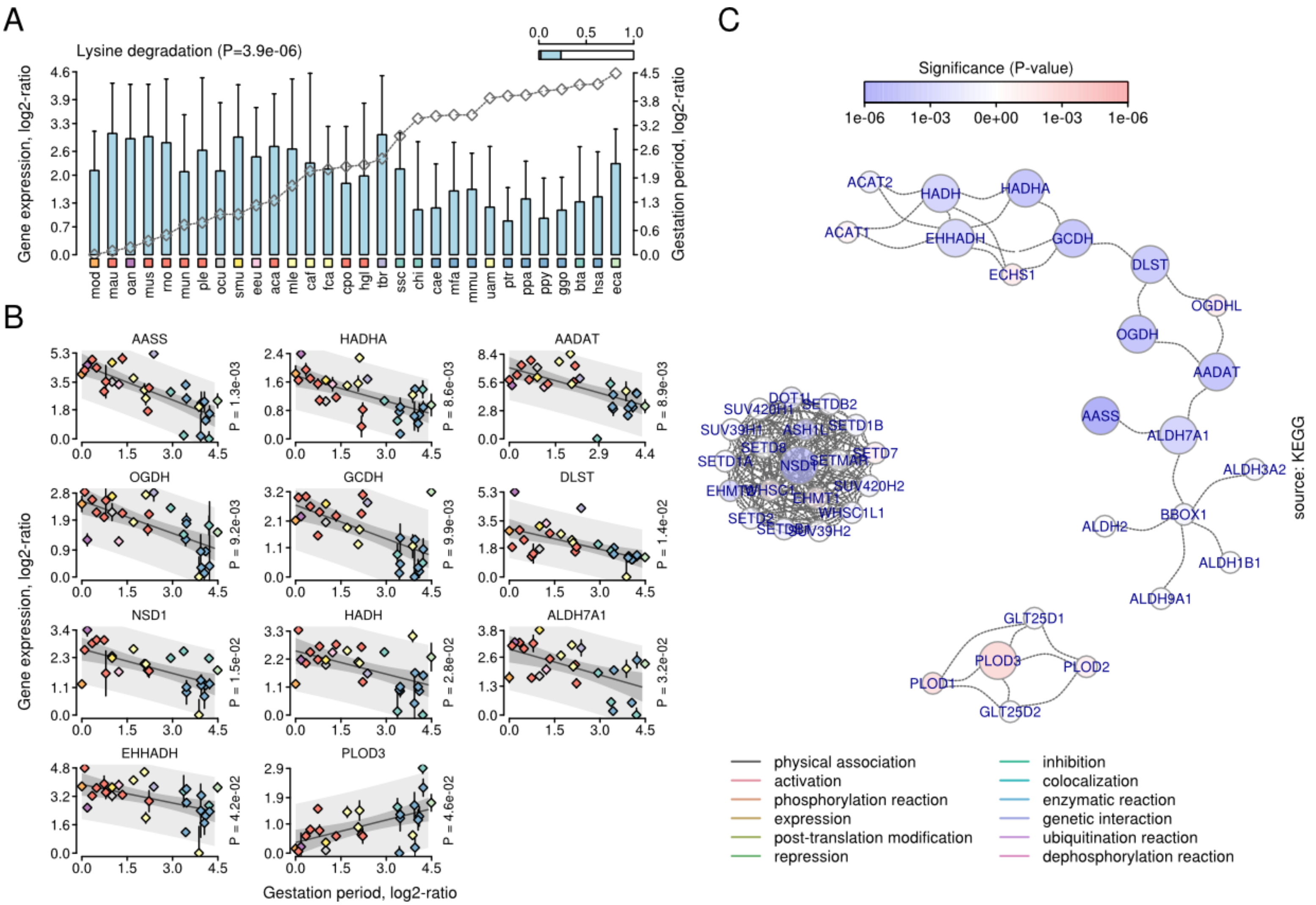

Figure S16. Gene expression variation associated with lysine metabolism in liver. (A) Mean FPKM of all significant genes. Error bars indicate standard deviation of the mean. Grey line is the relative value of life history variable (gestation period, axis on the right). Species are shown at the bottom. Color-coded rectangles distinguish lineages. Bar at the top right shows proportion of significant genes from all genes associated with this

pathway. *P*-value denotes statistical enrichment (right-sided hypergeometric test). (B) Genes whose expression variation correlates with life history variation. Vertical axis is the relative FPKM log2-transformed. Horizontal axis is the relative life history variable in logarithmic space. Rhombs are the means of FPKM. Colors of rhombs distinguish lineages. Error bars show standard deviation of the mean. *P*-value denotes significance of the OLS model. Median grey line is best-fit OLS line. Shaded areas indicate observed and predicted upper (95%) and lower (5%) confidence intervals. (C) Functional interaction network. Color of nodes denotes significance of the OLS model. Positively correlated genes are in red. Negatively correlated genes are in blue. Color of edges denotes type of interaction (bottom).

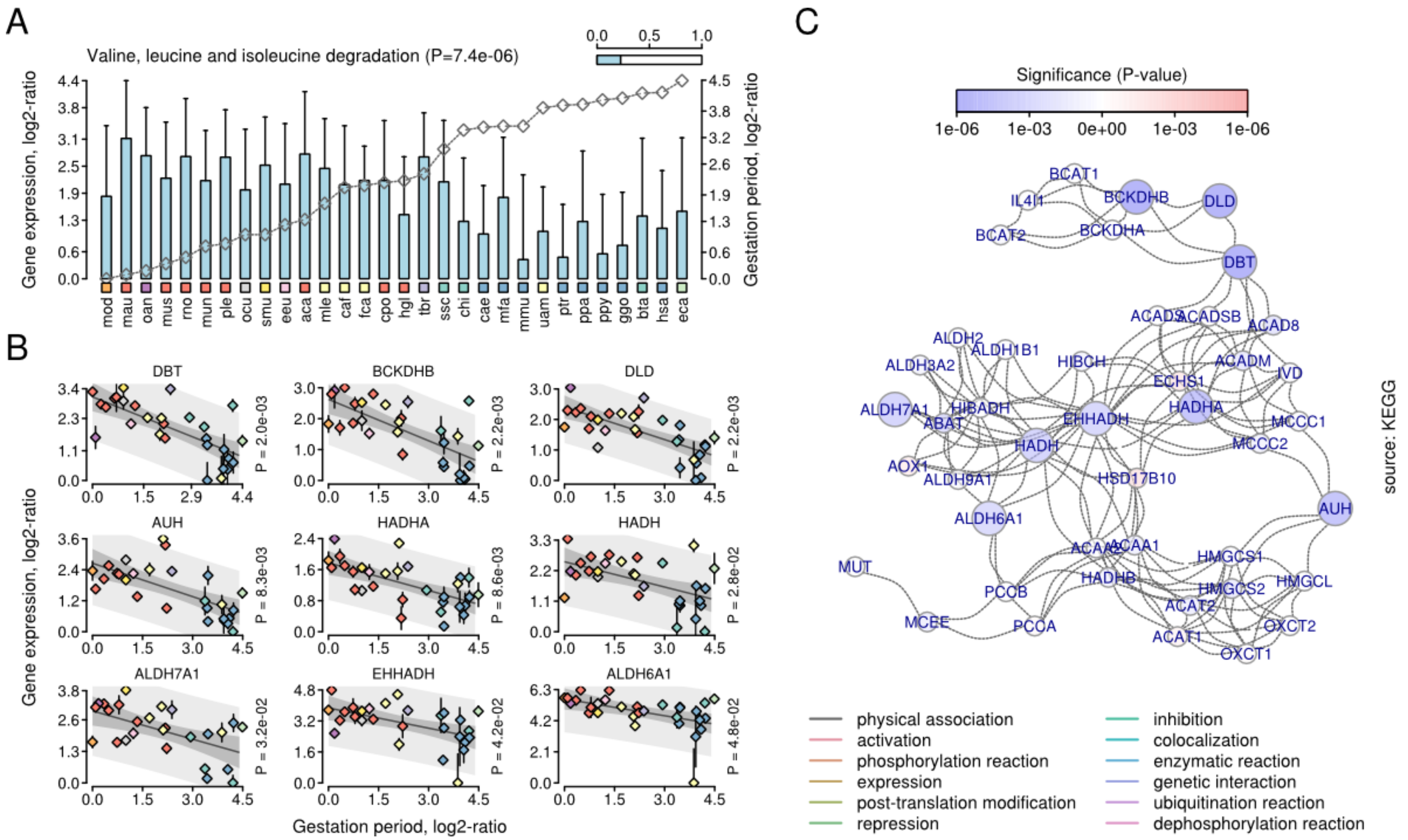

Figure S17. Gene expression variation associated with valine metabolism in liver. (A) Mean FPKM of all significant genes. Error bars indicate standard deviation of the mean. Grey line is the relative value of life history variable (gestation period, axis on the right). Species are shown at the bottom. Color-coded rectangles distinguish lineages. Bar at the top right shows proportion of significant genes from all genes associated with this pathway. *P*-value denotes statistical enrichment (right-sided hypergeometric test). (B) Genes whose expression variation correlates with life history variation. Vertical axis is the relative FPKM log2-transformed. Horizontal axis is the relative life history variable in logarithmic space. Rhombs are the means of FPKM. Colors of rhombs distinguish lineages. Error bars show standard deviation of the mean. *P*-value denotes significance of the OLS model.

Median grey line is best-fit OLS line. Shaded areas indicate observed and predicted upper (95%) and lower (5%) confidence intervals. (C) Functional interaction network. Color of nodes denotes significance of the OLS model. Positively correlated genes are in red. Negatively correlated genes are in blue. Color of edges denotes type of interaction (bottom).

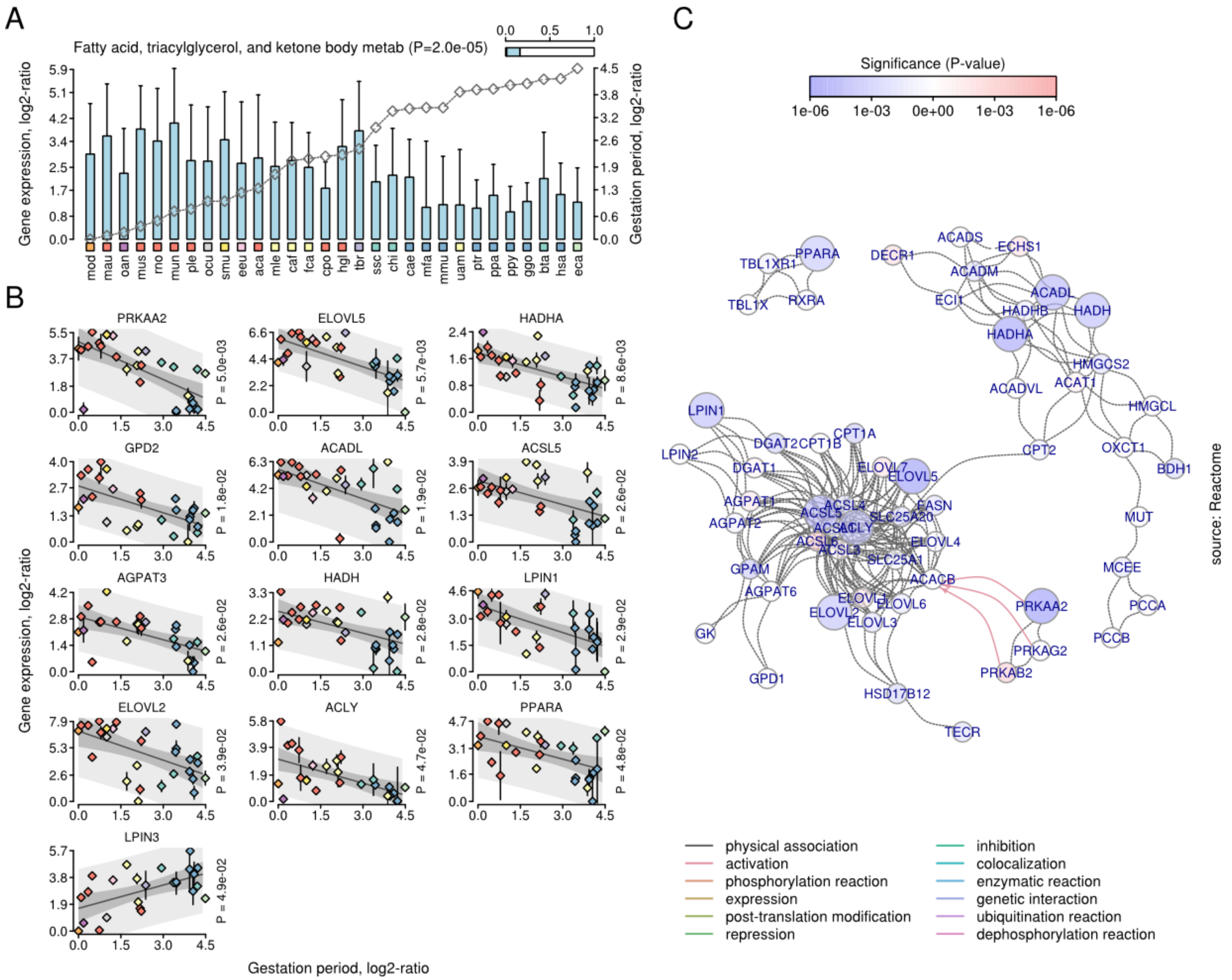
A
Fatty acid, triacylglycerol, and ketone body metab (P=2.0e-05)
Gene expression, log2-ratio
Gestation period, log2-ratio
mod
mau
oan
mus
rno
mun
ple
ocu
smu
eeu
aca
mle
caf
fca
cpo
hgl
tbr
ssc
chi
cae
mfa
mmu
uam
ptr
ppa
ppy
ggo
bta
hsa
eca
B
PRKAA2
P = 5.0e-03
ELOVL5
P = 5.7e-03
HADHA
P = 8.6e-03
GPD2
P = 1.8e-02
ACADL
P = 1.9e-02
ACSL5
P = 2.6e-02
AGPAT3
P = 2.6e-02
HADH
P = 2.8e-02
LPIN1
P = 2.9e-02
ELOVL2
P = 3.9e-02
ACLY
P = 4.7e-02
PPARA
P = 4.8e-02
LPIN3
P = 4.9e-02
Gene expression, log2-ratio
Gestation period, log2-ratio
C
Significance (P-value)
1e-06
1e-03
0e+00
1e-03
1e-06
source: Reactome
physical association
activation
phosphorylation reaction
expression
post-translation modification
repression
inhibition
colocalization
enzymatic reaction
genetic interaction
ubiquitination reaction
dephosphorylation reaction

Figure S18. Gene expression variation associated with fatty acid metabolism in liver. (A) Mean FPKM of all significant genes. Error bars indicate standard deviation of the mean. Grey line is the relative value of life history variable (gestation period, axis on the right). Species are shown at the bottom. Color-coded rectangles distinguish lineages. Bar at the top right shows proportion of significant genes from all genes associated with this pathway. *P*-value denotes statistical enrichment (right-sided hypergeometric test). (B) Genes whose expression variation correlates with life history variation. Vertical axis is the relative FPKM log2-transformed. Horizontal axis is the relative life history variable in logarithmic space. Rhombs are the means of FPKM. Colors of rhombs distinguish lineages. Error bars show standard deviation of the mean. *P*-value denotes significance of the OLS model. Median grey line is best-fit OLS line. Shaded areas indicate observed and predicted upper (95%) and lower (5%) confidence intervals. (C) Functional interaction network. Color of nodes denotes significance of the OLS model. Positively correlated genes are in red. Negatively correlated genes are in blue. Color of edges denotes type of interaction (bottom).

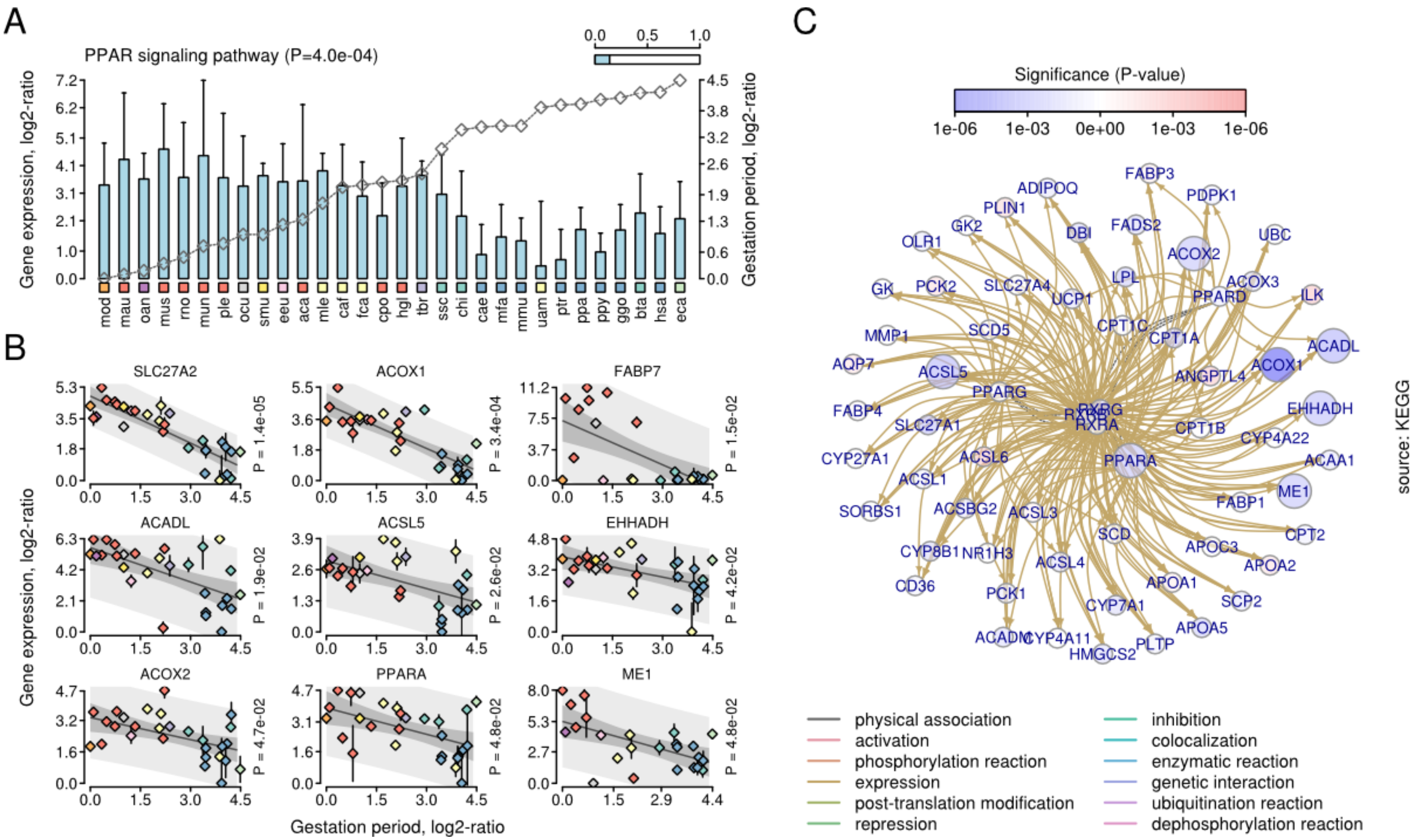

Figure S19. Gene expression variation associated with the peroxisome proliferator pathway in liver. (A) Mean FPKM of all significant genes. Error bars indicate standard deviation of the mean. Grey line is the relative value of life history variable (gestation period, axis on the right). Species are shown at the bottom. Color-coded rectangles distinguish lineages. Bar at the top right shows proportion of significant genes from all genes associated with this pathway. *P*-value denotes statistical enrichment (right-sided hypergeometric test). (B) Genes whose expression variation correlates with life history variation. Vertical axis is the relative FPKM log2-transformed. Horizontal axis is the relative life history variable in logarithmic space. Rhombs are the means of FPKM. Colors of rhombs distinguish lineages. Error bars show standard deviation of the mean. *P*-value denotes significance of the OLS model.

Median grey line is best-fit OLS line. Shaded areas indicate observed and predicted upper (95%) and lower (5%) confidence intervals. (C) Functional interaction network. Color of nodes denotes significance of the OLS model. Positively correlated genes are in red. Negatively correlated genes are in blue. Color of edges denotes type of interaction (bottom).

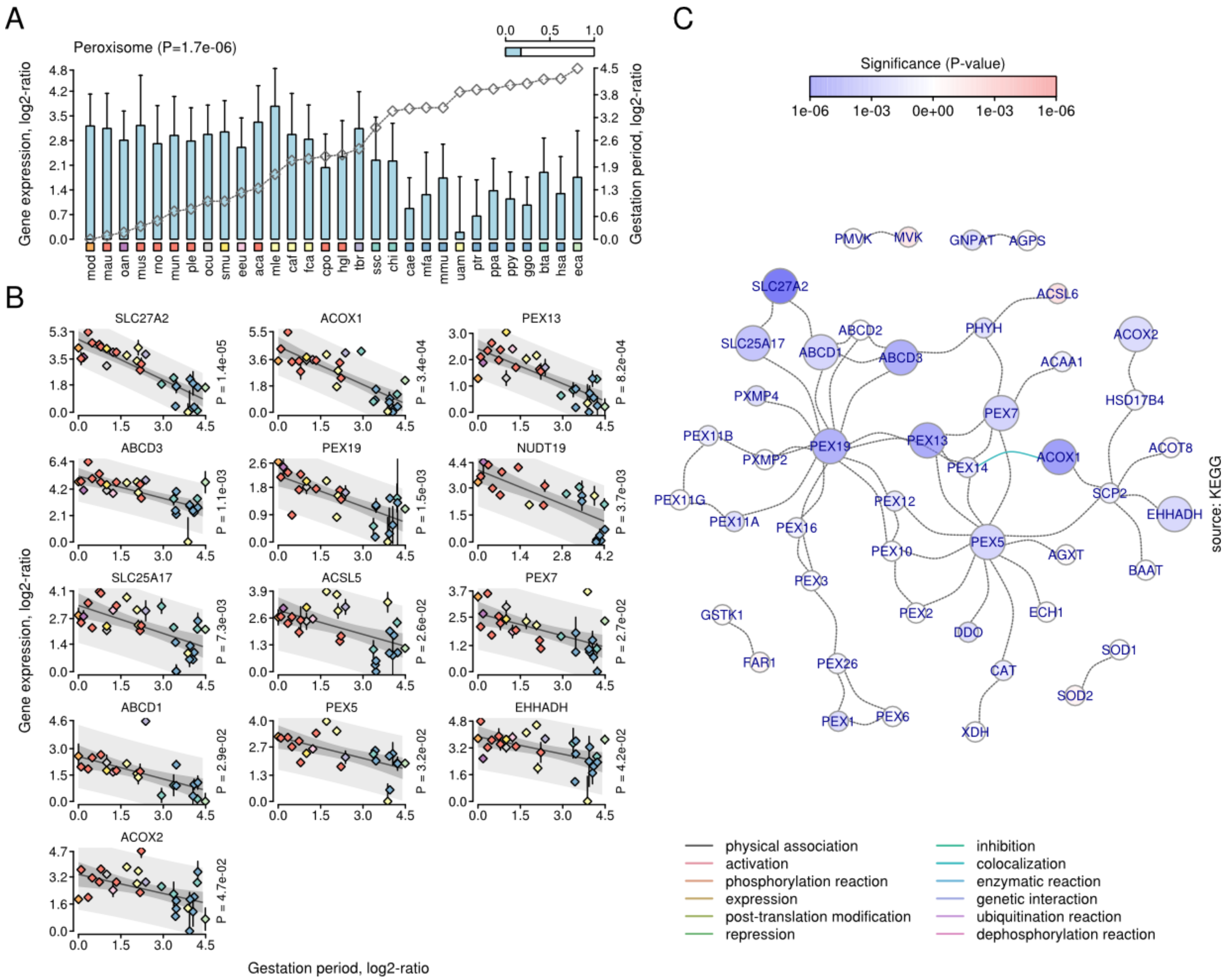
A
Peroxisome (P=1.7e-06)
Gene expression, log2-ratio
Gestation period, log2-ratio
mod mau oan mus rno mun ple ocu smu eeu aca mle caf fca cpo hgl tbr ssc chi cae mfa mmu uam ptr ppa ppy ggo bta hsa eca
B
SLC27A2
ACOX1
PEX13
ABCD3
PEX19
NUDT19
SLC25A17
ACSL5
PEX7
ABCD1
PEX5
EHHADH
ACOX2
Gene expression, log2-ratio
Gestation period, log2-ratio
P = 1.4e-05
P = 3.4e-04
P = 8.2e-04
P = 1.1e-03
P = 1.5e-03
P = 3.7e-03
P = 7.3e-03
P = 2.6e-02
P = 2.7e-02
P = 2.9e-02
P = 3.2e-02
P = 4.2e-02
P = 4.7e-02
C
Significance (P-value)
1e-06 1e-03 0e+00 1e-03 1e-06
source: KEGG
physical association
activation
phosphorylation reaction
expression
post-translation modification
repression
inhibition
colocalization
enzymatic reaction
genetic interaction
ubiquitination reaction
dephosphorylation reaction

Figure S20. Gene expression variation associated with peroxisome negatively correlates with life history variation in liver. (A) Mean FPKM of all significant genes. Error bars indicate standard deviation of the mean. Grey line is the relative value of life history variable (gestation period, axis on the right). Species are shown at the bottom. Color-coded rectangles distinguish lineages. Bar at the top right shows proportion of significant genes from all genes associated with this pathway. *P*-value denotes statistical enrichment (right-sided hypergeometric test). (B) Genes whose expression variation correlates with life history variation. Vertical axis is the relative FPKM log2-transformed. Horizontal axis is the relative life history variable in logarithmic space. Rhombs are the means of FPKM. Colors of rhombs distinguish lineages. Error bars show standard deviation of the mean. *P*-value denotes significance of the OLS model. Median grey line is best-fit OLS line. Shaded areas indicate observed and predicted upper (95%) and lower (5%) confidence intervals. (C) Functional interaction network. Color of nodes denotes significance of the OLS model. Positively correlated genes are in red. Negatively correlated genes are in blue. Color of edges denotes type of interaction (bottom).

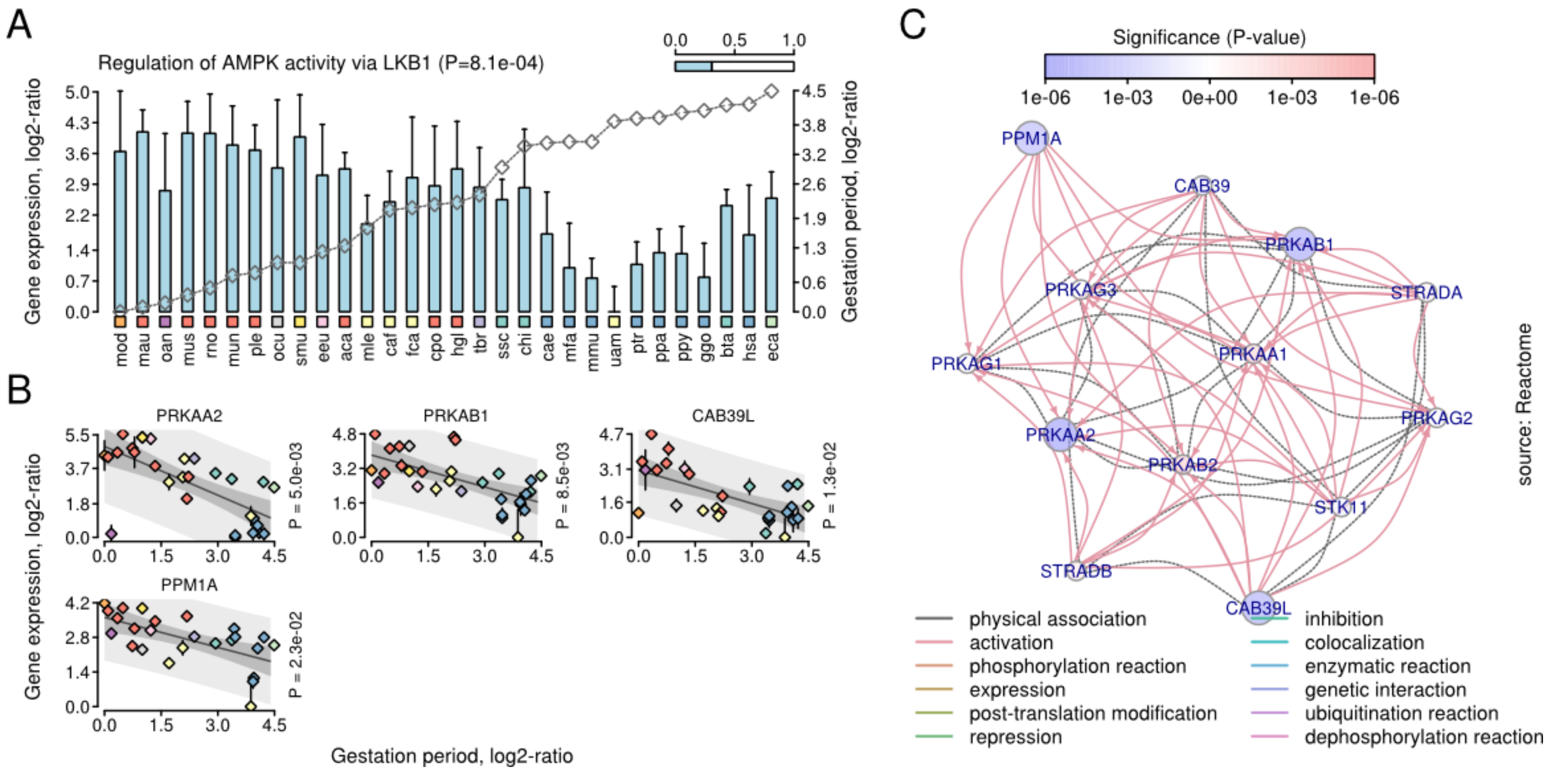

Figure S21. Gene expression variation associated with AMPK signaling negatively correlates with life history variation in liver. (A) Mean FPKM of all significant genes. Error bars indicate standard deviation of the mean. Grey line is the relative value of life history variable (gestation period, axis on the right). Species are shown at the bottom. Color-coded rectangles distinguish lineages. Bar at the top right shows proportion of significant genes from all genes associated with this pathway. *P*-value denotes statistical enrichment (right-sided hypergeometric test). (B) Genes whose expression variation correlates with life history variation. Vertical axis is the relative FPKM log2-transformed. Horizontal axis is the relative life history variable in logarithmic space. Rhombs are the means of FPKM. Colors of rhombs distinguish lineages. Error bars show standard deviation of the mean. *P*-value denotes significance of the OLS model. Median grey line is best-fit OLS line. Shaded areas indicate observed and predicted upper (95%) and lower (5%) confidence intervals. (C) Functional interaction network. Color of nodes denotes significance of the OLS model. Positively correlated genes are in red. Negatively correlated genes are in blue. Color of edges denotes type of interaction (bottom).

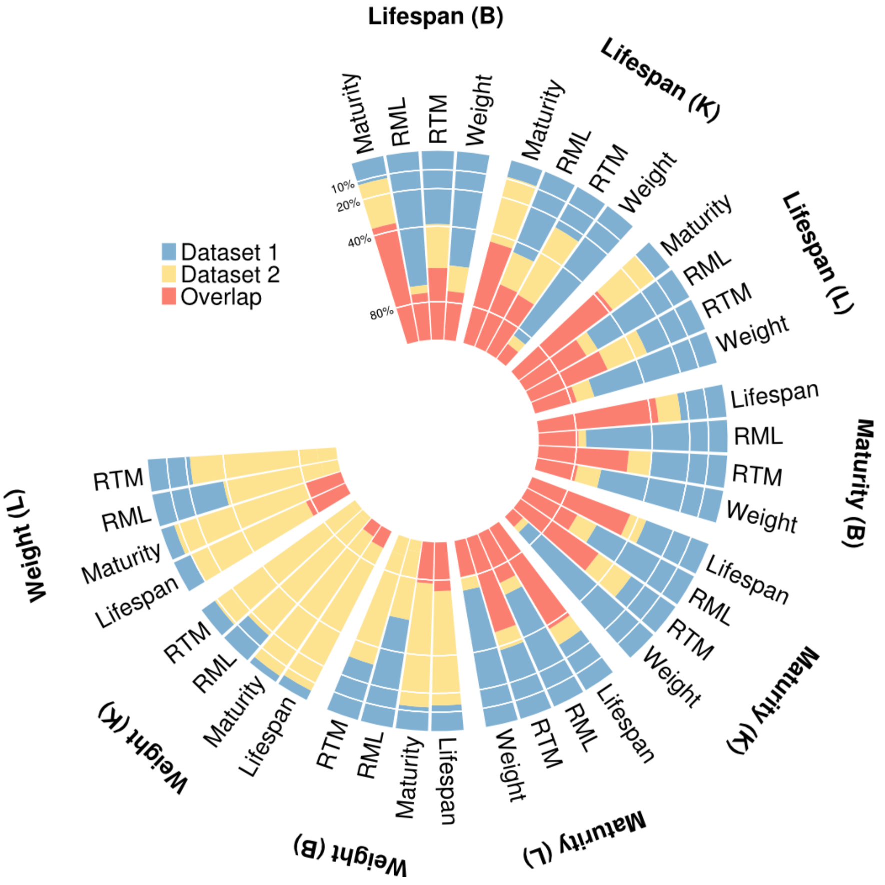

Figure S22. Overlap of genes whose expression variation associates with life histories and residuals. Each color-coded bar shows percentages of genes (scale at the beginning) unique for two data sets (blue, yellow) and percentage of common genes (red). RML, residual of maximum lifespan; RTM, residual of maturation time. L, liver; K, kidney; B, brain.

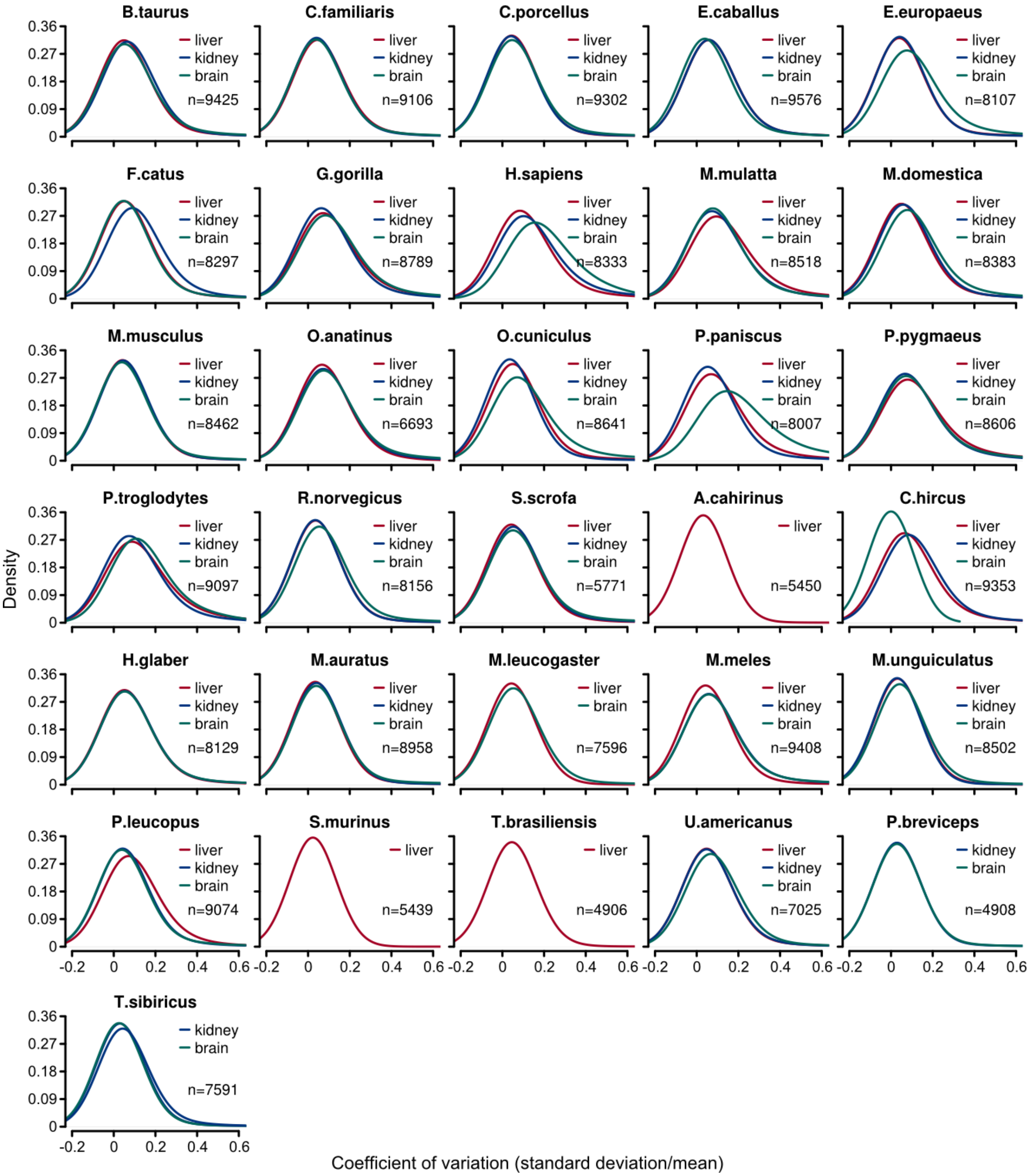

Figure S23. Within species FPKM variation. Each panel shows density of *CV* (coefficient of variation) for the liver, kidney and brain for one of the indicated species (top). *n* denotes total number of orthologs assayed in the analyses.

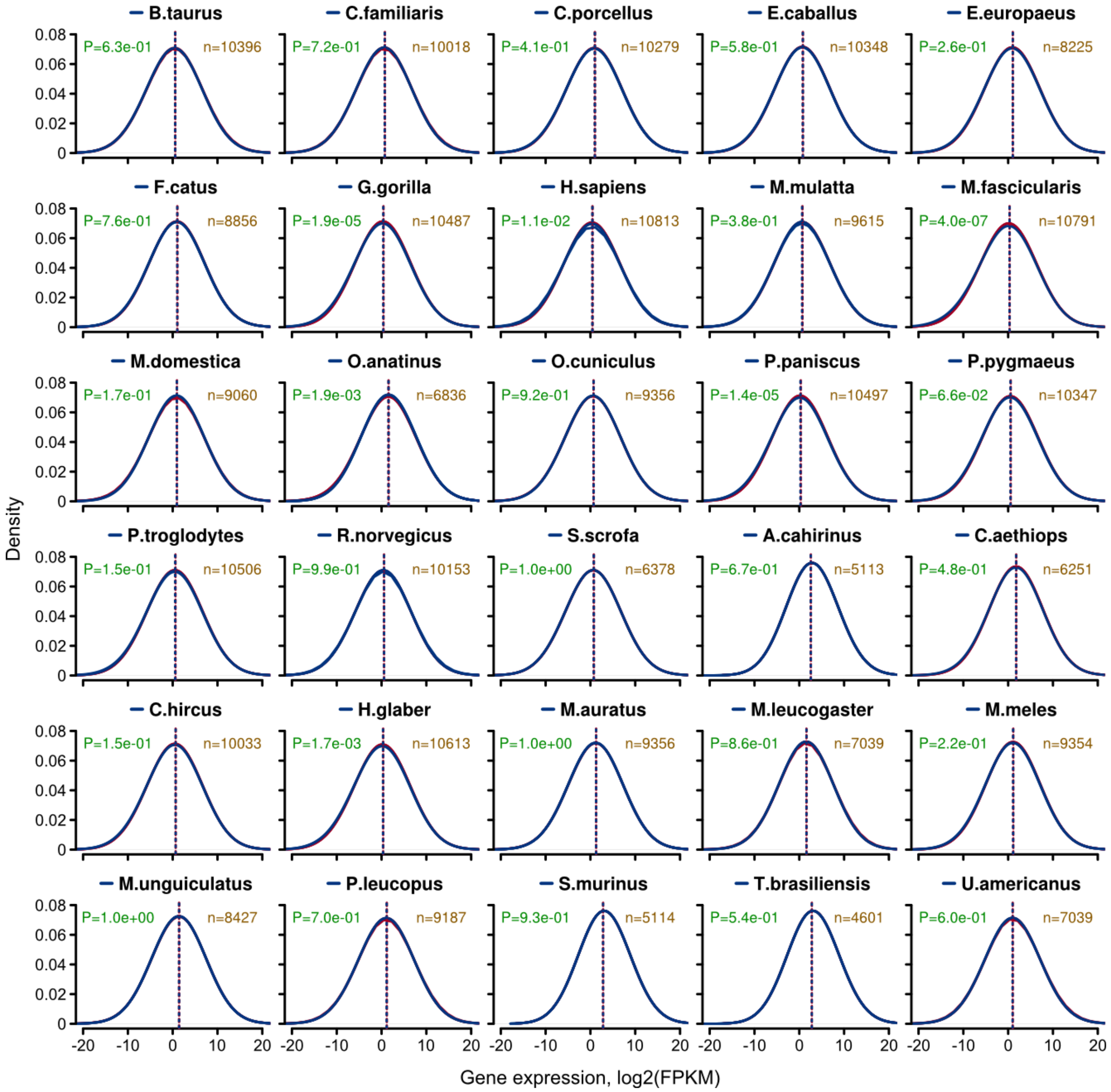

Figure S24. Normalization of liver RNA-seq samples. Each panel shows density of mouse FPKM (in red) and FPKM of one of the indicated species (in blue). *n* denotes total number of orthologous pairs. Dashed lines denote median FPKM. *P*-value denotes significance of a difference between distributions of mean FPKM of two species (two-sided Welch's test).

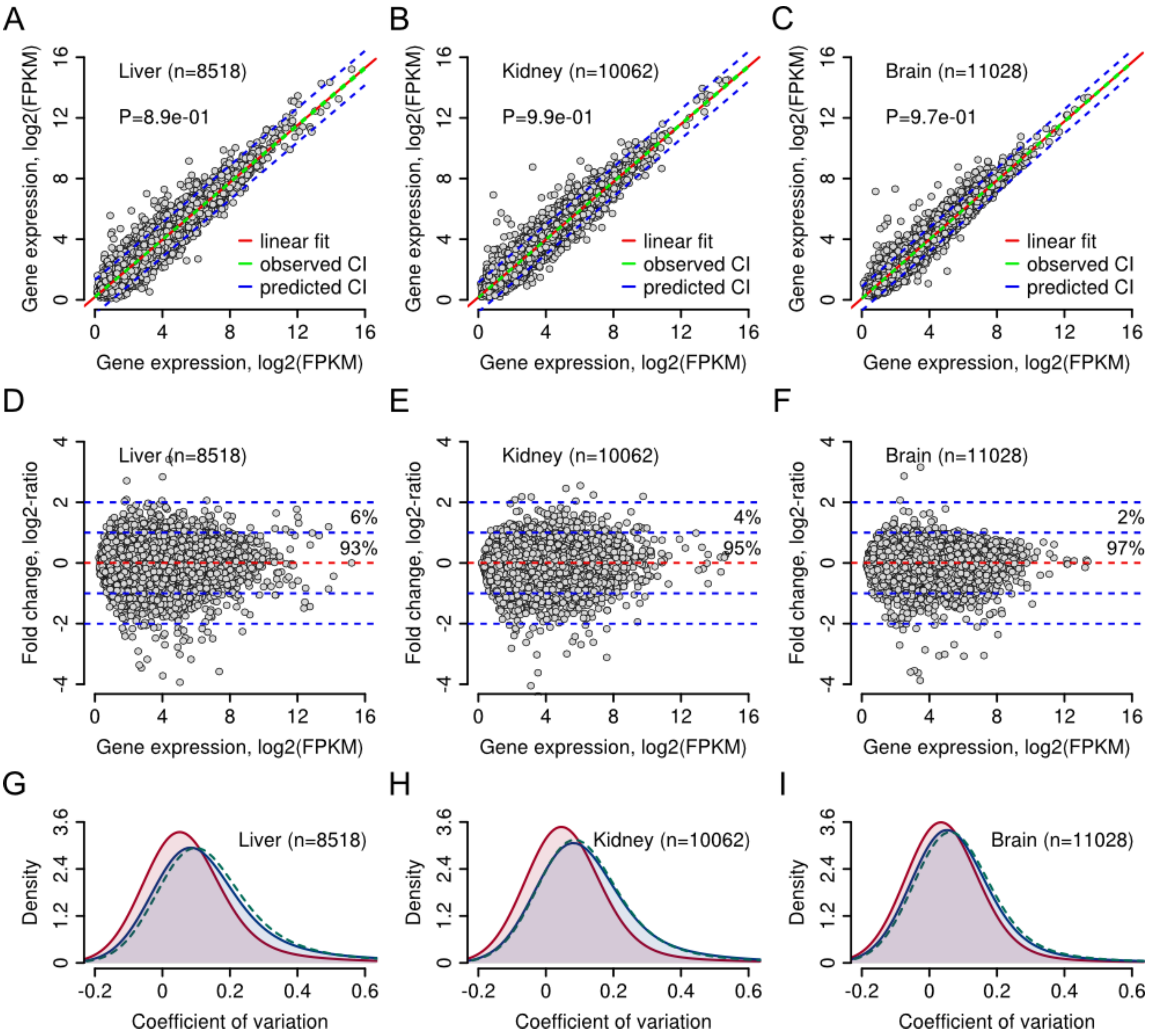

Figure S25. Variation between in-house and database RNA-seq data. (A), (B) and (C) Mean FPKM calculated from in-house RNA-seq libraries (vertical axis) and plotted against database RNA-seq data (horizontal axis, NCBI accession GSE30352) for liver, kidney and brain, respectively. Red line is best-fit regression line. CI, observed (green) and predicted (blue) upper (95%) and lower (5%) confidence intervals. *P*, K-S significance of difference (*P*-value) between distributions of FPKM. (D), (E) and (F) FPKM variation between in-house and database samples (fold change, vertical axis) plotted against mean FPKM for liver, kidney and brain (horizontal axis), respectively. Dashed blue lines with numbers indicate percentage of genes whose expression variation associate with specific fold change interval (e.g. expression variation of 93% of orthologs does not exceed log2-ratio 1 in the liver). (G), (H) and (I) Each panel shows density of *CV* (coefficient of variation) for in-house (red) and database (blue) RNA-seq data for one of the liver, kidney and brain, respectively. Dashed green line denotes density of *CV* for the combined dataset. *n* indicates total number of orthologs plotted in the graphs.

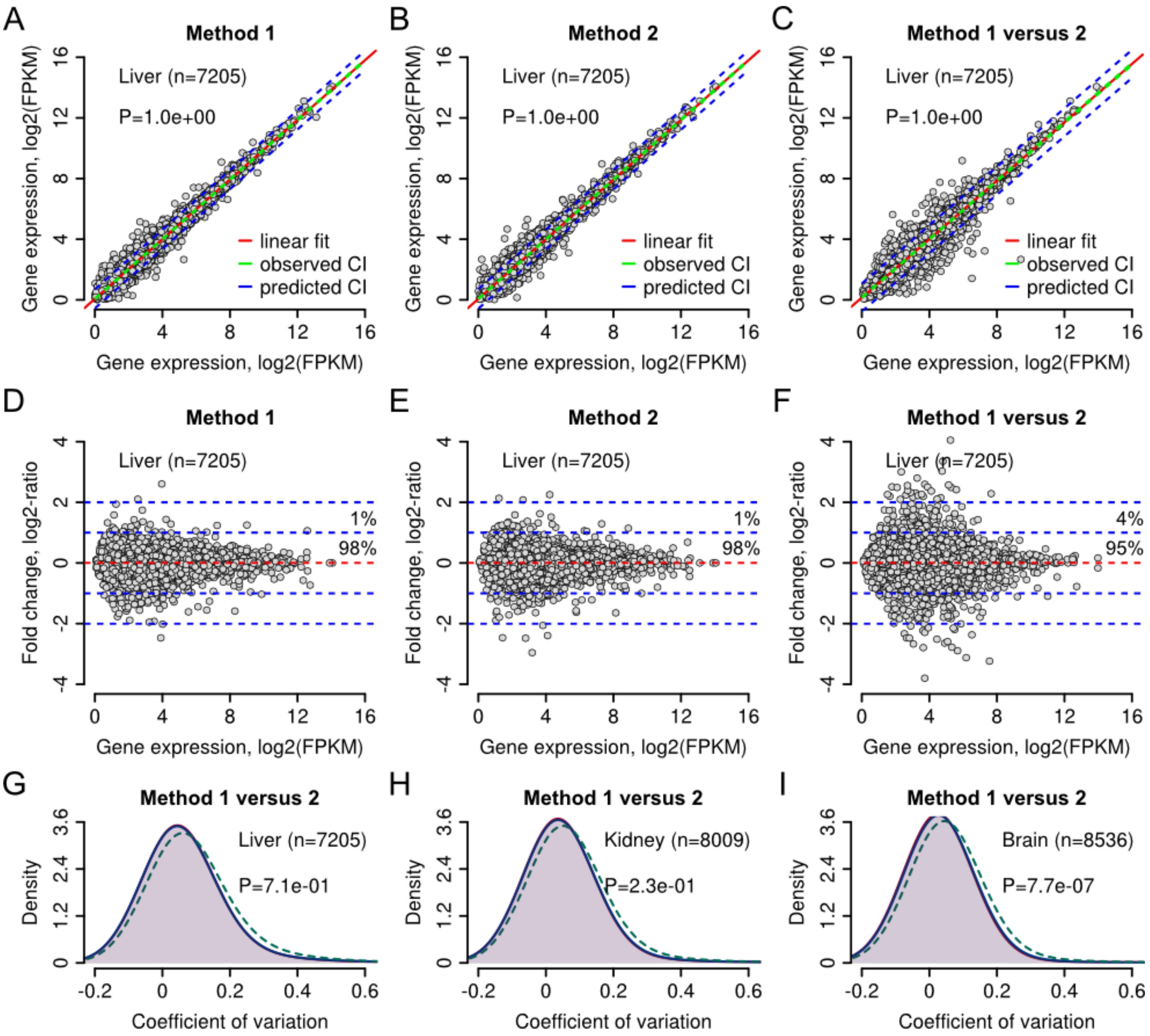

Figure S26. Variation between FPKM produced from genomic and *de novo* contig RNA-seq read alignments. RNA-seq reads (biological samples 1 and 2) were aligned with mouse genome (method 1) and *de novo* assembled transcriptome (method 2). (A) “Method 1” FPKM of sample 1 (vertical axis) plotted against “method 1” FPKM of sample 2 (horizontal axis) for liver. (B) “Method 2” FPKM of sample 1 (vertical axis) plotted against “method 2” FPKM of sample 2 (horizontal axis) for liver. (C) “Method 1” FPKM averaged between samples 1 and 2 (vertical axis) plotted against “method 2” mean FPKM (bottom axis) for liver. Red line is best-fit regression line. CI, observed (green) and predicted (blue) upper (95%) and lower (5%) confidence intervals. *P*, K-S significance of difference (*P*-value) between “method 1” and “method 2” distributions of FPKM. (D) Expression variation (fold change, vertical axis) between “method 1” FPKM of samples 1 and 2 plotted against mean FPKM for liver. (E) Expression variation (fold change, vertical axis) between “method 2” samples 1 and 2 plotted against mean FPKM for liver. (F) Expression variation (fold change, vertical axis) between “method 1” mean FPKM and “method 2” mean FPKM plotted against mean FPKM of all samples (bottom axis) for liver. Dashed blue lines with numbers indicate percentage of genes whose expression variation associate with specific fold change interval (e.g.

expression variation of 95% of orthologs does not exceed log2-ratio 1). (G), (H) and (I) Panels show density of *CV* (coefficient of variation) for “method 1” (red) and “method 2” (blue) FPKM for one of the liver, kidney or brain, respectively. Dashed green line denotes density of *CV* for the combined dataset. *n* indicates total number of orthologs plotted in the graphs. *P*, K-S significance of difference (*P*-value) between “method 1” and “method 2” distributions of *CV*.

Table S1. Classification and sampling sources of 33 mammals.

Class	Order	Family	Genus	Species	Common name	NCBI id	Abbreviation	Number of samples			Source[1]
								Liver	Kidney	Brain	
Mammalia	Euungulata	Bovidae	*Bos*	*taurus*	Domestic cattle	9913	bta	2	2	2	Study
Mammalia	Euungulata	Bovidae	*Capra*	*hircus*	Domestic goat	9925	chi	2	2	2	Study
Mammalia	Euungulata	Suidae	*Sus*	*scrofa*	Domestic boar	9825	ssc	2	2	2	Study
Mammalia	Carnivora	Canidae	*Canis*	*familiaris*	Domestic dog	9615	caf	2	2	2	Study
Mammalia	Carnivora	Felidae	*Felis*	*catus*	Domestic cat	9685	fca	3	3	3	Study
Mammalia	Carnivora	Mustelidae	*Meles*	*meles*	Asian badger	9662	mle	2	2	2	Study
Mammalia	Carnivora	Ursidae	*Ursus*	*americanus*	American black bear	9643	uam	2	2	2	Study
Mammalia	Chiroptera	Vespertilionidae	*Murina*	*leucogaster*	Greater tube-nosed bat	685731	mhi	2	1	2	Study
Mammalia	Chiroptera	Molossidae	*Tadarida*	*brasiliensis*	Brazilian free-tailed bat	9438	tbr	3	-	-	Study
Mammalia	Didelphimorphia	Didelphidae	*Monodelphis*	*domestica*	Short-tailed opossum	13616	mod	2	2	3	GSE30352
Mammalia	Diprotodontia	Petauridae	*Petaurus*	*breviceps*	Sugar glider	34899	pbr	-	2	2	Study
Mammalia	Erinaceomorpha	Erinaceidae	*Erinaceus*	*europaeus*	Western European hedgehog	9365	eeu	2	2	2	Study
Mammalia	Lagomorpha	Leporidae	*Oryctolagus*	*cuniculus*	Old World rabbit	9986	ocu	2	2	2	Study
Mammalia	Monotremata	Ornithorhynchidae	*Ornithorhynchus*	*anatinus*	Duck-billed platypus	9258	oan	4	3	4	GSE30352
Mammalia	Euungulata	Equidae	*Equus*	*caballus*	Horse	9796	eca	3	3	2	Study
Mammalia	Primates	Cercopithecidae	*Chlorocebus*	*aethiops*	Vervet	9534	cae	1	1	1	Study
Mammalia	Primates	Hominidae	*Gorilla*	*gorilla*	Gorilla	9593	ggo	2	2	2	GSE30352
Mammalia	Primates	Hominidae	*Homo*	*sapiens*	Human	9606	hsa	3	3	5	GSE30352
Mammalia	Primates	Cercopithecidae	*Macaca*	*fascicularis*	Long-tailed macaque	9541	mfa	1	1	1	GSE29629
Mammalia	Primates	Cercopithecidae	*Macaca*	*mulatta*	Rhesus monkey	9544	mmu	3	2	3	GSE30352
Mammalia	Primates	Hominidae	*Pan*	*paniscus*	Pygmy chimpanzee or bonono	9597	ppa	2	2	2	GSE30352
Mammalia	Primates	Hominidae	*Pongo*	*pygmaeus*	Orangutan	9600	ppy	2	2	2	GSE30352
Mammalia	Primates	Hominidae	*Pan*	*troglodytes*	Chimpanzee	9598	ptr	2	2	6	GSE30352
Mammalia	Rodentia	Muridae	*Acomys*	*cahirinus*	Spiny mouse	10068	aca	3	-	-	Study
Mammalia	Rodentia	Caviidae	*Cavia*	*porcellus*	Guinea pig	10141	cpo	3	3	3	Study
Mammalia	Rodentia	Bathyergidae	*Heterocephalus*	*glaber*	Naked mole-rat	10181	hgl	2	2	2	GSE30337
Mammalia	Rodentia	Muridae	*Mesocricetus*	*auratus*	Golden hamster	10036	mau	3	3	3	Study
Mammalia	Rodentia	Muridae	*Meriones*	*unguiculatus*	Mongolian gerbil	10047	mun	3	3	3	Study
Mammalia	Rodentia	Muridae	*Mus*	*musculus*	House mouse	10090	mus	3	3	3	Study
Mammalia	Rodentia	Muridae	*Peromyscus*	*leucopus*	White-footed mouse	10041	ple	2	2	2	Study
Mammalia	Rodentia	Muridae	*Rattus*	*norvegicus*	Norway rat	10116	rno	3	3	3	Study
Mammalia	Rodentia	Sciuridae	*Tamias*	*sibiricus*	Siberian chipmunk	64680	tsi	-	2	2	Study
Mammalia	Soricomorpha	Soricidae	*Suncus*	*murinus*	House shrew	9378	smu	3	-	-	Study

[1] RNA-seq libraries for Primates, Monotremata, and Didelphimorphia species were downloaded from Gene Expression Omnibus database (www.ncbi.nlm.nih.gov/geo).

Table S3. Statistics on *de novo* assembled RNA contigs for 12 mammals.

Species	Abbreviation	N25	N50	N75	Q25	Q50	Q75	Shortest	Mean	Median	Longest	N_Contigs	RNA-seq reads
Acomys cahirinus	aca	2778	1508	631	1573	4625	10938	200	860	459	14899	28805	Liver
Chlorocebus aethiops	cae	2529	1250	490	3094	9727	24956	200	740	391	15653	62901	Liver
Mesocricetus auratus	mau	3494	1201	414	7440	27588	92210	200	716	362	23906	227771	Liver, kidney, brain
Murina leucogaster	mhi	2584	1179	438	4242	13936	38339	200	698	360	17278	96004	Liver, kidney, brain
Meles meles	mle	3416	1361	467	6279	21784	66560	200	773	386	24100	170878	Liver, kidney, brain
Meriones unguiculatus	mun	3828	1829	554	3837	12128	34251	200	839	364	22871	103771	Liver, kidney, brain
Petaurus breviceps	pbr	2456	1094	421	5747	19323	53416	200	675	357	20324	129937	Kidney, brain
Peromyscus leucopus	ple	2690	813	371	9466	39410	123199	200	626	351	22839	271762	Liver, kidney, brain
Suncus murinus	smu	2872	1542	645	1486	4510	10760	200	871	458	15776	28697	Liver
Tadarida brasiliensis	tbr	2447	1214	497	1907	5979	15079	200	737	396	18342	37603	Liver
Tamias sibiricus	tsi	2615	1198	445	5363	17685	48573	200	706	364	18936	122148	Kidney, brain
Ursus americanus	uam	3017	1600	595	4283	13013	32373	200	381	381	18850	93108	Liver, kidney, brain

Note 1. N25 reports the unigene length at which 25% of the bases of the assembly were counted. Q25 reports number of contigs with sizes exceeding N25.
Note 2. N50 reports the unigene length at which 50% of the bases of the assembly were counted. Q50 reports number of contigs with sizes exceeding N50.
Note 3. N75 reports the unigene length at which 75% of the bases of the assembly were counted. Q75 reports number of contigs with sizes exceeding N75.

Table S4. Characteristics of *ab initio* predicted coding sequences and peptides for 12 mammals.

Species	Abbreviation	Predicted CDS[1]	Complete CDS[2]	% complete from total	Predicted peptide size (amino acids)			
					Shortest	Mean	Median	Longest
Acomys cahirinus	aca	8577	5317	62	66	392	306	4545
Chlorocebus aethiops	cae	12614	6845	54	66	390	299	4595
Mesocricetus auratus	mau	16326	12534	77	66	515	381	7331
Murina leucogaster	mhi	14981	8065	54	66	402	303	5225
Meles meles	mle	16938	12087	71	66	481	360	7312
Meriones unguiculatus	mun	14380	10329	72	66	498	370	7289
Petaurus breviceps	pbr	20343	11101	55	66	400	304	6415
Peromyscus leucopus	ple	17127	12150	71	66	487	363	7315
Suncus murinus	smu	8761	5039	58	66	404	305	4837
Tadarida brasiliensis	tbr	8657	4701	54	66	371	288	4545
Tamias sibiricus	tsi	20345	11690	57	66	411	312	5974
Ursus americanus	uam	14415	9304	65	66	408	311	5362

[1] Number of coding sequences predicted by Augustus software.
[2] Number of coding sequences with translation start and stop signals.

Table S5. Numbers of orthologs in COG.

Species	33-32 (1310)[1]	31-30 (2276)	29-28 (2379)	27-26 (1935)	25-24 (1754)	23-22 (1588)	21-20 (1837)	19-18 (1342)	17-16 (853)	15-14 (555)	13-12 (454)	11-10 (469)	9-8 (637)	7-6 (1042)	Total[3]
B.taurus	1301/99.3[2]	2214/97.3	2289/96.2	1833/94.7	1634/93.2	1468/92.4	1743/94.9	1230/91.7	732/85.8	443/79.8	355/78.2	313/66.7	312/49.0	363/34.8	16230
C.hircus	1305/99.6	2190/96.2	2255/94.8	1766/91.3	1590/90.6	1386/87.3	1647/89.7	1081/80.6	597/70.0	299/53.9	151/33.3	100/21.3	47/7.4	17/1.6	14431
S.scrofa	1201/91.7	1838/80.8	1853/77.9	1431/74.0	1299/74.1	1169/73.6	1430/77.8	913/68.0	573/67.2	361/65.0	304/67.0	231/49.3	267/41.9	321/30.8	13191
C.familiaris	1305/99.6	2199/96.6	2238/94.1	1784/92.2	1595/90.9	1425/89.7	1692/92.1	1146/85.4	662/77.6	414/74.6	331/72.9	304/64.8	278/43.6	397/38.1	15770
F.catus	1254/95.7	2013/88.4	1991/83.7	1553/80.3	1352/77.1	1223/77.0	1452/79.0	917/68.3	494/57.9	277/49.9	184/40.5	167/35.6	246/38.6	222/21.3	13345
M.meles	1303/99.5	2253/99.0	2306/96.9	1807/93.4	1484/84.6	1100/69.3	612/33.3	302/22.5	132/15.5	61/11.0	42/9.3	23/4.9	8/1/2003	2/0.2	11435
U.americanus	1298/99.1	2149/94.4	2009/84.4	1275/65.9	802/45.7	481/30.3	261/14.2	141/10.5	73/8.6	28/5.0	26/5.7	9/1.9	6/0.9	5/0.5	8563
M.leucogaster	1295/98.9	2144/94.2	2016/84.7	1348/69.7	800/45.6	408/25.7	186/10.1	95/7.1	52/6.1	14/2.5	8/1.8	9/1.9	3/0.5	0/0.0	8378
T.brasiliensis	1235/94.3	1613/70.9	1002/42.1	491/25.4	255/14.5	163/10.3	74/4.0	40/3.0	21/2.5	11/2.0	6/1.3	5/1.1	2/0.3	0/0.0	4918
M.domestica	1267/96.7	2080/91.4	2109/88.7	1675/86.6	1513/86.3	1333/83.9	1559/84.9	977/72.8	517/60.6	305/55.0	229/50.4	172/36.7	165/25.9	189/18.1	14090
P.breviceps	1293/98.7	2123/93.3	1986/83.5	1180/61.0	678/38.7	385/24.2	187/10.2	103/7.7	50/5.9	26/4.7	8/1.8	6/1.3	3/0.5	1/0.1	8029
E.europaeus	1258/96.0	1865/81.9	1851/77.8	1382/71.4	1258/71.7	1106/69.6	1336/72.7	795/59.2	407/47.7	223/40.2	154/33.9	150/32.0	184/28.9	183/17.6	12152
O.cuniculus	1280/97.7	2073/91.1	2075/87.2	1644/85.0	1489/84.9	1368/86.1	1598/87.0	1062/79.1	626/73.4	367/66.1	253/55.7	211/45.0	255/40.0	286/27.4	14587
O.anatinus	1183/90.3	1767/77.6	1702/71.5	1381/71.4	1238/70.6	1045/65.8	1236/67.3	622/46.3	348/40.8	196/35.3	146/32.2	116/24.7	126/19.8	129/12.4	11235
E.caballus	1309/99.9	2236/98.2	2299/96.6	1813/93.7	1647/93.9	1476/92.9	1717/93.5	1191/88.7	710/83.2	416/75.0	334/73.6	315/67.2	314/49.3	385/36.9	16162
C.aethiops	1277/97.5	2015/88.5	1743/73.3	975/50.4	548/31.2	304/19.1	132/7.2	68/5.1	29/3.4	11/2.0	10/2.2	2/0.4	1/0.2	0/0.0	7115
G.gorilla	1287/98.2	2139/94.0	2203/92.6	1750/90.4	1588/90.5	1436/90.4	1705/92.8	1206/89.9	746/87.5	439/79.1	325/71.6	321/68.4	439/68.9	662/63.5	16246
H.sapiens	1307/99.8	2237/98.3	2335/98.2	1875/96.9	1701/97.0	1540/97.0	1785/97.2	1287/95.9	773/90.6	480/86.5	379/83.5	352/75.1	469/73.6	709/68.0	17229
M.fascicularis	1301/99.3	2229/97.9	2310/97.1	1852/95.7	1684/96.0	1509/95.0	1738/94.6	1242/92.5	702/82.3	382/68.8	178/39.2	80/17.1	23/3.6	6/0.6	15236
M.mulatta	1275/97.3	2134/93.8	2181/91.7	1747/90.3	1588/90.5	1429/90.0	1686/91.8	1178/87.8	709/83.1	423/76.2	339/74.7	329/70.1	422/66.2	631/60.6	16071
P.paniscus	1301/99.3	2202/96.7	2285/96.0	1850/95.6	1640/93.5	1485/93.5	1722/93.7	1209/90.1	646/75.7	331/59.6	178/39.2	69/14.7	27/4.2	6/0.6	14951
P.pygmaeus	1295/98.9	2178/95.7	2235/93.9	1788/92.4	1622/92.5	1458/91.8	1692/92.1	1195/89.0	699/81.9	425/76.6	316/69.6	326/69.5	450/70.6	635/60.9	16314
P.troglodytes	1294/98.8	2193/96.4	2269/95.4	1813/93.7	1634/93.2	1462/92.1	1735/94.4	1233/91.9	720/84.4	433/78.0	329/72.5	318/67.8	441/69.2	640/61.4	16514
A.cahirinus	1271/97.0	1773/77.9	1110/46.7	593/30.6	342/19.5	188/11.8	87/4.7	46/3.4	24/2.8	7/1.3	5/1.1	4/0.9	3/0.5	0/0.0	5453
C.porcellus	1291/98.5	2178/95.7	2255/94.8	1779/91.9	1585/90.4	1435/90.4	1672/91.0	1125/83.8	644/75.5	369/66.5	226/49.8	215/45.8	204/32.0	195/18.7	15173
H.glaber	1303/99.5	2236/98.2	2329/97.9	1877/97.0	1670/95.2	1500/94.5	1744/94.9	1212/90.3	627/73.5	313/56.4	149/32.8	89/19.0	44/6.9	11/1.1	15104
M.auratus	1305/99.6	2249/98.8	2314/97.3	1837/94.9	1570/89.5	1141/71.9	647/35.2	298/22.2	128/15.0	56/10.1	35/7.7	8/1.7	5/0.8	0/0.0	11593
M.unguiculatus	1306/99.7	2237/98.3	2250/94.6	1743/90.1	1276/72.7	698/44.0	305/16.6	167/12.4	61/7.2	25/4.5	20/4.4	7/1.5	4/0.6	0/0.0	10099
M.musculus	1309/99.9	2234/98.2	2297/96.6	1832/94.7	1659/94.6	1497/94.3	1745/95.0	1246/92.8	736/86.3	443/79.8	336/74.0	318/67.8	317/49.8	352/33.8	16321
P.leucopus	1303/99.5	2242/98.5	2310/97.1	1812/93.6	1526/87.0	1102/69.4	599/32.6	275/20.5	129/15.1	66/11.9	26/5.7	15/3.2	8/1/2003	0/0.0	11413
R.norvegicus	1268/96.8	2109/92.7	2106/88.5	1702/88.0	1546/88.1	1397/88.0	1639/89.2	1161/86.5	677/79.4	404/72.8	309/68.1	306/65.2	310/48.7	372/35.7	15306
T.sibiricus	1301/99.3	2204/96.8	2170/91.2	1503/77.7	854/48.7	471/29.7	217/11.8	106/7.9	42/4.9	25/4.5	15/3.3	7/1.5	1/0.2	1/0.1	8917
S.murinus	1263/96.4	1814/79.7	1106/46.5	598/30.9	342/19.5	166/10.5	77/4.2	34/2.5	20/2.3	8/1.4	3/0.7	5/1.1	1/0.2	2/0.2	5439

[1] Column headers denote the numbers of individual species in COG and total number of COGs of a given size (in brackets).
[2] Cells show the numbers of orthologs for each individual species and percentage from total number of COGs of a given size.
[3] Total numbers of orthologs for each particular species.

Table S6. Life histories of 33 mammals.

Common name	Abbreviation	Gestation period (days)[1]	Weaning time (days)[1]	Adult weight (grams)[1]	Growth[2]	Time to maturity (days)[1]	Residual of *tsex*	Maximum lifespan (days)[1]	Residual of *tmax*	Oxygen consumption (ml/gram)[3]
Domestic cattle	bta	277	180	540000	0.0031	365	0.254	7997.15	0.604	0.17
Domestic goat	chi	155	160	61000	0.0041	685	0.763	7358.4	0.761	0.19
Domestic boar	ssc	115	56	180000	0.0095	768	0.678	7665	0.678	0.11
Domestic dog	caf	63	56	20000	0.0244	510	0.723	5840	0.709	0.333
Domestic cat	fca	65	56	3900	-	210	0.423	6205	0.953	0.71
Asian badger	mle	49	90	13000	0.0196	365	0.568	5913	0.763	0.27
American black bear	uam	220	198	154250	0.0029	1278	1.166	12410	1.123	0.36
Greater tube-nosed	mhi	-	-	12.75	-	-	-	3285	1.148	1.42
Brazilian free-tailed	tbr	78	42	12.5	0.112	547	3.799	3650	1.280	1.51
Short-tailed	mod	15	53	105	-	122	0.536	1861.5	0.481	0.57
Sugar glider	pbr	16	122	110	0.0188	456	1.982	5110	1.310	0.69
Hedgehog	eeu	35	42	750	-	253	0.727	4270.5	0.831	0.45
Old World rabbit	ocu	30	26	1800	0.0228	240	0.571	3285	0.564	0.57
Duck-billed platypus	oan	17	106	1250	-	548	1.411	6205	1.122	0.31
Horse	eca	337	274	350000	-	973	0.744	16790	1.350	0.25
Vervet	cae	162	182	5620	-	1825	3.400	11242	1.637	0.306
Gorilla	ggo	256	834	139842	0.0008	4015	3.742	16060	1.473	0.168
Human	hsa	280	639	70000	0.0005	5110	5.528	36500	3.699	0.21
Long-tailed	mfa	165	242	6362	-	1544	2.800	13505	1.932	0.298
Rhesus monkey	mmu	165	292	8235	0.0012	2007	3.443	14600	2.013	0.37
Pygmy chimpanzee	ppa	232	635	39925	-	3194	3.900	18250	2.005	0.203
Orangutan	ppy	249	1003	64475	0.0009	2555	2.813	21425.5	2.197	0.3
Chimpanzee	ptr	229	1111	44983	0.0007	2920	3.475	19491	2.105	0.26
Spiny mouse	aca	38	14	45	0.0147	59	0.311	2153.5	0.628	1.1
Guinea pig	cpo	68	18	728	0.0106	76	0.220	4380	0.856	0.55
Naked mole-rat	hgl	70	36	35	0.0046	365	2.031	10329.5	3.123	0.66
Golden hamster	mau	16	20	105	0.0574	48	0.211	1423.5	0.367	1.5
Mongolian gerbil	mun	25	24	53.2	0.0324	36	0.183	1387	0.395	1.15
House mouse	mus	19	22	20.5	0.0298	42	0.262	1460	0.477	1.667
White-footed mouse	ple	26	22	23	0.0456	44	0.268	2883.5	0.926	2.2
Norway rat	rno	21	25	300	-	70	0.245	1825	0.405	1.32
Siberian chipmunk	tsi	35	40	85	-	350	1.609	3504	0.933	1.25
House shrew	smu	30	19	45	0.0643	36	0.190	1168	0.341	1.97

[1] Life histories were collected from published literature and AnAge database (www.genomics.senescence.info).
[2] An estimate of Gompertz function defining the fraction of body weight accumulating per day (data from AnAge).
[3] Oxygen consumption is the volume of oxygen (ml) consumed in 1 hour. Here, the data are adjusted by species body weight (grams).

Table S7. Lambda parameter estimates and associated statistics for life histories.

Life history	λ	lnL (λ)[1]	lnL (λ=0)[2]	lnL (λ=1)[3]	*P* (λ=0)[4]	*P* (λ=1)[5]
Gestation period	0.65	-44.7	-46.6	-47.3	4.90e-02	2.30e-02
Weaning time	0.37	-49.9	-50.6	-57.6	2.40e-01	8.60e-05
Adult weight	0.39	-79.3	-79.7	-84.1	3.50e-01	2.00e-03
Growth rate	1	-40.6	-49.6	-40.6	2.10e-05	1.00e+00
Time to maturity (*tsex*)	0.72	-51.2	-54.7	-54.1	8.30e-03	1.60e-02
Residual of *tsex*	0.72	-45.6	-47.5	-47.6	5.00e-02	4.70e-02
Maximum lifespan (*tmax*)	0.65	-42.4	-44	-44.9	7.70e-02	2.50e-02
Residual of *tmax*	0.64	-33.9	-34.2	-34.8	4.90e-01	1.90e-01
Oxygen consumption	0.41	-39.4	-40	-42.8	2.70e-01	9.00e-03

[1] log-likelihoods of the λ model.
[2] log-likelihoods of the “noise” model (phylogenetic tree with λ=0).
[3] log-likelihoods of the Brownian motion (BM) model.
[4] Significance of difference of the λ model from noise (LRT).
[5] Significance of difference of the λ model from the BM (LRT).

Table S8. Statistics for genes whose expression variation is consistent with the BM model.

Model[1]	Liver (*n* = 12835)[2]		Kidney (*n* = 13859)		Brain (*n* = 14091)		Combined[4]
	Nb. of genes[3]	% from total	Nb. of genes	% from total	Nb. of genes	% from total	
BM ($P < 0.05$)	837 (518)	6.5 (4.0)	1994 (1190)	14.4 (8.6)	2346 (1555)	16.6 (11.0)	4182 (76)
BM ($P < 0.01$)	625 (412)	4.9 (3.2)	1637 (1002)	11.8 (7.2)	1976 (1340)	14.0 (9.5)	3471 (50)

[1] *P* denotes significance of a difference from randomly distributed value (no effect of phylogeny).
[2] *n* denotes total number of orthologous groups assayed in the analysis.
[3] Number of significant genes and number of genes specific for an organ (in brackets).
[4] Number of significant genes identified in three organs and inter-organ overlap (in brackets).

Table S9. Statistics for genes whose expression variation is consistent with the OU model.

Taxonomic group	Liver (n = 6446)[1]		Kidney (n = 5424)		Brain (n = 5442)		Combined[4]
	Nb. of genes[2]	% from total	Nb. of genes	% from total	Nb. of genes	% from total	
Primates	254 (208)	3.9 (3.2)	183 (137)	3.4 (2.5)	204 (168)	3.7 (3.1)	574 (6)
Hominidae	145 (124)	2.2 (1.9)	160 (117)	2.9 (2.1)	310 (266)	5.7 (4.9)	558 (6)
Rodentia	160 (137)	2.5 (2.1)	114 (90)	2.1 (1.6)	161 (141)	3.0 (2.6)	401 (1)
Eumuroida	106 (91)	1.6 (1.4)	100 (84)	1.8 (1.5)	114 (99)	2.1 (1.8)	297 (0)
Chiroptera	87 (76)	1.3 (1.2)	148 (132)	2.7 (2.4)	52 (40)	1.0 (0.7)	266 (3)
Laurasiatheria	71 (64)	1.1 (1.0)	58 (49)	1.1 (0.9)	105 (97)	1.9 (1.8)	222 (0)
Rodentia and Lagomorpha	87 (77)	1.3 (1.2)	78 (67)	1.4 (1.2)	62 (55)	1.1 (1.0)	213 (0)
Euungulata and Carnivora	58 (48)	0.9 (0.7)	70 (61)	1.3 (1.1)	55 (52)	1.0 (0.9)	172 (0)
Chiroptera, Laurasiatheria, Euungulata and Carnivora	70 (63)	1.1 (1.0)	47 (38)	0.9 (0.7)	48 (40)	0.9 (0.7)	153 (0)
Lagomorpha	47 (41)	0.7 (0.6)	33 (23)	0.6 (0.4)	65 (55)	1.2 (1.0)	131 (2)
Hystricognathi	49 (45)	0.8 (0.7)	34 (30)	0.6 (0.5)	44 (40)	0.8 (0.7)	121 (0)
Chiroptera, Euungulata and Carnivora	41 (38)	0.6 (0.6)	45 (40)	0.8 (0.7)	41 (38)	0.8 (0.7)	121 (1)
Euungulata	35 (31)	0.5 (0.5)	29 (25)	0.5 (0.5)	52 (51)	1.0 (0.9)	111 (1)
Cercopithecidae	41 (37)	0.6 (0.6)	38 (32)	0.7 (0.6)	36 (30)	0.7 (0.5)	106 (2)
Carnivora	42 (36)	0.7 (0.6)	43 (36)	0.8 (0.7)	27 (26)	0.5 (0.5)	105 (0)
Combined[3]	1293 (1166)	19.8 (17.3)	1180 (961)	21.6 (17.7)	1376 (1198)	25.4 (22.0)	3551 (22)

[1] *n* denotes total number of orthologous groups assayed in the analysis.
[2] Number of genes identified in the organ and number of genes specific for an organ (in brackets).
[3] Number of genes identified in the organ for all lineages and number of genes specific for an organ (in brackets).
[4] Number of genes identified in three organs for all lineages and inter-organ overlap (in brackets).
